# Meta-Analysis of ALS Post-Mortem Cerebral Cortex Transcriptome Studies Identifies Robust Shifts in Gene Expression Despite Significant Between-Study Heterogeneity

**DOI:** 10.3390/brainsci16101120

**Published:** 2026-10-16

**Authors:** William R. Swindell, Aminah Ali, Alfredo Iacoangeli, Salman Bhai

**Affiliations:** 1 Department of Internal Medicine, Division of Hospital Medicine, University of Texas Southwestern Medical Center, Dallas, TX 75390, USA; 2 Department of Biostatistics & Health Informatics, Institute of Psychiatry Psychology & Neuroscience, King’s College London, 16 De Crespigny Park, London SE5 8AB, UK; aminah.2.ali@kcl.ac.uk (A.A.); alfredo.iacoangeli@kcl.ac.uk (A.I.); 3 Department of Basic and Clinical Neuroscience, Maurice Wohl Clinical Neuroscience Institute, Institute of Psychiatry, Psychology and Neuroscience, King’s College London, London SE5 9RT, UK; 4 NIHR Maudsley Biomedical Research Centre (BRC), South London and Maudsley NHS Foundation Trust and King’s College London, London SE5 8A, UK; 5 Perron Institute for Neurological and Translational Science, Ground RR Block QE II Medical Centre Ralph & Patricia Sarich Neuroscience Building, 8 Verdun St, Nedlands, WA 6009, Australia; 6 Department of Neurology, University of Texas Southwestern Medical Center, Dallas, TX 75390, USA; salman.bhai@utsouthwestern.edu

**Keywords:** expression profiling, glial cells, meta-regression, motor neuron disease, pyramidal layer, spatial transcriptomics, white matter

## Abstract

**Highlights:**

**What are the main findings?**

- Gene expression changes in the ALS cortex exhibit significant between-study heterogeneity (one-third of genes evaluated).
- We nonetheless identified 542 DEGs with consistently altered expression (FDR < 0.05 with SMD ≥ 0.20 or SMD ≤ −0.20).

**What are the implications of the main findings?**

- Meta-analysis DEGs link mRNA processing, interneurons, microglia, astrocytes and oligodendrocytes to ALS pathology.
- Meta-analysis provides a valuable tool for identifying reproducible findings and sources of between-study heterogeneity.

**Abstract:**

**Background:** ALS is characterized by motor neuron failure with potential neurocognitive comorbidity. Cortical neurons, and their support cells, exhibit pathologic features, some of which have been identified in post-mortem transcriptome studies of the cortex from ALS patients and control subjects. These studies, however, have utilized varying technologies with few attempts to reconcile findings. **Methods:** This study used meta-analysis to evaluate differential gene expression (ALS vs. control) from 16 prior transcriptome studies utilizing multiple technologies (microarray, bulk RNA-seq or snRNA-seq). Normalization with GC content correction was used to mitigate downstream biases impacting differential expression. **Results:** Approximately one-third of protein-coding genes (5930 of 16,579) exhibited significant between-study effect size heterogeneity (FDR < 0.05), which was frequently associated with variation in average post-mortem interval. Nonetheless, we identified 269 and 273 differentially expressed genes (DEGs) with relatively consistent increased or decreased expression in the ALS cortex, respectively (FDR < 0.05 with SMD ≥ 0.20 or SMD ≤ −0.20). ALS-increased DEGs were associated with transcription (RNA polymerase), cell signaling (TGF-beta, PI3K), interneurons, microglia, endothelial cells, and deep laminar layers (L5, L6). ALS-decreased DEGs were associated with transcription (RNA polymerase), glial cells (astrocytes, oligodendrocytes) and the L1 layer. There was significant overlap between DEGs from the ALS cortex and those previously identified from ALS spinal cord and blood. **Conclusions:** These findings highlight signaling pathways, cell types and cortical layers associated with robust changes in ALS cortex gene expression. Our results illustrate the importance of meta-analysis as a tool for understanding between-study heterogeneity and identifying repeatable findings across technologies.

## 1. Introduction

ALS is characterized by upper and lower motor neuron failure leading to progressive loss of motor function with a poor prognosis [1]. Functional decline is associated with loss of cortical motor neurons, although there may be broader cerebral involvement with deficits in executive function and frontotemporal dementia (FTD) [2]. These aspects of ALS progression are linked to dysfunction and hyperexcitability of cortical circuits that drive disease progression [3]. Though FDA-approved therapies exist for ALS, most have limited efficacy with regard to improved survival [4]. Patients carrying *SOD1* mutations, however, have shown improved biomarker profiles following antisense oligonucleotide therapy [5,6]. This has raised hope that gene-based therapies can provide a path towards effective ALS treatments [7], despite decades of limited drug development progress without individualized therapies [8]. High-throughput omics-based technologies, such as single-cell or single-nucleus RNA-sequencing (scRNA-seq or snRNA-seq), are promising approaches for disease-associated pathway discovery [9]. Processes linked to such pathways within the brain may in fact be connected to peripheral mechanisms [10] and provide targets for ALS drug development [11,12].

ALS post-mortem tissues provide a window into end-stage disease and have been studied using transcriptomic workflows [13]. This has revealed transcriptional dysregulation in the ALS brain with concordance across regions, including the cortex, hippocampus and cerebellum [14]. Similar expression shifts have been described in post-mortem cortexes from ALS and FTD patients and from those with sporadic and familial (*C9orf72*) disease [15,16]. These shifts have been linked to diverse processes, including histone regulation [17], alternative splicing [14], RNA metabolism [18], peroxisomes [19], proteostasis [20], unfolded protein response [21] and neuroinflammation [22]. Transcriptomic studies have also uncovered important nuances. More profound shifts in cortical pathways, for example, have been observed in males compared to females [23]. ALS-associated expression shifts have also been anatomically localized (e.g., layer 5 of the motor cortex) [24]. Many studies have probed *within-study* heterogeneity to identify subtypes (or substages) from ALS patient transcriptomic signatures [11,25,26,27,28,29,30]. A prior analysis, for example, showed changes related to oxidative and proteotoxic stress in most patients (61%), with others exhibiting shifts related to glial activation (19%) or TARDBP/TDP-43 pathology (20%) [25].

Transcriptomic studies of ALS brain have utilized diverse profiling technologies over decades, starting with microarrays [31], and then advancing to bulk RNA-seq and scRNA-seq or snRNA-seq [32]. Additionally, spatial transcriptomic studies have enabled localization of gene expression changes within their histological context [24]. Data from these platforms have accumulated within repositories [33,34,35,36,37], although they have varied with regard to quality and sample size, with each technology subject to specific pitfalls and biases. scRNA-seq or snRNA-seq, for example, may lack precision and accuracy when source RNA is degraded or if the number of cells per cell type per individual is low [38]. This can give rise to *between-study* heterogeneity that has, so far, been poorly investigated. Meta-analysis, however, provides a useful framework to characterize such heterogeneity and identify moderator variables (technical or biological) accounting for study-to-study variability [39,40,41]. Additionally, meta-analysis can integrate findings across studies to highlight *reproducible* findings [42], which may differ from those associated with extremely low *p*-values within any single study [43,44]. This can sharpen the focus on the best drug targets, guarding against the pursuit of non-reproducible findings and misspent investigational effort [45,46,47].

This paper reports findings from a meta-analysis of transcriptome datasets from the post-mortem cortexes of ALS patients and neurologically normal control subjects. The analysis integrates results from multiple generations of expression profiling technology spanning 18 years (2007–2025), including microarray [48,49,50], bulk RNA-seq [14,16,23,25,29,51,52] and snRNA-seq [15,20,26,53,54,55]. Data are normalized with GC content bias correction [56,57], and studies with multiple samples per patient are analyzed using linear mixed-effects models [58]. Sources of between-study heterogeneity are identified, and linear meta-regression modeling [59] is used to evaluate the degree to which moderator variables are associated with such heterogeneity. A random-effect meta-analysis model is applied to integrate effect size estimates across studies, leading to the identification of differentially expressed genes (DEGs) with the highest between-study reproducibility. These DEGs are analyzed to assess for enriched gene annotations, overlap with genes near ALS susceptibility loci [60], cell type-specific marker genes from existing databases [61,62,63], and marker genes linked to snRNA-seq cell clusters from post-mortem human brains [64,65]. This work builds on prior studies [66,67] to understand between-study variation in transcriptomic data, and spotlight reproducible findings in ALS post-mortem tissue research.

## 2. Materials and Methods

### 2.1. Study Selection

The meta-analysis included 16 datasets generated from gene expression profiling of post-mortem cortex samples from ALS patients (sporadic or familial) and control subjects without known neurological disease (Table 1). Expression profiling technologies included microarray, bulk RNA-seq and snRNA-seq. Candidate datasets were identified by querying gene expression data repositories, including the Gene Expression Omnibus (GEO) [33], Sequence Read Archive (SRA) [34], ArrayExpress [35], European Genome-phenome Archive (EGA) [36], Synapse [68], National Institute on Aging Genetics of Alzheimer’s Disease Data Storage Site (NIAGADS) [37] and DNA Data Bank of Japan (DDBJ) [69]. We additionally queried primary literature databases [70,71] to identify any datasets described in research publications without submission of raw data to one of the above-mentioned repositories for gene expression datasets.

We identified 25 candidate datasets (16 included, 9 excluded) with release prior to our cutoff date of 1 January 2026. Two datasets (GSE126542, GSE219278) were excluded because cells had been sorted prior to expression profiling such that it would be difficult to compare results to other datasets [20,72,73]. Another dataset (GSE179819) was excluded because only miRNA was isolated and quantified [74]. Three datasets (GSE124439, GSE153960, GSE290359) were excluded because samples were subsets of those within an included dataset [25,73,75,76,77]. Three datasets were excluded because data had not been submitted to a public database and was no longer available upon request from corresponding authors [78,79,80].

### 2.2. Dataset A (GSE4595)

Two-color microarray data were generated from motor cortexes of ALS patients (*n* = 11) and CTL subjects (*n* = 9) [48]. Gene expression was profiled using the Agilent-012391 Whole Human Genome Oligo Microarray G4112A (GPL1708) platform. Raw data files were not available from the Gene Expression Omnibus. Available series matrix files included log_2_-transformed ratios (*M*), representing ratios of signal intensities from two channels, including Cy5-labeled sample cRNA and Cy3-labeled reference cRNA (co-hybridized to the same array) (Figure S1A). *M* values were available for 41,675 probes and 20 samples (2.8% of values were missing). Global median location normalization was performed to equalize the median *M* across arrays (Figure S1B) [81]. Quantile normalization was then performed to ensure the comparability of *M* distributions (R package: limma; function: normalizeBetweenArrays) (Figure S1C).

Following these steps, inspection of array pseudoimages showed that *M* values for several array samples were associated with spatial bias (Figure S2A). Spatial correction was performed by fitting a loess model with *x* and *y* coordinates as predictor variables, then utilizing residuals from this loess fit as spatially adjusted *M* values (Figure S1D) [66]. Following this step, little or no spatial bias was discernable (Figure S2B). A persistent region of spatial bias was noted with respect to one array sample (GSM102840, ALS-58M-04) (Figure S2B). This array region was associated with 967 probes, which were treated as missing values in subsequent analyses.

Of 41,675 probes, some were associated with the same human gene. To limit redundancy, probes associated with the same human gene were filtered, preferentially retaining the probe for which the average |*M*| was highest across the 20 array samples. This yielded 18,841 probes, each corresponding to a unique human gene, of which 13,353 were protein-coding. Further analyses were performed using 13,327 of these probes, with fewer than 50% missing values in the ALS and CTL groups, respectively. Samples associated with different sexes or ages overlapped within two-dimensional principal component (PC) space (Figure S3A,B,D,E). One sample was a potential outlier (GSM102844, ALS_75M_06) but was retained since it was otherwise unremarkable based on technical criteria (Figures S1 and S2). ALS and CTL subjects did not differ significantly with respect to sex or age at death (Figure S3C,F). Effect sizes associated with the disease status (ALS vs. CTL) were estimated using linear models, with normalized and spatially corrected *M* values as the response variable and the disease status as a categorical variable (ALS vs. CTL). Additional covariates included sex (categorical) and age at death (continuous) (R package: stats; function: lm).

### 2.3. Dataset B (E-MTAB-1925)

Post-mortem samples were obtained from motor cortexes of 3 ALS patients (*C9ORF72* genotype) and 3 CTL subjects [49]. Gene expression was measured using the Affymetrix human 1.0 ST exon array platform. Raw CEL files were obtained from ArrayExpress [35]. Inspection of array pseudoimages did not reveal any spatial artifact (Figure S4A). Probe-level modeling was used to calculate normalized unscaled standard error (NUSE) and relative log expression (RLE) metrics [82]. NUSE and RLE distributions were shifted upward for one sample (CTL-M-3, R995-MC-Control) but similar among other samples (Figure S4B,C).

Normalization was performed using the single-channel array normalization (SCAN) algorithm with correction for GC-content bias (R package: SCAN.UPC; function: SCAN) [56]. Results from this approach were compared to those from data normalized using the robust multichip average (RMA) algorithm (R package: oligo; function: rma) [83]. NetAffx annotations were obtained for 17,638 of 22,011 transcript clusters (R package: oligo, function: getNetAffx) [84]. These were filtered to retain 14,295 clusters linked uniquely to an official human gene symbol. In some cases, more than one cluster was associated with the same human gene [85]. Such clusters were filtered to remove redundancy, retaining those with the highest average normalized expression across samples. This yielded 14,133 clusters, each associated with a unique human gene. Of these, further analyses were performed using 13,631 clusters associated with a protein-coding human gene.

For any given sample, a protein-coding gene was considered to have detectable expression if the normalized signal intensity was greater than the 15th percentile (relative to all other protein-coding genes). Based on this definition, the disease status effect size was estimated for 12,379 protein-coding genes with detectable expression with respect to at least 1 of 3 ALS samples or 1 of 3 CTL samples. None of the 12,379 genes were associated with outlier expression values (i.e., absolute z-score ≥ 3). Effect sizes were estimated using linear models with the disease status (ALS vs. CTL) as the primary independent variable along with sex (categorical) and age at death (continuous) as covariates (R package: stats; function: lm).

### 2.4. Dataset C (E-MTAB-2325)

Data were generated from motor cortexes of sporadic ALS patients (*n* = 31) and CTL subjects (*n* = 10) [50]. Gene expression was profiled using the Agilent 4x44K whole-human-genome oligo microarray platform. A data matrix with raw signal intensities for 41,000 probes was obtained from ArrayExpress [35]. Original data files generated by GenePix data-extraction software were not available. Inspection of raw signal intensities identified one sample (ALS-61F-22, 69bis 09-13-07) with low probe intensities relative to other samples (Figure S5A). Since this sample was also associated with a prolonged post-mortem interval (10 h), it was excluded from further analyses due to concern for RNA degradation leading to low signal intensities (Figure S5D–F). Raw signal intensities for all other samples were background-corrected using the normal-exponential convolution model (Figure S5B,E; R package: limma, function: backgroundCorrect) [86], followed by log_2_-transformation of corrected intensities with subsequent quantile normalization (Figure S5C,F; R package: limma, function: normalizeBetweenArrays).

Gene annotations were available for 29,668 of the 41,000 probes. To limit redundancy among the 29,668 probes, those associated with the same gene symbol were filtered, retaining probes with the highest average signal intensity across all samples, which yielded 18,804 probes uniquely associated with a distinct human gene. Of these, 17,207 probes were linked to a protein-coding human gene. All of these probes had detectable expression with respect to at least 15% of ALS samples or 15% of CTL samples. A gene was considered to have detectable expression if its normalized expression signal was greater than the 15th percentile relative to all other genes. The effect size associated with disease status was estimated for these 17,207 genes using linear models, with disease status as the primary independent variable, along with sex (categorical) and age at death (continuous) as covariates.

### 2.5. Dataset D (GSE67196)

Bulk RNA-seq data were generated from frontal cortexes of sporadic or familial (*C9ORF72*) ALS patients (*n* = 18) and CTL subjects (*n* = 9) [51,87]. Paired-end sequencing reads (100 bp) were generated using the Illumina HiSeq 2000 platform (Illumina, San Diego, CA, USA). Raw sequence files (fastq format) were obtained from the Sequence Read Archive (SRP056477) [34]. FastQC was used to evaluate reads and extract quality control statistics [88]. There was an average of 36.6 million reads among samples prior to any filtering (Figure S6A). TrimGalore was used to trim the Illumina universal adapter sequence and filter out sequences with a Phred score below 20 (Illumina 1.9 encoding) [89]. Trimmed reads with a length < 50 bp were removed. A BBTools script (bbduk.sh) was used to filter out reads mapping to a database of rRNA sequences (rRNA databases v4 database) [90].

Following these filtering steps, there remained an average of 31.0 million reads per sample (Figure S6B). Filtered reads were mapped to the GRCh38/hg38 genome using the STAR algorithm [91]. Stringtie was used to calculate transcript abundance measures such as FPKM (fragments per kilobase of transcript per million fragments mapped) and TPM (transcripts per million) [92]. Gene counts were calculated using a script (prepDE.py3) within the Stringtie distribution. RSeQC was used to evaluate BAM alignment files and calculate post-mapping quality control metrics [93]. An average of 95.5% of reads were mapped among samples (Figure S6C), with 92.9% of reads mapped uniquely (Figure S6D). An average of 91.9% and 89.3% of reads were assigned to intragenic and exonic sequences, respectively (Figure S6E,F), with only 7% of reads mapped to the ribosomal sequence (Figure S6G). Reads were mapped to 19,913 protein-coding genes within the GRCh38/hg38 genome. On average, 67.2% of such genes had detectable expression among samples (Figure S6H), where detectable expression was defined using multiple measures of gene abundance (FPKM > 0.3, TPM > 0, count > 0).

Disease status effect sizes were estimated for 14,210 protein-coding genes, with detectable expression in at least 15% of ALS samples (≥3 of 18) or at least 15% of CTL samples (≥2 of 9). Raw counts were normalized using conditional quantile normalization (CQN) with correction for GC content bias (R package: cqn; function: cqn) [57]. When samples were plotted with respect to the first two PC axes, there was no clear separation of samples differing by sex or age at death (Figure S7A,B,D,E). ALS and CTL groups did not significantly differ with respect to the sex ratio (*p* > 0.05, Figure S7C), although the age at death was significantly lower among ALS subjects (*p* < 0.01, Figure S7F). To estimate the disease status effect size, transformed and normalized expression values (log_2_ scale) were treated as linear model dependent variables, with coefficient estimates calculated for the disease status (categorical), sex (categorical) and age at death (continuous) (R package: stats; function: lm).

### 2.6. Dataset E (GSE122649)

Bulk RNA-seq was performed on motor cortex samples from 13 sporadic ALS patients and 12 CTL subjects [25]. Two replicate samples were available from each individual. Fastq files with single-end reads (76 bp) were obtained from the Sequence Read Archive (SRP169128) [34]. Reads were generated using the Illumina NextSeq 500 platform. Reads were quality control-processed and mapped following the protocol described above (dataset *D*). There was an average of 63.3 million reads per sample (Figure S8A), with 58.6 million reads/sample remaining after quality filtering (Figure S8B). An average of 94.2% of reads were successfully mapped (Figure S8C), with 87% of reads mapped uniquely (Figure S8D). Nearly all reads (97.1%) were assigned to the intragenic sequence (Figure S8E), and 51.3% were mapped to exons (Figure S8F). An average of 2.3% of reads mapped to ribosomal sequences (Figure S8G), and 75.2% of protein-coding genes had detectable expression (Figure S8H).

Read counts were generated for 19,913 protein-coding genes and CQN-normalized with correction of GC content bias (R package: cqn; function: cqn) [57]. The disease status effect size was estimated for 16,057 of these, with detectable expression in at least 15% of ALS samples (≥4 of 26) or 15% of CTL samples (≥2 of 12). CQN-normalized samples differing by sex and age at death overlapped within PC space (Figure S9A,B,D,E). ALS and CTL groups did not differ significantly with respect to the sex ratio (*p* > 0.05, Figure S9C), although age at death was significantly lower among ALS patients (*p* = 0.012, Figure S9F). Effect size estimates were generated from linear mixed-effects models (R package: lme4; function: lmer), in which the subject was treated as a random effect and independent variables included the disease status (ALS vs. CTL), sex (categorical) and age at death (continuous). Coefficient estimates were singular for 162 of the 16,057 models (1.4%). In these cases, expression was averaged by subject, and a fixed-effects linear model was fit using the same variables excluding the subject (R package: stats; function: lm).

### 2.7. Dataset F (GSE137810)

The New York Genome Center (NYGC) dataset includes bulk RNA-seq samples from post-mortem brain and spinal cord tissues of ALS patients (sporadic and familial) and normal CTL subjects [16,25,73,75,76,94]. The current study evaluated RNA-seq data released as of May 2025, including 3064 sequencing runs assigned separate SRR identifiers within the Sequence Read Archive (bioproject PRJNA573105) [34]. Most runs (2646 of 3064) had also been assigned sample identifiers (GSM prefix) within GEO under series accession GSE137810. Paired-end reads (100–125 bp) were generated using the Illumina NovaSeq 6000 (GPL24676) or Illumina HiSeq 2500 (GPL16791) platform (Illumina, San Diego, CA, USA). Samples were obtained from 10 different clinical sites as part of two projects (ALS consortium and Target ALS) and processed in multiple batches. The 3064 runs were annotated by merging information from multiple sources, including GEO series matrix files (GSE137810-GPL16791 series matrix.txt and GSE137810-GPL24676_series_matrix.txt), PRJNA573105 bioproject metadata, published supplemental data [26], and annotation files provided by NYGC ALS Consortium upon request (April 2025).

The 3064 runs were filtered to include 1530 from the cortex (frontal, motor, occipital or temporal). These were further filtered to include 1288 runs generated from samples with an RNA integrity number (RIN) greater than or equal to 5. Three runs linked to samples with sexual mosaicism were excluded. Of the remaining 1285 runs, 1104 were generated from an ALS patient or neurologically normal control subject. Seven of these were excluded because age at death was unknown. An additional 26 runs were excluded because the clinical phenotype was ambiguous, with conflicting annotation among data fields. Of the remaining 1071 runs, some appeared to be duplicate submissions with separate SRR identifiers. These were identified as runs where the number of sequenced bases was exactly the same (also same sample RIN value). There were 913 runs remaining after excluding duplicate submissions. Two of these were removed because they were linked to a sample with ambiguous annotation (CGND-HRA-00553).

The remaining 911 runs were associated with 812 unique sample identifiers (CGND number assigned by the NYGC ALS Consortium). The excess number of runs was due to multiple sets of reads from the same sample (i.e., technical replication). Raw fastq files from the 911 runs were obtained from SRA and mapped to the GRCh38/hg38 genome (as described for dataset *D* above). Mapped reads were summed across technical replicates linked to the same sample. This yielded read counts for 812 unique samples, of which 675 were derived from 312 ALS patients and 137 were from 74 CTL subjects. There was a median of 42.2 million reads/sample before read filtering (Figure S10A) and 41.4 million reads/sample after filtering (Figure S10B). The median number of mapped reads was 94.3% (Figure S10C) and 92.8% of reads mapped uniquely (Figure S10D). Nearly all reads (median 97.9%) (Figure S10E) were assigned to intragenic regions with most of these assigned to exons (median 58.6%) (Figure S10F). The median percentage of reads assigned to the ribosomal sequence was 1.4% (Figure S10G). The median percentage of protein-coding genes with detectable expression was 74.8% (Figure S10H).

Read counts from the 812 samples were CQN-normalized with correction for GC content bias (R package: cqn; function: cqn) [57]. These samples were associated with biological and technical sources of variation (Figure S11). Biological variables included the disease status, sex, age and cortex region. Technical variables included the platform, project (ALS Consortium vs. Target ALS), batch, site, RIN and primer type (random vs. non-random). Based on PC mapping, sample variation was not strongly associated with sex or age at death (Figure S12A–D), although samples from the temporal cortex did not overlap with those from other cortical regions within PC space (Figure S12E,F). Variation among samples, however, was more strongly associated with each of the above-mentioned technical variables (Figure S13A–L).

Hierarchical clustering was used to divide samples into groups, and Random Forest was used to predict groups based upon biological and technical variables [95]. Variable importance scores [96] from this procedure were highest with respect to sex, site, batch and project (Figure S14A). Technical factors were most strongly associated with intrasample variation based on goodness-of-fit testing applied to alternative linear mixed-effects models (Figure S14B). Further goodness-of-fit testing indicated that a base model (disease status, subject, age, sex, region) augmented by two technical variables (batch and RIN) provided a good balance between minimization of model deviance and model simplicity (Figure S14C).

The effect size associated with disease status was estimated for 15,807 protein-coding genes, with detectable expression in at least 15% of ALS samples (≥47 of 312) or 15% of CTL samples (≥21 of 137). Based on the above-mentioned analysis (Figure S14C), a linear mixed-effects model was formulated with subject (random effect) and six biological/technical variables as fixed effects (disease status, sex, age at death, cortical region, batch, RIN) (R package: lme4; function: lmer). This model resulted in singular model estimates for 14 of 15,807 genes (<1%). In these cases, a two-stage procedure was utilized to circumvent the need for mixed-effects modeling [66]. In stage 1, variation associated with batch and tissue was removed by obtaining residuals from a linear model with these factors as fixed effects. In stage 2, residuals were averaged by subject and used as the response variable in a linear model with the disease status, age, sex and average RIN (by subject) as fixed effects (R package: stats; function: lm).

### 2.8. Dataset G (EGAS00001004286)

Bulk RNA-seq was used to evaluate expression in motor cortexes from 11 sporadic ALS patients and 8 CTL subjects [52]. Paired-end reads (101 bp) were generated using the Illumina HiSeq 2500 platform. Raw fastq files were downloaded from the European Genome-phenome Archive (EGAS00001004286) [36]. An average of 119.1 million reads per sample were available prior to filtering (Figure S15A), with 115.3 million reads per sample remaining after filtering (Figure S15B). Quality-filtered reads were mapped to the GRCh38/hg38 genome as described above (see dataset *D*). An average of 95% of reads were successfully mapped (Figure S15C), with 90.2% of reads mapped uniquely (Figure S15D). The average percentage of reads mapped to intragenic and exonic regions was 97.7% and 58.3%, respectively (Figure S15E,F). Approximately 0.3% of reads on average were assigned to ribosomal sequences (Figure S15G). Expression was quantified for 19,837 protein-coding genes, and 73.8% of these on average had detectable expression (Figure S15H). Read counts were CQN-normalized with correction for GC content bias (R package: cqn; function: cqn) [57]. The effect size associated with disease status was estimated for 15,302 protein-coding genes, with detectable expression in at least 15% of ALS samples (≥2 of 11) or 15% of CTL samples (≥2 of 8). A fixed-effects linear model was used with the disease status as the primary independent variable and age (continuous) and sex (categorical) as covariates (R package: stats; function: lm).

### 2.9. Dataset H (GSE174332/PRJNA1073234)

Gene expression was evaluated using snRNA-seq in 66 samples from 33 ALS patients (sporadic, *C9ORF72*) and 32 samples from 16 CTL subjects [15]. Samples were obtained from the motor or prefrontal cortex. Fastq files containing paired-end reads (103–200 bp) were downloaded from the Sequence Read Archive (PRJNA1073234) [34]. Among all samples, median values were 259.2 million for reads/sample (Figure S16A), 9333.5 for cells/sample (Figure S16B) and 30,400 for reads/cell (Figure S16C). Other sequencing quality control parameters were within an acceptable range (Figure S16D–F). Reads were mapped to the GRCh38/hg38 genome using the 10× Genomics cellranger count pipeline. The distribution of read and feature counts among cells was similar among samples (Figure S17A,B). Likewise, samples were similar with respect to the distribution of mitochondrial and ribosomal gene percentages among cells (Figure S17C,D). The median percentage of mapped reads was 95.3% (Figure S16G).

Raw gene counts were summed across cells to generate pseudobulk samples (R package: Seurat, function: AggregateExpression) [97]. Pseudobulk counts were generated for 18,689 protein-coding genes, and the median percentage of these with detectable expression among samples was 73.5% (Figure S16H). A gene was considered to have detectable expression in a given sample if the count was non-zero with FPKM > 0.05. Pseudobulk read counts for each sample were CQN-normalized with correction for GC content bias (R package: cqn; function: cqn) [57]. Samples associated with males and females overlapped in two-dimensional PC space (Figure S18A,B), and the percentage of males was similar among ALS and CTL subjects (Figure S18C). Samples from younger and older patients were more separated in PC space (Figure S18D,E), and the age at death was significantly lower among ALS compared to CTL subjects (*p* < 0.01; Figure S18F). Samples from different cortical regions (motor vs. prefrontal) were overlapping in PC space (Figure S18G,H), and there was an equal fraction of samples from each region among ALS and CTL subject samples (Figure S18I). The effect size associated with the disease status was estimated for 14,678 protein-coding genes, with detectable expression in at least 15% of ALS samples (≥10 of 66) or at least 15% of CTL samples (≥5 of 32). This analysis was performed using linear mixed-effects models with the subject as a random effect and the disease status, brain region, sex and age as fixed effects (R package: lme4, function: lmer). This generated singular coefficients for 198 of the 14,678 genes tested. In these cases, the two-stage procedure described above was used (dataset *F*), wherein data was averaged by subject, estimated effects of the brain region were removed by regression, and a fixed-effects linear model was formulated using residuals as the response variable (R package: stats; function: lm).

### 2.10. Dataset I (syn45351388)

Gene expression was measured using snRNA-seq in 36 samples from 16 familial ALS patients (*C9ORF72*) and 26 samples from 12 CTL subjects [53] (some samples were excluded, see below). Samples were obtained from the frontal or occipital cortex, and read sequencing was performed using the Illumina NovaSeq 6000 platform. Pair-end reads divided into 998 fastq file pairs were obtained from the Synapse database (syn45351388) and mapped using the cellranger count pipeline as described above (dataset *H*). Pseudobulks were generated by summing gene counts across cells within a sample (R package: Seurat, function: AggregateExpression), and results from paired fastq files linked to the same sample were summed together. The median numbers of reads and cells per sample were 512.2 million and 42,531, respectively, with a median of 11,700 reads per cell (Figure S19A–C). The median sequencing saturation was 21.5% (Figure S19E), and 93.4% of bases had quality scores greater than 30 (Figure S19F). The median percentage of mapped reads was 96.3% (Figure S19G).

Pseudobulk read counts were generated for 18,689 protein-coding genes, of which the median percentage with detectable expression among samples was 73% (Figure S19H). Read and feature counts were similar among samples (Figure S20A,B). There were two samples for which cells had a relatively high percentage of mitochondrial reads (CTL-72M-OCX-1 and CTL-71F-OCX-1; Figure S20C). These samples were excluded from differential expression analyses (see below). The ribosomal read percentage distribution was similar among samples although tended to be slightly higher among ALS compared to CTL samples (Figure S20D). Samples associated with different sexes, ages or cortical regions overlapped moderately in two-dimensional PC space (Figure S21A,B,D,E,G,H). Age at death was significantly lower among ALS patients (*p* < 0.01; Figure S21F), but there was an equal number of males and females in each disease status group (Figure S21C). Likewise, ALS and CTL samples were balanced with respect to the percentage of samples from each cortical region (Figure S21I).

Pseudobulk read counts were CQN-normalized with GC content bias correction (R package: cqn; function: cqn) [57]. The effect size associated with the disease status was estimated for 14,721 protein-coding genes, with detectable expression in 15% of ALS samples (≥6 of 36) or 15% of CTL samples (≥4 of 24). This was achieved using linear mixed-effects models (R package: lme4, function: lmer) with the subject as a random effect, disease status as the primary independent variable, and multiple covariates, including the cortex region (categorical), sex (categorical) and age (continuous). For 2086 of 14,721 genes evaluated (14.2%), mixed-effects models generated singular coefficient estimates. In these cases, the two-stage procedure described above (dataset *F*) was applied with data averaged by subject, effects of the cortex region removed by regression, and adjusted expression values (residuals) treated as the response variable of a linear model with the disease status, sex and age as fixed effects (R package: stats; function: lm).

### 2.11. Dataset J (PRJNA918304)

Gene expression was evaluated by snRNA-seq in orbitofrontal cortexes from 17 ALS patients (sporadic, C9ORF72) and 6 CTL subjects [54]. Sequencing was performed using the Illumina NovaSeq 6000 platform. Fastq files containing paired-end reads were obtained from the Sequence Read Archive (PRJNA918304). Reads were mapped to the GRCh38/hg38 genome using the cellranger count pipeline (dataset *H* above) and aggregated across cells within samples to generate pseudobulks (R package: Seurat; function: AggregateExpression). The median numbers of reads and cells per sample were 2.07 billion and 23,076, respectively (Figure S22A,B). The median number of reads per cell was 78,100 (Figure S22C). Median sequencing saturation was 67.3% (Figure S22D), with 93.6% of reads having a quality score greater than 30 (Figure S22E) and 89.6% of reads within cells (Figure S22F). The median percentage of mapped reads was 95.9% (Figure S22G).

Pseudobulk counts were generated for 18,689 protein-coding genes, and 75.2% of these (median) had detectable expression among samples (Figure S22H). Samples were similar with respect to the distribution of counts, features and percentage of reads mapped to mitochondrial genes (Figure S22K). The percentage of reads assigned to ribosomal genes was slightly higher in ALS compared to CTL samples (Figure S22L). Pseudobulk read counts were CQN-normalized with correction for GC content bias (R package: cqn; function: cqn) [57]. Male and female samples largely overlapped within two-dimensional PC space (Figure S23A,B), but there was less overlap among samples from younger versus older subjects (Figure S23D,E). ALS and CTL subjects did not differ significantly with respect to sex or age at death (*p* > 0.05; Figure S23C,F). The effect size associated with disease status was estimated for 15,197 protein-coding genes, with detectable expression in at least 15% of ALS samples (≥3 of 17) or 15% of CTL samples (≥1 of 6). Effect size estimates were generated using linear models with the disease status as the primary independent variable and sex (categorical) and age at death (continuous) as covariates (R package: stats; function: lm).

### 2.12. Dataset K (GSE219280)

Gene expression was evaluated by snRNA-seq in the motor and frontal cortexes of 12 samples from 6 ALS patients (*C9ORF72*) and 12 samples from 6 CTL patients [20]. Paired-end reads were generated using the Illumina NovaSeq 6000 platform. Raw fastq files were downloaded from the Sequence Read Archive (PRJNA907951). The median number of reads per sample was 310.8 million (Figure S24A), with 25,430 cells per sample (Figure S24B) and 12,700 reads/cell (Figure S24C). The median sequencing saturation was 19.7% (Figure S24D), and 93.4% of bases had quality scores greater than 30 (Figure S24E). A median of 74.8% of reads were within cells (Figure S24F), and 96.1% of reads were mapped to the GRCh38/hg38 genome (Figure S24G). Gene counts were aggregated across cells to generate pseudobulk samples (R package: Seurat, function: AggregateExpression). Counts were generated for 18,689 protein-coding genes, and a median of 74.3% of these genes had detectable expression (Figure S24H). Read and feature count distributions were similar among samples (Figure S24I,J). One sample had an increased percentage of mitochondrial reads (ALS-59F-A4-MCX, GSM6781913), but samples were otherwise similar in this respect (Figure S24K). The percentage of ribosomal reads was higher among ALS samples (Figure S24F). Pseudobulk read counts were CQN-normalized with correction for GC content bias (R package: cqn; function: cqn) [57]. Samples associated with different sexes, ages at death and cortical regions were overlapping within two-dimensional PC space (Figure S25A,B,D,E,G,H). One sample was an outlier within PC space (CTL-73F-C5-FCX, GSM6781936) but otherwise unremarkable in quality control metrics and thus retained. Samples associated with the ALS and CTL groups were balanced with respect to the sex, age at death and percentage of samples from each anatomical region (Figure S25C,F,I).

Disease status effect size estimates were generated for 14,798 genes with detectable expression in 15% (≥2 of 12) of ALS samples or 15% (≥2 of 12) CTL samples. A linear mixed-effects model was used with the subject as a random effect and all other variables as fixed effects (disease status, cortex region, sex, age) (R package: lme4, function: lmer). This approach resulted in 1926 singular models (13.0%). In such cases, the two-stage procedure described above (dataset *F*) was used, in which expression values were averaged by subject, then adjusted by regression to remove the effect of the cortex region, and finally, adjusted expression values (residuals) were used as the response variable in a fixed-effects model with the disease status (categorical), sex (categorical) and age at death (continuous) as independent variables (R package: stats; function: lm).

### 2.13. Dataset L (KCL BrainBank)

Bulk RNA-seq was used to evaluate gene expression in post-mortem motor cortexes from 112 sporadic ALS patients and 59 CTL subjects [29,98,99]. Paired-end reads (150 bp) were sequenced using the Illumina HiSeq 4000 platform (Illumina, San Diego, CA, USA). Raw reads were mapped to the GRCh38/hg38 genome as described above (dataset *D*). Read counts were CQN-normalized with correction for GC content bias (R package: cqn; function: cqn) [57]. Samples with lower and higher RIN values mapped to different regions within two-dimensional PC space (Figure S26A,B). The average RIN was significantly higher among ALS samples compared to CTL samples (*p* < 0.001; two-tailed two-sample *t*-test; Figure S26C). Samples were filtered to include only 136 samples with RIN ≥ 5 (98 ALS samples, 38 CTL samples). This mitigated the difference between ALS and CTL samples with respect to the average RIN, although there remained a significant group difference (*p* < 0.01; Figure S26D). Raw gene counts were available for 19,377 protein-coding genes. Of these, effect size estimates associated with the disease status were obtained for 11,771 genes with detectable expression in at least 15% of ALS samples (≥15 of 98) or 15% of CTL samples (≥6 of 38). Effect size estimates were generated using a linear fixed-effects model with the disease status (categorical) and RIN (continuous) as independent variables (R package: stats; function: lm).

### 2.14. Dataset M (EGAS00001007318)

Bulk RNA-seq was used to evaluate gene expression in post-mortem prefrontal cortexes from 51 sporadic ALS patients and 50 CTL subjects [9,23]. The analysis considered a subset of 66 samples with RIN ≥ 5 (38 ALS, 28 CTL). Single-end reads had been generated using the Illumina NovaSeq 6000 platform. Raw reads were mapped to the GRCh38/hg38 genome, as described previously [29]. There were averages of 34 million reads per sample prior to quality control read filtering (Figure S27A) and 29.2 million reads per sample after filtering (Figure S27B). Greater than 90% of reads were mapped to the GRCh38/hg38 genome in all samples (Figure S27C), with >80% of reads mapped uniquely (Figure S27D). An average of 96.8% of reads were mapped to intragenic regions (Figure S27E), with 60.2% of reads mapped to exons (Figure S27F). The percentage of ribosomal reads was <5% in all samples (Figure S27G), and an average of 73.6% of protein-coding genes had detectable expression (Figure S27H). The number of mapped reads was counted for each gene and CQN-normalized with correction for GC content bias (R package: cqn; function: cqn) [57].

The disease status effect size was estimated for 15,491 protein-coding genes with detectable expression in at least 15% of ALS samples (≥6 of 38 samples) or 15% of CTL samples (≥5 of 28 samples). Samples from males and females overlapped in two-dimensional PC space (Figure S28A,B), although the frequency of males was significantly higher among ALS samples (*p* = 0.047; Figure S28C). Samples from young and older subjects mapped to different regions in PC space (Figure S28D,E), and the age at death was significantly lower among ALS patients (*p* = 0.012; Figure S28F). Samples with differing RIN values also mapped to different regions in PC space (Figure S28G,H), although the average RIN did not differ significantly between ALS and CTL samples (Figure S28I). Effect sizes were estimated using fixed-effects linear models (R package: stats; R function: lm). Linear models included the disease status as an independent variable with sex (categorical), age at death (continuous) and RIN (continuous) as covariates.

### 2.15. Dataset N (GSE271156)

Gene expression was profiled using snRNA-seq in motor cortexes from 4 sporadic ALS patients and 2 CTL subjects [26]. Paired reads were generated using the NextSeq 2000 platform. Among the 6 samples, mean values were 151.3 million for reads/sample (Figure S29A), 4472 for cells/sample (Figure S29B) and 36,900 for reads/cell (Figure S29C). All other quality control parameters were within an acceptable range (Figure S29D–L). Gene counts were aggregated across cells to generate pseudobulk samples (R package: Seurat, function: AggregateExpression). Pseudobulk gene counts were generated for 18,689 protein-coding genes and CQN-normalized with correction for GC content bias (R package: cqn; function: cqn) [57]. The effect size associated with the disease status was estimated for 15,339 protein-coding genes with detectable expression in at least 15% of ALS samples (≥1 of 4) or at least 15% of CTL samples (≥1 of 2 samples). Effect sizes were calculated using linear models with the disease status (categorical) and sex (categorical) as fixed effects (R package: stats; function: lm).

### 2.16. Dataset O (NG00178)

Bulk RNA-seq was used to evaluate gene expression in multiple post-mortem cortical regions from ALS and CTL subjects (prefrontal cortex, motor cortex, occipital cortex) [14]. Raw fastq files were obtained from the National Institute on Aging Genetics of Alzheimer’s Disease Data Storage Site (NIAGADS) under accession NG00178. The current analysis evaluated all 99 cortical samples available, which included 66 ALS samples (22 individuals) and 33 CTL samples (11 individuals). Samples were sequenced using the Illumina NovaSeq 6000 platform (151 bp paired-end reads). Among the 99 samples, a median of 26.4 million reads were available prior to read quality filtering (Figure S30A), of which 25.1 million reads per sample remained after quality filtering (Figure S30B). The median percentage of mapped reads, uniquely mapped reads, intragenic reads and exonic reads exceeded 89%, respectively (Figure S30C–F). The median percentage of ribosomal reads was less than 0.01%, and the median percentage of protein-coding genes with detectable expression was 69.1% (Figure S30H).

Samples were mapped to the GRCh38/hg38 genome, as described above (dataset *D*). Normalized expression values were calculated from mapped read counts using CQN normalization with correction for GC content bias (R package: cqn; function: cqn) [57]. When samples were plotted with respect to two-dimensional principal component space, there was overlap with respect to samples varying by sex and age at death (Figure S31A,B,D,E), but samples from different cortical regions were separated (Figure S31G,H). The proportion of males was similar between ALS and CTL subjects (Figure S31C) whereas age at death was significantly lower among ALS subjects (Figure S31F). Disease status (ALS vs. CTL) effect size estimates were obtained for 14,601 protein-coding genes with detectable expression in at least 15% of ALS samples (≥10 of 66) or 15% of CTL samples (≥5 of 33). The estimated effect size for each gene was obtained using a linear mixed-effects model (R package: lme4, function: lmer), with the subject as a random effect (categorical) and other available variables as categorical (disease status, sex, brain cortical region) or continuous (age at death) fixed effects.

### 2.17. Dataset P (JGAS000852)

Gene expression was evaluated using snRNA-seq in post-mortem cortexes from 6 sporadic ALS patients and 6 CTL subjects [55]. Paired-end 150 bp reads were generated using the NovaSeq 6000 platform. Raw data were submitted to the DNA Data Bank of Japan (accession JGAS000852), and processed files were deposited in figshare [100]. Mapped read counts were obtained from a Seurat object stored in R Data Serialization (RDS) format (“brain_som.rds”). Samples were similar with respect to their distribution of read counts, feature counts and percentage of ribosomal reads (Figure S32A,B,D). There was one sample (CTL-83M-6; subject ID: HC_8; patient ID: B22) for which nuclei had a relatively increased percentage of mitochondrial reads (Figure S32C). Pseudobulk samples were generated by pooling mapped counts across nuclei (R package: Seurat; function: AggregateExpression). Aggregate counts for each sample were CQN-normalized with correction for GC content bias (R package: cqn; function: cqn) [57]. Samples associated with different sexes or ages at death were overlapping within two-dimensional PC space (Figure S33A,B,D,E). There was no significant difference between ALS and CTL groups with respect to sex distribution or average age at death (*p* > 0.05; Figure S33C,F). Pseudobulk counts were available for 18,689 protein-coding genes. The treatment effect (ALS vs. CTL) was estimated for 14,970 of these expressed in 15% of ALS samples (≥1 of 6 samples) or 15% of CTL samples (≥1 of 6 samples). The one sample with a higher percentage of mitochondrial reads (CTL-83M-6) was retained in these analyses since none of the normalized expression values for any gene met the outlier criteria (i.e., Z-score ≥ 3 or ≤ −3). Treatment effects were estimated using fixed-effects linear models with the disease status (categorical) as the primary independent variable and with the age at death (continuous) and sex (categorical) as covariates (R package: stats; function: lm).

### 2.18. Alternative Normalization

Datasets from this study were normalized using methods that incorporate GC content bias correction (exceptions: microarray datasets *A* and *C*). For RNA-seq datasets (*D*–*P*), we utilized conditional quantile normalization (CQN) [57]. For one microarray dataset (*B*), normalization was performed using single-channel array normalization (SCAN) [56]. For comparison purposes, read counts from RNA-seq datasets (*D*–*P*) were also normalized using the variance-stabilizing transformation (VST) without correction for GC content bias (R package: DESeq2; function: vst) [101]. Additionally, dataset B was alternatively normalized using the robust multichip average algorithm (RMA) without correction for GC content bias (R package: oligo; function: rma) [83]. Some results from these alternative normalization approaches are presented. However, unless otherwise indicated, the results presented and discussed below were obtained from data normalized by CQN (datasets *D*–*P*) or SCAN (dataset *B*). No alternative normalization was performed for datasets *A* and *C*.

### 2.19. Meta-Analysis

Differential expression effect sizes (ALS vs. CTL) were calculated by gene and for each study based on the unstandardized coefficient estimate associated with the disease status in linear models. Coefficient estimates were converted to a covariate-adjusted standardized mean difference (SMD) [102] utilizing the unstandardized coefficient estimate, standard deviation of expression values, ALS treatment sample size, and CTL treatment sample size (R package: esc; function: esc_B) [103]. In each case, ALS and CTL group sample sizes were set equal to the number of unique individuals in each group, rather than the total number of samples, to avoid assigning excess weight to studies with multiple samples from the same individuals.

An effect size summary estimate for each gene was calculated using inverse variance random effects meta-analysis (R package: meta, function; metagen) [104]. This analysis was performed using 16,579 protein-coding genes for which at least 4 SMD estimates were available from separate studies. The majority of these genes (87.7%) had SMD estimates available from 10 or more studies. Raw *p*-values and confidence intervals associated with pooled SMD estimates were generated from the standard normal distribution. To control the false discovery rate (FDR), raw *p*-values were adjusted for multiple tests among the 16,579 genes utilizing the Benjamini–Hochberg method [105]. Meta-analysis-supported differentially expressed genes (DEGs) were defined as those with an FDR < 0.05, with at least a “small” treatment effect based on a classical interpretation of the SMD summary measure (i.e., meta-SMD estimate ≥ 0.20 or ≤−0.20) [106].

Moderators associated with effect size heterogeneity were analyzed using a mixed-effects meta-regression model (R package: metafor; function: rma) [107]. The importance of moderating variables was only evaluated with respect to genes with significant effect size heterogeneity based on Cochran’s Q statistic (FDR < 0.05) [108]. Additionally, these analyses were only performed for genes with an SMD estimate available from each of the 16 included studies. Multiple mixed-effects meta-regression models were formulated (Equations (1)–(7) below).

*Y*_i_ = *β*_0_ + *β*_1_*x*_1i_ + *β*_2_*x*_2i_ + *μ*_i_ + *ε*_i_ (1)

*Y*_i_ = *β*_0_ + *β*_3_*x*_3i_ + *μ*_i_ + *ε*_i_ (2)

*Y*_i_ = *β*_0_ + *β*_4_*x*_4i_ + *μ*_i_ + *ε*_i_ (3)

*Y*_i_ = *β*_0_ + *β*_5_*x*_5i_ + *μ*_i_ + *ε*_i_ (4)

*Y*_i_ = *β*_0_ + *β*_6_*x*_6i_ + *μ*_i_ + *ε*_i_ (5)

*Y*_i_ = *β*_0_ + *β*_7_*x*_7i_ + *μ*_i_ + *ε*_i_ (6)

Equations (1)–(6) above represent single-moderator models where *Y*_i_ represents study-specific effect sizes (SMD estimates), *μ*_i_ represents the true (unknown) effect sizes (i.e., between-study variability), and *ε*_i_ represents sampling variation (i.e., within-study variability). Moderator variables include expression profiling technology, coded as two 0–1 indicator variables (*x*_1_ and *x*_2_), together representing a three-level categorical variable designating whether study data had been generated using microarray, bulk RNA-seq or snRNA-seq. Additional moderator variables include the percentage of ALS patient samples obtained from the motor cortex (*x*_3_), the average post-mortem interval among ALS and CTL samples in hours (*x*_4_), the average age of ALS patients (*x*_5_), the percentage of ALS patient samples from males (*x*_6_), and the percentage of ALS samples from patients with sporadic disease (*x*_7_).

*Y*_i_ = *β*_0_ + *β*_1_*x*_1i_ + *β*_2_*x*_2i_ + *β*_3_*x*_3i_ + *β*_4_*x*_4i_ + *μ*_i_ + *ε*_i_ (7)

Whereas Equations (1)–(6) represent single-moderator models, Equation (7) incorporates 3 of the 6 moderator variables, chosen from those mentioned above. This is referred to as the “full model” and permits effects of any one moderator to be evaluated while controlling for those of the other two. Models were compared based on multiple metrics. An omnibus test of moderators (Q_M_ test) was performed to assess the null hypothesis that one or multiple moderator coefficients (β) are equal to zero [107]. A test for residual heterogeneity (Q_E_ test) was also performed to assess whether effect size variation not accounted for by a model exceeds that expected on the basis of sampling variation alone [107]. The *I*^2^ and *H*^2^ statistics were calculated for each gene to estimate the percentage of residual effect size heterogeneity (between-study variability) not accounted for by the meta-regression model [109].

### 2.20. Meta-Analysis DEGs and Enrichment of Gene Annotations

Meta-analysis DEGs were assessed for gene annotation enrichment relative to other non-DEG protein-coding genes included in the meta-analysis. A conditional hypergeometric test (R package: GOstats, function: hyperGTest) [110] was used to assess for enrichment of Gene Ontology terms, including those associated with the biological process (BP), cell component (CC) and molecular function (MF) ontologies [111]. Fisher’s exact test was used to assess for enrichment with respect to gene annotations from multiple other databases, including the Kyoto Encyclopedia of Genes and Genomes (KEGG) [112], WikiPathways [113], Pathway Commons [114], Medical Subject Headings (MeSH) [115], Drug signatures database (DSigDB) [116] and Molecular Signatures Database (MSigDB) [117].

### 2.21. Transcription Factor (TF) Binding Site Analysis

DEGs were evaluated to identify transcription factor binding sequences with increased proximity to DEG transcription start sites (TSSs). The analysis was performed using the CORE vertebrate collection of 1019 position weight matrix (PWM) models from the JASPAR database [118]. DNA sequences matching each PWM model in the GRCh38/hg38 genome sequence were identified as described previously [67]. Motifs without at least one match on each human chromosome were excluded, leaving a final set of 970 motifs used in further analyses. The nearest neighbor distance was then calculated between the TSS for each gene and each PWM match on the same chromosome [67]. For each PWM model, the NN distance was then compared between DEGs and non-DEGs (Wilcoxon rank sum test) [67]. To control the false discovery rate among the 970 PWM models, raw *p*-values were adjusted using the Benjamini–Hochberg method [105].

### 2.22. Comparison to GWAS Findings, Cell Type Markers, snRNA-seq Clusters and Region-Specific Expression Markers

The NHGRI-EBI GWAS catalog [60] was used to identify genes overlapping or near loci significantly associated with ALS-related traits. The analysis utilized catalog entries linked to the following 7 ALS-related disease traits: ALS, ALS (age of onset), ALS (*c9orf72* mutation interaction), ALS in *c9orf72* mutation negative individuals, ALS in *c9orf72* mutation positive individuals, ALS (sporadic), ALS or frontotemporal dementia and rapid functional decline in sporadic ALS. This identified 367 unique genes overlapping or near loci associated with ALS-related traits, of which 238 were detectable in the cortex and included in the differential expression meta-analysis.

To detect trends among genes with cell type-specific expression, marker genes were identified from multiple databases, including Brain Cell Type Specific Gene Expression Analysis (Bretigea) [61], CellMarker 2.0 [62] and Cellmarker accordion [63]. Marker genes linked to cell clusters defined using an unsupervised snRNA-seq approach were identified from two prior post-mortem brain studies [64,65]. The first study [64] identified superclusters by applying snRNA-seq to 3.37 million cells from 10 post-mortem brain regions (without known neurological disease). Based on supplemental data from this study, we identified 880 marker genes uniquely associated with 30 superclusters, of which 539 were expressed by the cortex and had been included in the differential expression meta-analysis. The second study [65] used snRNA-seq to identify subclasses based on 1.12 million cells from the post-mortem cerebral cortex (without known neurological disease). Using supplemental data from this study, we identified 3271 genes linked to 21 subclasses, of which 2924 had been included in the differential expression meta-analysis. This latter study had also used spatial transcriptomics to identify genes with region-specific expression in the human cortex. Based upon supplemental data files [65], we identified 2924 genes with expression localized to one or more cortical regions (of which 2638 were included in the meta-analysis). Additionally, we identified 1084 genes with expression localized to one or more laminar layers (of which 1068 had been included in the meta-analysis).

## 3. Results

### 3.1. Disease Status Effect Size Estimates

Samples from each dataset (Table 1) were plotted in two-dimensional PC space and color-coded by disease status (Figure S34). ALS and CTL samples were non-overlapping and, for each dataset, the disease status of samples could be distinguished by a linear discriminant function (balanced accuracy ≥ 56.1%, Figure S34). Disease status classification accuracy was greater than 80% for three datasets (datasets *A*, *B* and *J*; Figure S34). Overlap between ALS and CTL confidence ellipses was no greater than 77.3%, with less than 20% overlap in some cases (datasets *A*, *B* and *J*; Figure S35). The disease status is thus a prominent factor contributing to post-mortem cortex sample variation.

**Figure 1:**
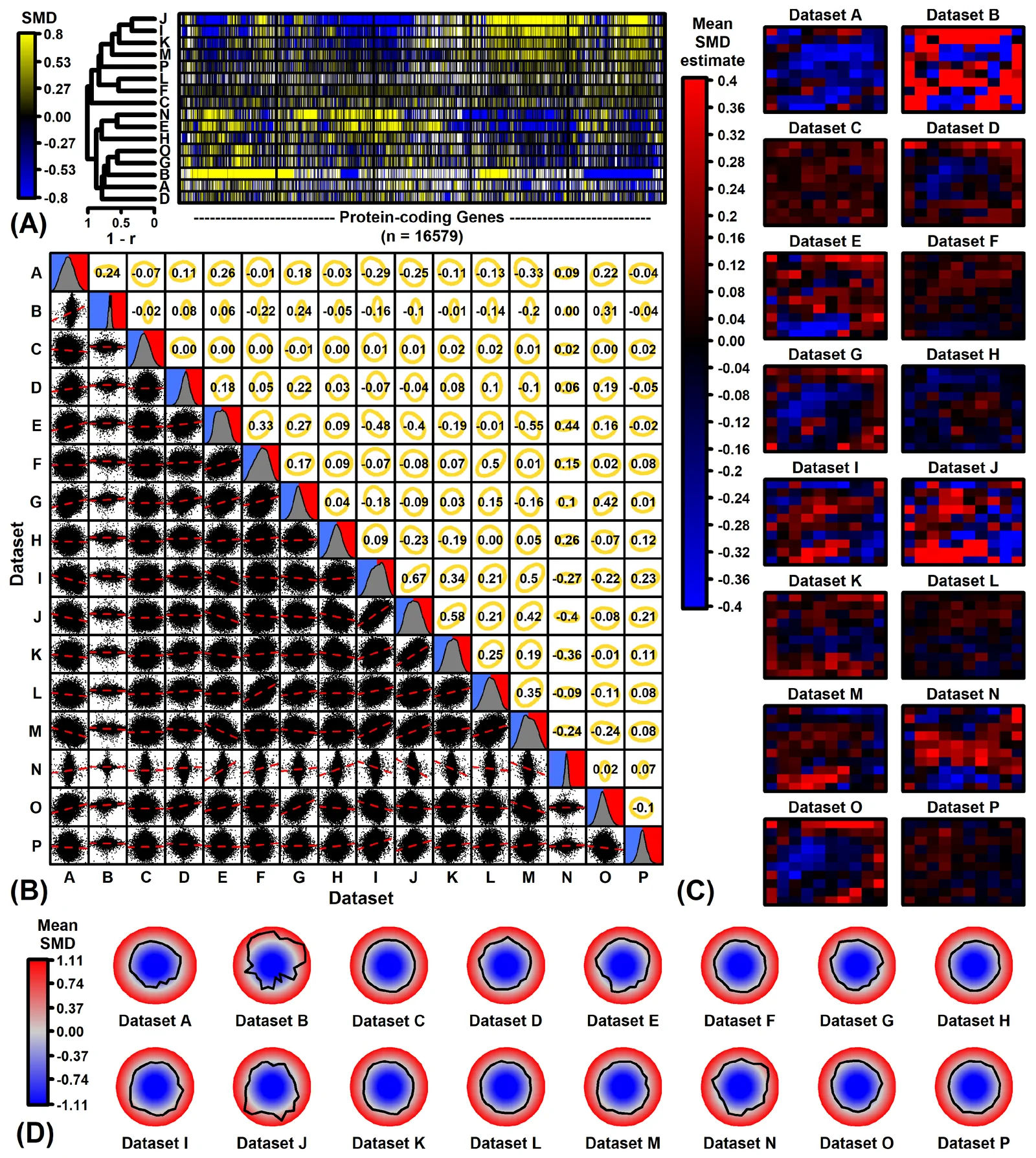
SMD estimate comparison. (**A**) Cluster analysis. Datasets (rows) were hierarchically clustered based upon the Spearman correlation coefficient between SMD estimates. Genes (columns) were clustered based upon the Euclidean distance. SMD estimates are color-coded (see legend), with whitespace used for missing values (i.e., genes missing from some datasets but present in others). (**B**) Correlation matrix. Below diagonal: SMD scatterplots with least square regression estimates (dashed red lines). Diagonal: SMD histograms (red: SMD > 0; blue: SMD < 0). Above diagonal: Spearman correlation coefficients with yellow ellipses outlining the middle 90% of datapoints (Mahalanobis distance). (**C**) Self-organizing maps (SOM). An SOM was generated based upon co-expression relationships determined from an independent RNA-seq dataset generated from a heterogenous collection of human tissues, cell types and cell lines (GSE138734). The SOM was then color-coded for each dataset based upon average SMD estimates of genes assigned to each SOM region (see legend). (**D**) Radial plots. Gene expression clusters were identified from an independent dataset (GSE138734). Radial plots show the average SMD estimate of genes within each cluster (see scale).

Datasets were clustered based on the disease status effect size (SMD) of protein-coding genes (Figure 1A). The dendrogram suggested two main groups, with one group (*J*, *I*, *K*, *M*, *P*, *L*, *F*, *C*) having a higher average PMI (20.2 ± 3.5 h) and the second group (*N*, *E*, *H*, *O*, *G*, *B*, *A*, *D*) having a lower average PMI (12.3 ± 2.6 h) (Figure 1A). Pairwise Spearman correlations between SMD estimates ranged from −0.55 to 0.67 (Figure 1B). Genes were then grouped based upon expression patterns from an independent reference dataset (GSE138734) generated from heterogeneous human tissue samples [119]. This revealed prominent directional SMD trends among genes assigned to the same cluster for four datasets (*B*, *E*, *J*, *N*), indicating a concordance of effect sizes among genes linked to the same pathway or cell type (Figure 1C,D).

*p*-values associated with effect size estimates were non-uniformly distributed for all datasets (*p* < 0.001; one-sample Kolmogorov–Smirnov test; Figure S36), with several having an L-shaped distribution (*A*, *F*, *H*, *I*, *J*, *L*, *M*, *O*), reflecting an increased abundance of low *p*-values (Figure S37). However, based on stringent criteria (FDR < 0.05 with SMD ≥ 0.20 or SMD ≤ −0.20), it was not possible to identify any significant effect size estimates with respect to 9 of 16 datasets (Figure S38). Some significant effect size estimates could be identified with respect to seven datasets (*C*, *F*, *H*, *J*, *L*, *M*, *O*), most of which had larger sample sizes (≥20–30 samples in both ALS and CTL groups) (Figure S38).

### 3.2. Sources of Effect Size and Differential Expression Bias

Sources of effect size and differential expression bias were investigated. For datasets with multiple samples per subject (datasets *E*, *F*, *H*, *I*, *K*, *O*), some effect size estimates had been generated from fixed-effects models after averaging by subject. This was carried out for a minority of genes for which the mixed-effects model generated a singular fit. For such datasets, the distribution of SMD estimates from fixed-effects models overlapped with those from mixed-effects models, although distributions differed significantly in two cases (i.e., datasets *I* and *K*; *p* < 0.001; Wilcoxon rank sum test; Figure S39A–F). In all cases, however, fixed-effects model estimates correlated similarly, or better, with those from other datasets, as compared to mixed-effects model SMD estimates (Figure S39G).

Low-expressed genes were biased towards ALS-decreased expression for five datasets (*F*, *H*, *L*, *M*, *O*), whereas high-expressed genes were biased towards ALS-increased expression for five datasets (*C*, *I*, *K*, *M*, *O*) (Figure S40). These patterns were mostly limited to genes near the extremes of the expression distribution (extremely low or high expression) and were thus not indicative of systemic trends (Figure S40). Short genes demonstrated mild bias towards ALS-increased expression with respect to two datasets (*F*, *K*; Figure S41). There were also four datasets for which high-GC-content genes had a slightly increased proportion of ALS-increased DEGs (*F*, *G*, *H*, *O*; Figure S42). Overall, however, these biases were improved considerably compared to those obtained when GC content correction had not been implemented during the normalization step, i.e., using RMA-normalized microarray data [83] or VST-normalized RNA-seq data [101]. With these normalization approaches, multiple datasets exhibited severe differential expression biases related to the gene length (*D*, *H*, *I*, *J*, *K*, *O*, *P*; Figure S43) and/or GC content (*B*, *F*, *G*, *H*, *J*, *L*, *M*; Figure S44). Normalization of individual datasets with correction for GC content bias [56,57] thus ameliorated differential expression bias related to both gene length and GC content.

### 3.3. Effect Size Heterogeneity and Moderator Variables

**Figure 2:**
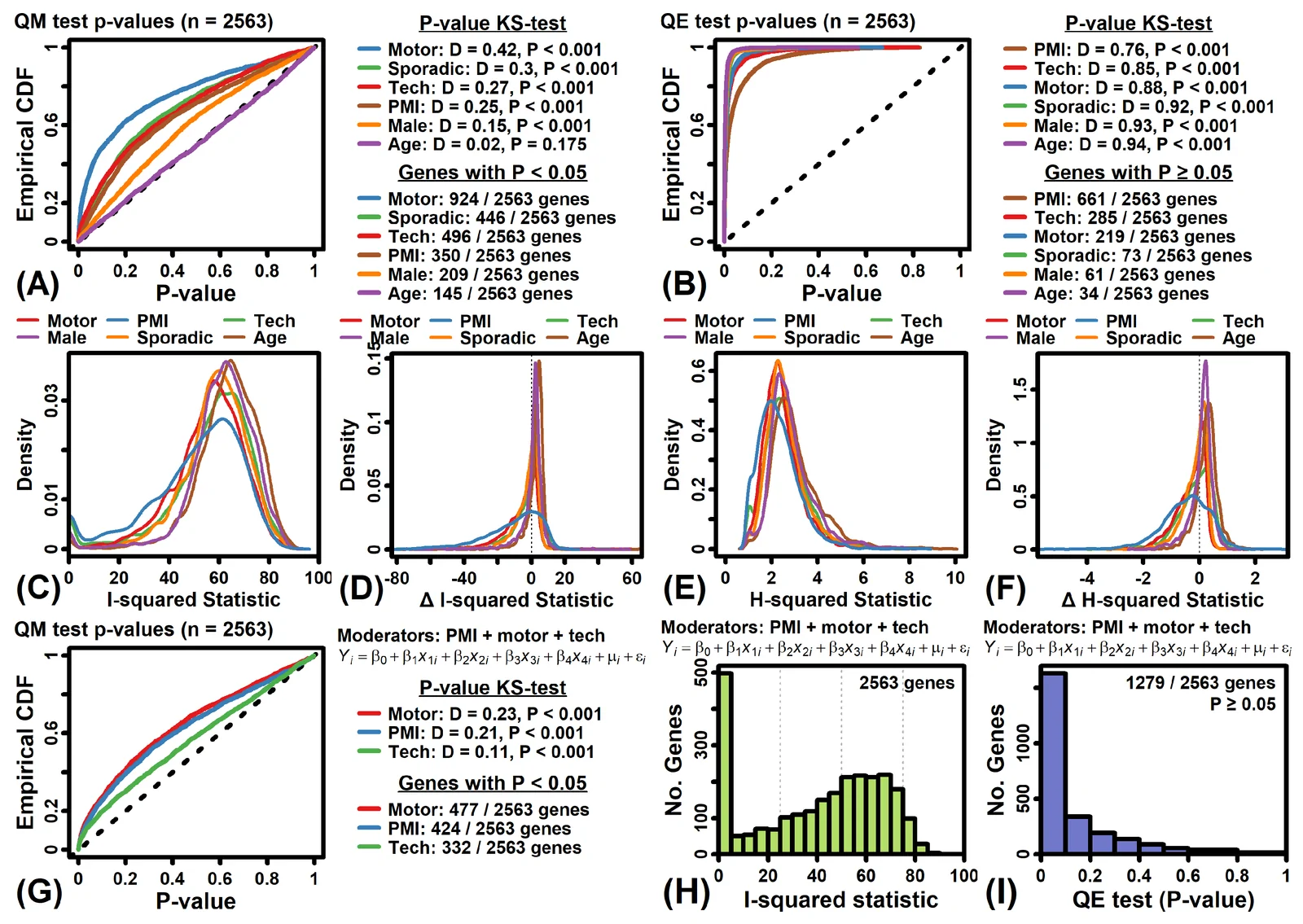
Meta-regression result summary. Analyses were performed for 2772 genes with significant effect size heterogeneity (FDR < 0.05, Cochran’s Q). (**A**) *p*-values associated with the omnibus test of moderators. (**B**) *p*-values from the test for residual heterogeneity. In (**A**,**B**), empirical cumulative *p*-value distributions (ECDFs) are shown for six single-moderator models. A one-sample Kolmogorov–Smirnov (KS) test was performed to test for deviation from a uniform *p*-value distribution (dashed line). (**C**) *I*^2^ statistic distributions. (**D**) Δ *I*^2^ statistic distributions. (**E**) *H*^2^ statistic distributions. (**F**) Δ *H*^2^ statistic distributions. In (**E**,**F**), the change in each statistic is shown relative to a base model without moderator variables (i.e., single-moderator model—base model without moderators). (**G**) *p*-values associated with the omnibus test of moderators (full model with three moderating variables). The test was performed for each moderator variable, and the KS-test was used to test for any significant deviation from a uniform *p*-value distribution. (**H**) *I*^2^ statistic distribution (full model). (**I**) *p*-values associated with the test for residual heterogeneity (full model).

We identified genes with effect size heterogeneity, including some with ALS-increased expression in some studies (*p* < 0.05) but ALS-decreased expression in others (*p* < 0.05) (Figure S45A). Examples of such genes include *KIF9*, *ZFC3H1*, *PSMF1* and *GRM2* (Figure S45B–E). Of 16,579 protein-coding genes, there were 5930 with significant effect size heterogeneity (Cochran’s Q FDR < 0.05). Among 2563 such genes with an SMD estimate from all 16 studies, the percentage of ALS samples from the motor cortex, percentage of samples from ALS patients with sporadic disease and expression profiling technology were most frequently associated with SMD heterogeneity in single-moderator meta-regression models (*p* < 0.05 for ≥496 heterogeneous genes; QM test of moderators; Figure 2A). However, PMI most frequently explained SMD heterogeneity, with 661 genes lacking significant residual heterogeneity (*p* ≥ 0.05) in models with PMI as a single moderator (*p*≥ 0.05; QE test; Figure 2B). In contrast, only 285 genes had non-significant residual heterogeneity (*p* ≥ 0.05) in models for which expression profiling technology was a single moderator (Figure 2B). Consistent with these patterns, regression models with PMI as a single moderator frequently had a low residual heterogeneity percentage, as measured by either *I*^2^ or *H*^2^ statistics (Figure 2C–F). These conclusions were supported by three-variable meta-regression models (Figure 2G–I). In such models, both the cortex region (% motor) and PMI were significant moderator variables (*p* < 0.05) for a larger number of genes than expression profiling technology (Figure 2G). Moreover, there were 498 genes for which three moderator variables, taken together, accounted for most of the SMD heterogeneity (I-squared < 5%; Figure 2H), along with 1279 genes with non-significant residual heterogeneity in three-variable models (*p* > 0.05; QE test; Figure 2I).

### 3.4. Meta-Analysis Mitigates Differential Expression Gene Length and GC Content Bias

Meta-analysis DEGs were identified by combining SMD estimates for 16,579 protein-coding genes across the 16 datasets using a random effect inverse-variance meta-analysis model. The distribution of meta-analysis *p*-values was non-uniform and L-shaped (Figure S46A,B). The analysis identified 269 ALS-increased DEGs (FDR < 0.05, SMD ≥ 0.20) and 273 ALS-decreased DEGs (FDR < 0.05, SMD ≤ −0.20) (Supplemental Files S1 and S2; Figure S46C). There was no evidence for differential expression bias related to gene abundance (Figure S46D), gene length (Figure S46E) or GC content (Figure S46F). Alternatively, when the meta-analysis was performed using effect size estimates generated from datasets that had been normalized without GC content correction [83,101], there remained prominent differential expression bias related to GC content (Figure S47A–C), and a more negative correlation between SMD meta-estimates and both gene length and GC content (Figure S48). Meta-analysis thus mitigated differential expression bias related to the gene length and GC content, although only when effect sizes were estimated using datasets normalized with GC content correction [56,57].

### 3.5. Heterogeneity of Disease-Related Genes and Cumulative Effect Size Estimates

**Figure 3:**
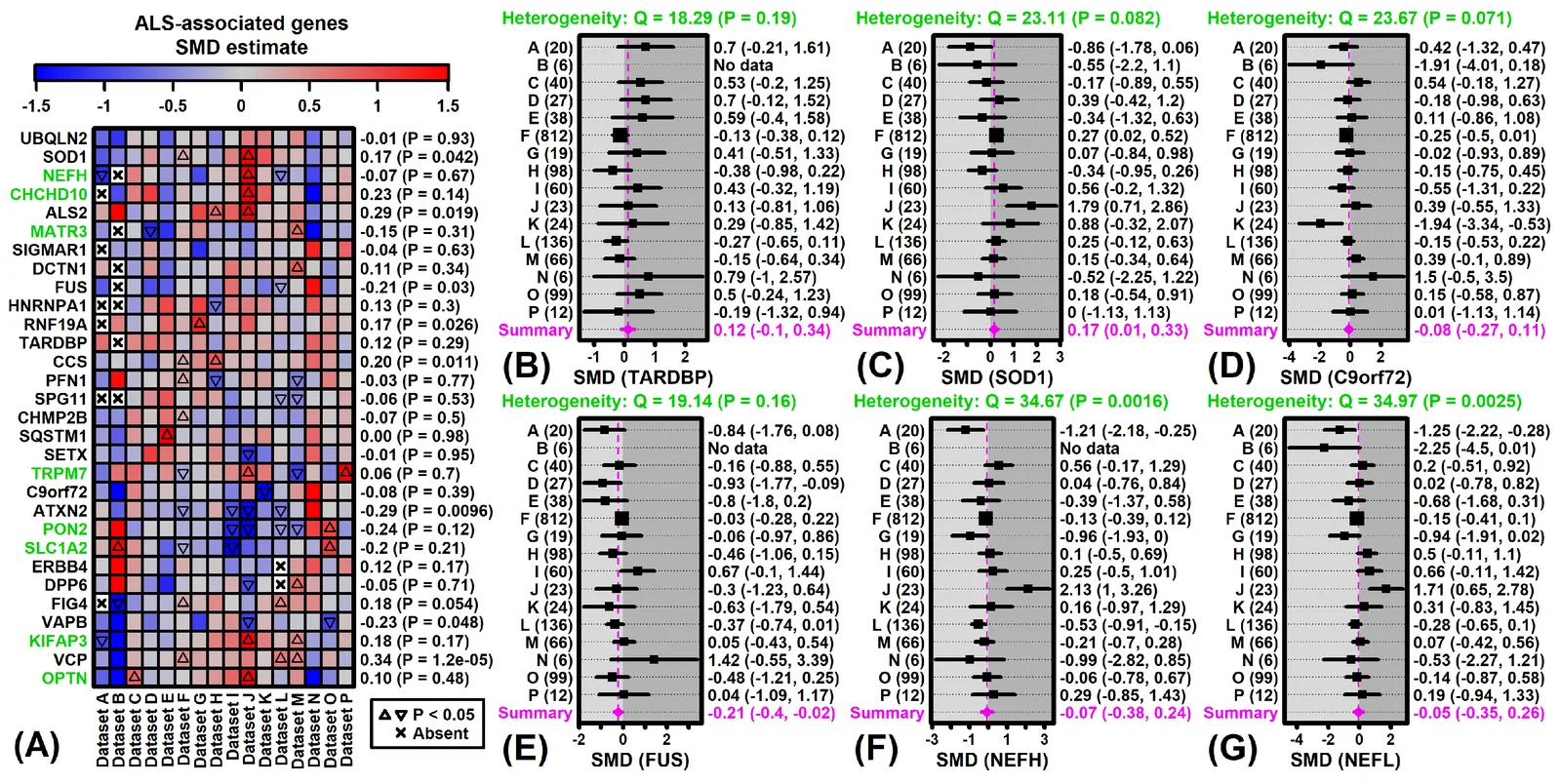
Genes frequently studied in ALS research. (**A**) ALS-related genes. Heatmaps are color-coded based on SMD estimates from each study (see legend). Genes shown in green font (left margin) are associated with significant between-study heterogeneity (FDR < 0.05, Cochran’s Q). SMD meta-estimates are listed with *p*-values in parentheses (right margin). (**B**–**G**) Forest plots. SMD estimates are shown for each study with 95% confidence intervals. The size of point estimates (squares) is proportional to the total number of samples from each dataset (left margin parentheses). The meta-estimate is represented by a magenta diamond and dashed vertical line. The SMD estimate from each study is listed with the 95% confidence interval (right margin). Cochran’s Q statistic for heterogeneity is shown with the associated *p*-value (top margin).

Several genes investigated in ALS research exhibited significant between-study heterogeneity based on Cochran’s Q statistic (e.g., *NEFH*, *CHCHD10*, *MATR3*; FDR < 0.05; Figure 3A–G). For example, expression of neurofilament heavy chain (*NEFH*) was strongly decreased in one study (SMD = −1.21, dataset *A*) but increased in another (SMD = 2.13, dataset *J*), with an overall non-significant SMD meta-estimate (SMD = −0.07, *p* = 0.67) (Figure 3F). Effect size estimates for neurofilament light chain (*NEFL*) mirrored this pattern (SMD = −0.04, *p* = 0.77; Figure 3G). In some cases, evidence from individual studies was marginal but strengthened by meta-analysis. For example, SMD estimates for superoxide dismutase 1 (*SOD1*) were non-significant but weakly positive in most individual studies, such that the aggregate SMD meta-estimate 95% lower confidence limit was greater than zero (SMD = 0.17, *p* = 0.042, FDR = 0.15; Figure 3C). Likewise, SMD estimates for FUS RNA binding protein (*FUS*) were non-significant but weakly negative in several individual studies, such that the cumulative SMD meta-estimate 95% upper confidence limit was less than zero (SMD = −0.21, *p* = 0.03, FDR = 0.30; Figure 3E).

### 3.6. ALS-Increased DEGs Are Enriched with Genes Associated with RNA Polymerase, Cell Signaling, Collagen, Oxidative Stress and SOD1 Expression

**Figure 4:**
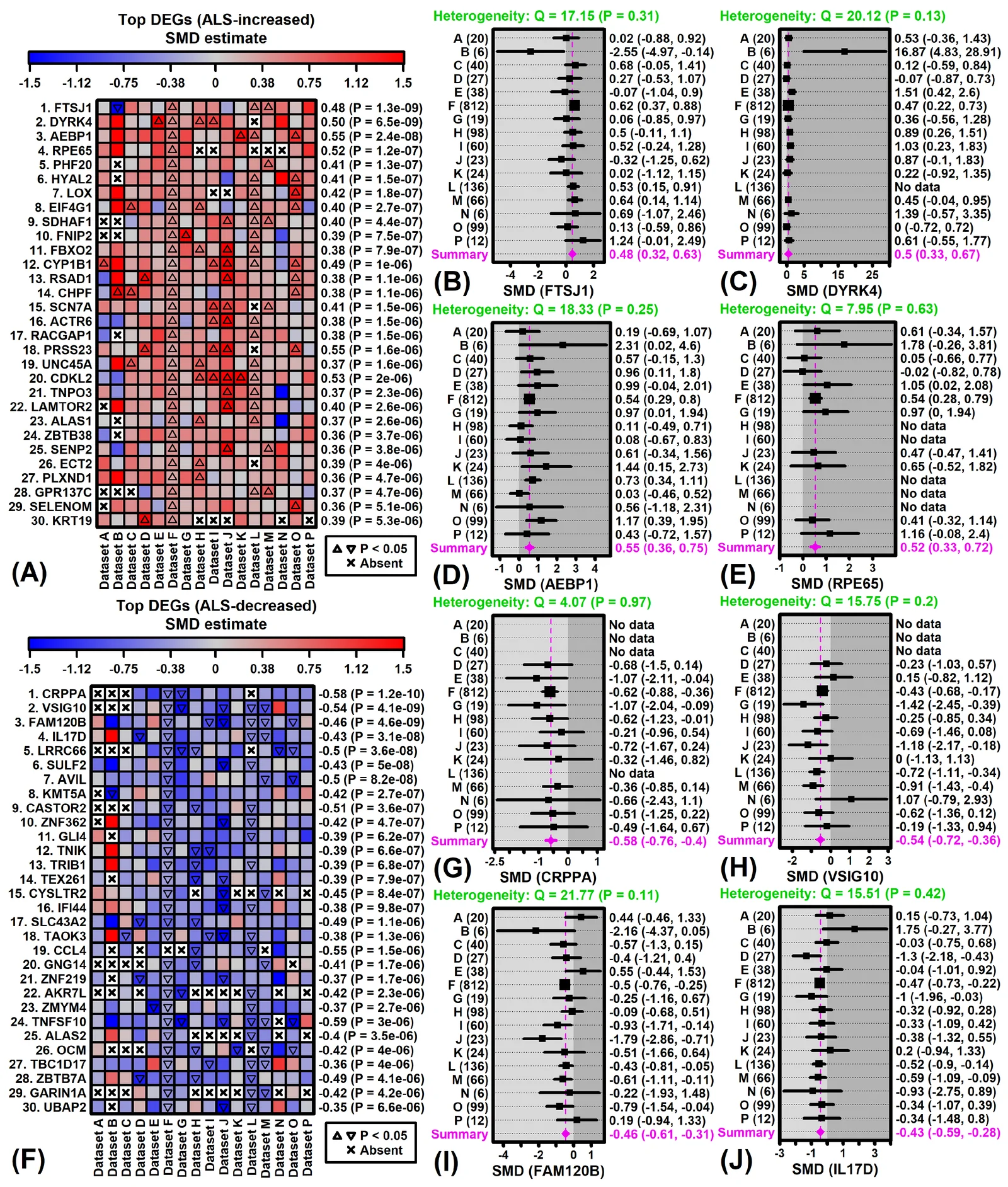
Top-ranked differentially expressed genes (DEGs). (**A**) ALS-increased DEGs. (**F**) ALS-decreased DEGs. In (**A**,**F**), heatmaps are color-coded based on SMD estimates from each study (see legend). SMD meta-estimates are listed with *p*-values in parentheses (right margin). (**B**–**E**,**G**–**J**) Forest plots. SMD estimates are shown for each study with 95% confidence intervals. The size of point estimates (squares) is proportional to the total number of samples from each dataset (left margin parentheses). The meta-estimate is represented by a magenta diamond and dashed vertical line. The SMD estimate from each study is listed with the 95% confidence interval (right margin). Cochran’s Q statistic for heterogeneity is shown with the associated *p*-value (top margin).

Top-ranked ALS-increased DEGs identified by meta-analysis were increased in most but not all of the 16 datasets (Figure 4A–E). Although some ALS-increased DEGs were associated with moderate (0.50 < SMD < 0.80) effect size estimates (e.g., *DYRK4*, *AEBP1*, *RPE65* and *PRSS23*) (Figure 4A–E), most were associated with small magnitude estimates (e.g., *FTSJ1*, *PHF20*, *HYAL2* and *LOX*; Figure 4A–E). Complete lists of protein-coding and non-coding ALS-increased DEGs are included in Table S1.

**Figure 5:**
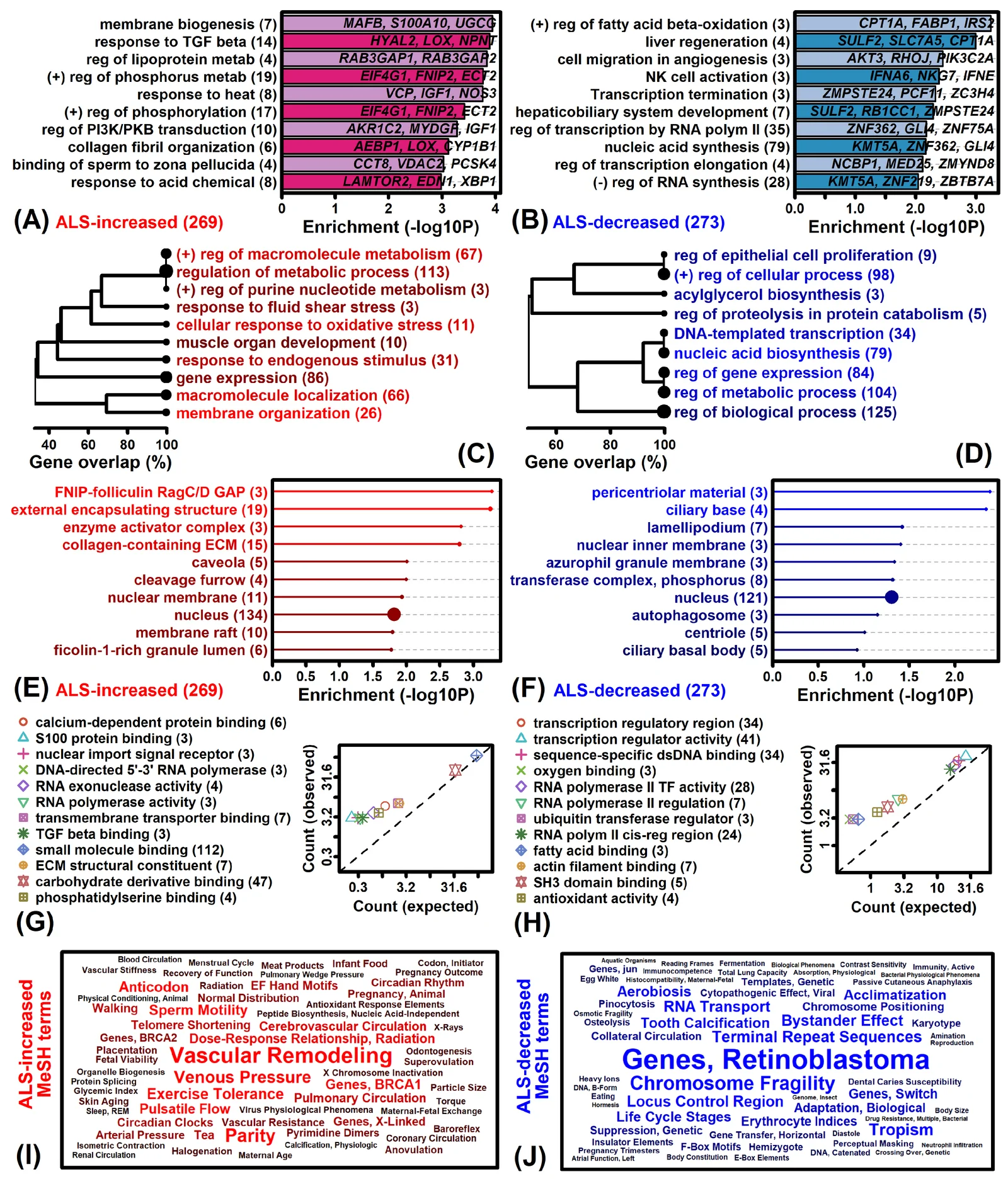
ALS DEG enrichment analysis. (**A**,**B**) Top-ranked GO BP terms (A: ALS-increased DEGs, B: ALS-decreased DEGs). Enrichment is shown on the horizontal axis (conditional hypergeometric test). The number of DEGs associated with each term is given in parentheses (left margin). Example DEGs are listed within each figure. (**C**,**D**) GO BP term cluster analysis (C: ALS-increased DEGs, D: ALS-decreased DEGs). Enriched terms were clustered based on percent overlap between gene sets associated with each term (horizontal axis). Representative terms are shown from each cluster group. The number of DEGs associated with each representative term is given in parentheses. Terms associated with more DEGs are represented by larger leaf node circles. Terms associated with more significant enrichment are displayed in brighter fonts (red or blue). (**E**,**F**) Top-ranked GO CC terms (E: ALS-increased DEGs, F: ALS-decreased DEGs). Enrichment is shown on the horizontal axis (conditional hypergeometric test). The number of DEGs associated with each term is given in parentheses (left margin). Terms associated with more DEGs are represented by larger symbols within each figure. Terms associated with more significant enrichment are displayed in brighter fonts (red or blue). (**G**,**H**) Top-ranked GO MF terms (G: ALS-increased DEGs, H: ALS-decreased DEGs). Scatterplots show the observed and expected number of DEGs associated with each GO MF term (see legend). The dotted line is shown for reference (slope = 1, intercept = 0). The number of DEGs associated with each term is given in parentheses (left margin) (**I**,**J**) Top-ranked MeSH terms ((**I**) ALS-increased DEGs, (**J**) ALS-decreased DEGs). MeSH terms having more significant enrichment are shown with larger and brighter fonts (red or blue).

The 269 ALS-increased DEGs were most significantly enriched with respect to GO BP terms involving membrane biogenesis, response to TGF beta, regulation of lipoprotein metabolism, collagen fibril organization and cellular response to oxidative stress (Figure 5A,B). Approximately 50% of ALS-increased DEGs (134 of 269) were localized to the nucleus (Figure 5E). Enriched GO MF terms included RNA polymerase activity, calcium-dependent protein binding, S100 protein binding, and TGF beta binding (Figure 5G). MeSH terms associated with ALS-increased DEGs included vascular remodeling and telomere shortening (Figure 5I). Comparison to other annotation databases demonstrated associations between ALS-increased DEGs and MYC regulation, TGB1-TGFB1R signaling, down-regulation in Alzheimer’s disease and a computationally defined SOD1 expression neighborhood [120] (Table S1). There was significant overlap between ALS-increased DEGs and drug signatures, including those linked to loxapine (tricyclic antipsychotic), isoimperatorin (small-molecule anti-inflammatory) and roxarsone (organic arsenic additive) (Table S1).

### 3.7. ALS-Decreased DEGs Are Enriched with Genes Encoding Nuclear Proteins That Support RNA Synthesis and Transcription

ALS-decreased DEGs identified by meta-analysis demonstrated consistent patterns in most datasets (Figure 4F–J). There were some ALS-decreased DEGs (*CRPPA*, *VSIG10*, *LRRC66*) for which the summary effect size was moderate in magnitude (i.e., 0.50 < SMD < 0.80) (Figure 4F–H). However, most top-ranked ALS-decreased DEGs were associated with small but significant effect size estimates (e.g., *FAM120B* and *IL17D*) (Figure 4F,IJ). Among non-coding DEGs, expression of *MIR9-1HG* (MIR9-1 host gene) was most significantly decreased (SMD = −0.497, *p* = 1.51 × 10^−08^, FDR = 7.54 × 10^−05^). Complete lists of protein-coding and non-coding ALS-decreased DEGs are provided in Table S2.

ALS-decreased DEGs were enriched with respect to GO BP terms such as fatty acid beta-oxidation, cell migration in angiogenesis, transcription termination and DNA-templated transcription (Figure 5B,D). Approximately 45% of ALS-decreased DEGs (121 of 273) were localized to the nucleus (Figure 5G). A larger than expected fraction of ALS-decreased DEGs were associated with GO MF terms such as transcription regulatory region, sequence-specific dsDNA binding and antioxidant activity (Figure 5H). ALS-decreased DEGs were frequently associated with MeSH terms such as terminal repeat sequences, chromosome fragility and RNA transport (Figure 5J).

### 3.8. Significant Overlap Between Meta-Analysis DEGs Identified from Cortex with Those from Spinal Cord and Whole Blood

**Figure 6:**
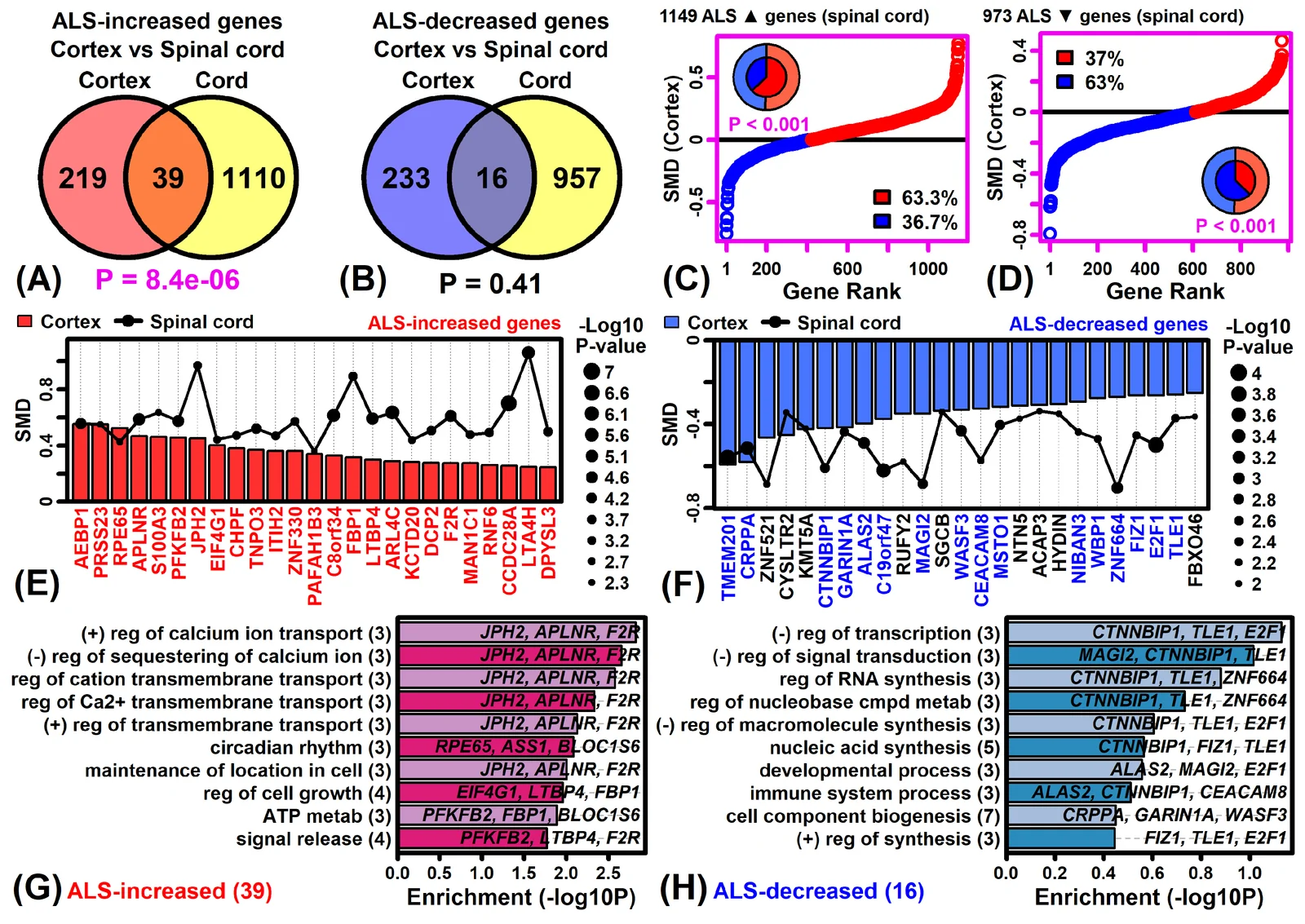
Comparison to DEGs from spinal cord transcriptome meta-analysis [66]. (**A**,**B**) Venn diagrams. Overlap is shown between meta-analysis ALS DEGs from cortex (current study) and spinal cord (prior study). Significance of overlap was evaluated using Fisher’s exact test (bottom margin). Both gene sets are defined based on the same thresholds (FDR < 0.05 with SMD ≥ 0.20 or SMD ≤ −0.20). (**C**,**D**) Cortex SMD estimates of spinal cord DEGs (blue: SMD < 0 in cortex; red: SMD > 0 in cortex). The percentage of genes with SMD > 0 in cortex (red) or SMD < 0 in cortex is shown (upper left). This percentage is also represented by the pie chart. The outer pie chart (lower right) shows the percentage of genes with SMD > 0 in the cortex (red) and SMD < 0 in the cortex (blue) among all genes from both meta-analyses. The *p*-value was obtained by comparing inner and outer pie chart frequencies (Fisher’s exact test). (**E**) ALS-increased DEGs (cortex and spinal cord). (**F**) ALS-decreased DEGs (cortex and spinal cord). In (**E**,**F**), bars show SMD estimates in the cortex and circles denote SMD estimates in spinal cord. The size of a circle is proportional to the *p*-value associated with the SMD estimate from the spinal cord meta-analysis (see scale, right margin). (**G**,**H**) Top-ranked GO BP terms. GO BP terms enriched with respect to (**G**) ALS-increased DEGs or (**H**) ALS-decreased DEGs from both cortex and spinal cord meta-analyses. Enrichment is shown on the horizontal axis (conditional hypergeometric test). The number of DEGs associated with each term is given in parentheses (left margin). Example DEGs are listed within each figure.

Meta-analysis DEGs were compared to those from recent meta-analysis studies of post-mortem spinal cord from ALS patients and CTL subjects [66,67]. When the same significance thresholds are applied (FDR < 0.05 with SMD ≥ 0.20 or SMD ≤ −0.20), fewer DEGs are identified from ALS cortex than from bulk-processed spinal cord samples (1149 ALS-increased DEGs; 982 ALS-decreased DEGs). There was significant overlap between DEGs increased in the ALS cortex and spinal cord (*p* < 0.001; Figure 6A) and a significantly high fraction of ALS-increased DEGs from the spinal cord had positive SMD estimates (SMD > 0) in cortex (*p* < 0.001; Figure 6C). There was no significant overlap between ALS-decreased DEGs from the spinal cord and cortex (*p* = 0.41; Figure 6B), although nearly two-thirds of ALS-decreased DEGs from the spinal cord had negative SMD estimates (SMD < 0) in the cortex (*p* < 0.001; Figure 6D). Examples of ALS-increased DEGs from both tissues include *AEBP1*, *PRSS23* and *RPE65* (Figure 6E), whereas ALS-decreased DEGs from both tissues included *TMEM201*, *CRPP1* and *CTNNBIP1* (Figure 6F). ALS-increased DEGs from both tissues were associated with positive regulation of calcium ion transport, the circadian rhythm and maintenance of the location in the cell (Figure 6G). ALS-decreased DEGs from both tissues were associated with negative regulation of transcription, negative regulation of signal transduction and regulation of RNA synthesis (Figure 6H).

Cortex DEGs were next compared to those identified from LCM-dissected spinal cord motor neurons from ALS patients and CTL subjects [67]. Given the same significance threshold (FDR < 0.05 with SMD ≥ 0.20 or SMD ≤ −0.20), fewer DEGs were identified from LCM-dissected motor neurons (155 ALS-increased, 225 ALS-decreased). ALS-increased DEGs from the cortex did not overlap significantly with DEGs from spinal motor neurons (*p* = 0.37; Figure S49A), although a significant majority (61.3%) of the 155 ALS-increased DEGs from spinal motor neurons had positive SMD estimates (SMD > 0) from the cortex (*p* < 0.001; Figure S49C). Examples of increased DEGs from both tissues included *APLNR*, *RASGRF2* and *MAFB* (Figure S49E). There was no significant overlap between ALS-decreased DEGs from the cortex and LCM-dissected spinal motor neurons (Figure S49B,D), but examples of decreased DEGs from both tissues include *SLC43A2*, *ZNF521* and *DDX56* (Figure S49F).

DEGs from the cortex were next compared with those identified from a large-scale study of whole blood from ALS patients and CTL subjects [121]. There was no significant overlap between ALS-increased DEGs from both tissue types (*p* = 0.065; Figure S50A) although a significant majority (56.6%) of ALS-increased DEGs from the blood were correspondingly increased (SMD > 0) in the cortex (Figure S50C). Examples of ALS-increased DEGs from both tissues included *ANXA1*, *TNFRSF12A* and *SMM29* (Figure S50E). The 18 DEGs increased in both tissues were most significantly enriched with genes involved in positive regulation of cellular process and macromolecule metabolism (Figure S50G). There was significant overlap between ALS-decreased DEGs from both tissues (*p* = 0.027; Figure S50B), although ALS-decreased DEGs from the blood were not more frequently decreased (SMD < 0) in the cortex (*p* = 0.59; Figure S50D). ALS-decreased DEGs from both tissues included *PTGDR2*, *ZNF362* and *ZNF580* (Figure S50F). Such DEGs were most significantly enriched with respect to genes involved in RNA polymerase II transcription, positive regulation of RNA metabolism, and DNA damage response (Figure S50H).

Finally, we evaluated the overlap of all ALS DEG sets described above, including those from the current study (cortex), those from bulk spinal cord [66] or LCM-dissected spinal cord motor neurons [67], and those from whole blood [121]. This comparison identified four ALS-increased DEGs shared among all tissue types (*KCTD20*, *DCP2*, *CCDC28A* and *TNFRSF12A*), along with two ALS-decreased DEGs shared among the three tissues (*MSTO1*, *ALAS2*).

### 3.9. Meta-Analysis DEGs from Cortex Do Not Overlap Significantly with Those near ALS-Associated Loci from GWA Studies

Comparison was made to ALS-associated genes linked to ALS in GWA studies (Figure S51A) [60]. Among 238 such genes, there was no significant trend towards ALS-increased or ALS-decreased expression (Figure S51B) and no significant overlap with respect to all DEGs from the cortex, ALS-increased DEGs or ALS-decreased DEGs (Figure S51C–E). DEGs identified from the meta-analysis were not more likely than other genes to be in close genomic proximity to loci identified from GWA studies (Figure S51F–I). We identified GO and KEGG terms overrepresented among genes near ALS GWA loci, but there was no significant tendency for such terms to be similarly enriched with respect to ALS-increased or ALS-decreased DEGs (Figure S51J–M).

### 3.10. Transcription Factor Binding Sites near ALS-Increased and ALS-Decreased DEGs

Disease-related changes in gene expression may be driven in part by sequence-specific binding of TFs to their genomic recognition sites. Along these lines, both ALS-increased and ALS-decreased DEGs were associated with RNA polymerase and gene expression or transcription (Figure 5G,H). We screened a library of 970 TF binding sites [118] to identify those with increased proximity to ALS-increased or ALS-decreased DEGs transcription start sites (TSSs). Motifs recognized by HMX1, GMEB1 and ZBTB7C were 10–20% closer to ALS-increased DEGs compared to non-DEGs (*p* ≤ 0.004; two-sample two-tailed *t*-test), although this difference was non-significant following FDR correction for multiple hypothesis testing (FDR ≥ 0.20; Figure S52A). Likewise, motifs recognized by ZNF454, ZNF143 and TEAD2 were 10-20% closer to ALS-decreased DEGs compared to non-DEGs (*p* ≤ 0.007; two-sample two-tailed *t*-test), but again, these findings were non-significant following FDR correction (FDR ≥ 0.30; Figure S52B). Complete lists of top-ranked motifs are provided in Tables S1 and S2.

### 3.11. ALS-Increased DEGs Are Expressed by Interneurons, Endothelial Cells and Microglia While ALS-Decreased DEGs Are Expressed by Glial Cells (Astrocytes, Oligodendrocytes)

**Figure 7:**
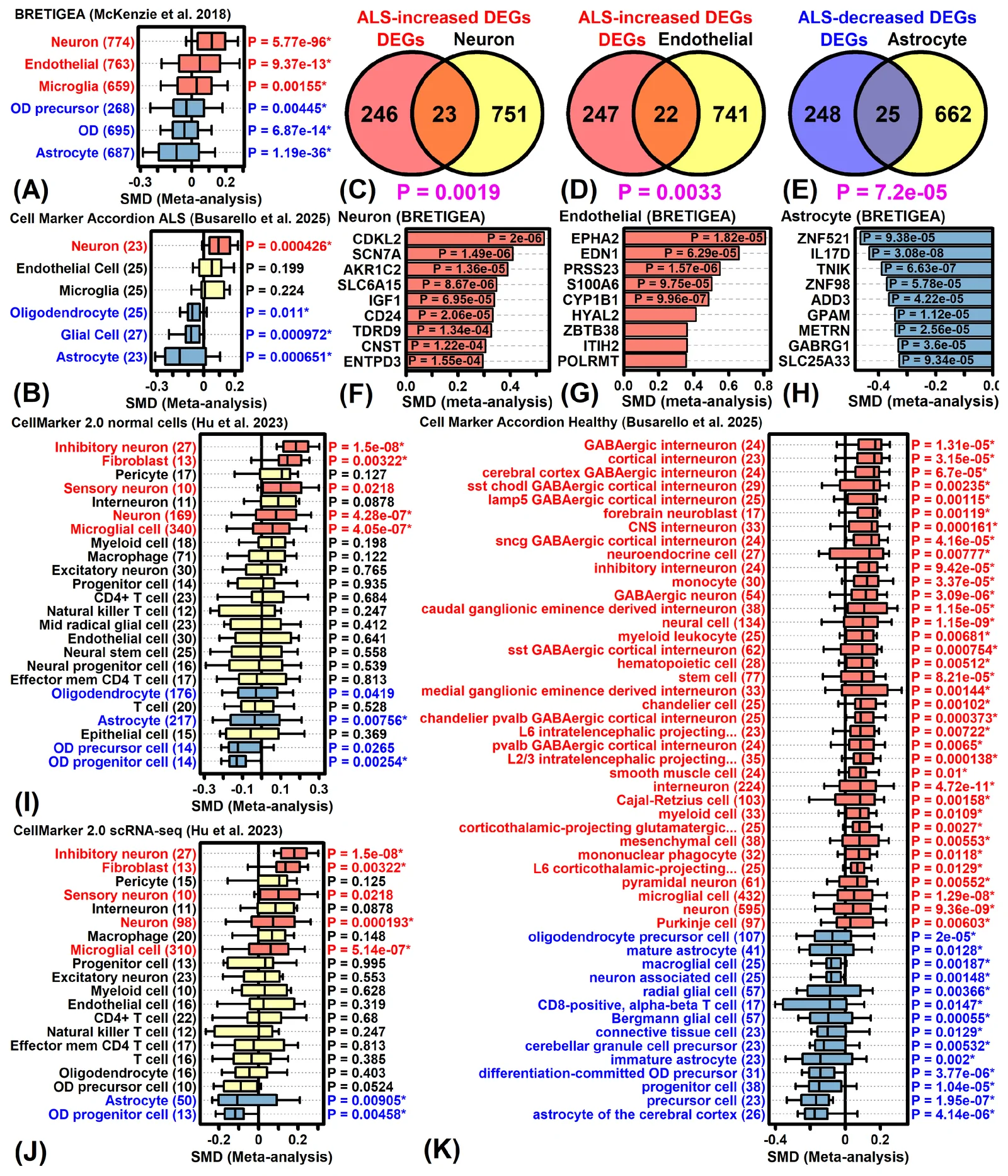
Cell type marker gene analysis. (**A**) Brain cell type specific gene expression analysis (BRETIGEA). (**B**) Cell Marker Accordion ALS-related marker genes. (**C**–**E**) Venn diagrams. Overlap is shown between ALS DEGs and BRETIGEA marker genes for the indicated cell type. Significance of overlap was evaluated using Fisher’s exact test (bottom margin). (**F**) Neuron marker genes (BRETIGEA). All listed genes were ALS-increased DEGs (FDR < 0.05, SMD ≥ 0.20). (**G**) Endothelial marker genes (BRETIGEA). All listed genes were ALS-increased DEGs (FDR < 0.05, SMD ≥ 0.20). (**H**) Astrocyte marker genes (BRETIGEA). All listed genes were ALS-decreased DEGs (FDR < 0.05, SMD ≤ −0.20). (**I**) CellMarker 2.0 markers (normal non-disease tissues). (**J**) CellMarker 2.0 markers from sc-RNAseq studies (normal non-disease tissues). (**K**) Cell Marker Accordion markers (normal non-disease tissues). In (**A**,**B**,**I**,**J**,**K**), boxplots outline the middle 50% of marker gene SMD estimates (whiskers: 10th to 90th percentiles). Regions are listed with the number of marker genes given in parentheses (left margin). *p*-values (right margin) were generated by testing whether marker gene SMD estimates differ from all other genes included in the meta-analysis (Wilcoxon rank sum test). An asterisk (*) is used to indicate which *p*-values meet significance at an FDR < 0.05 threshold.

Meta-analysis DEGs were evaluated to determine whether cell type marker genes exhibited trends toward ALS-increased or ALS-decreased expression. When utilizing the BRETIGEA database [61], there was a significant pattern of ALS-increased expression among neuron marker genes (*p* = 5.77 × 10^−96^; Wilcoxon rank sum test; Figure 7A), with a similar pattern of ALS-decreased expression among astrocyte markers (*p* = 1.19 × 10^−36^; Figure 7A,E). Consistent with this, there was significant overlap between ALS-increased DEGs and neuron marker genes (e.g., *CDKL2*, *SCN7A* and *AKR1C2*; Figure 7C,F), and significant overlap between ALS-decreased DEGs and astrocyte marker genes (e.g., *ZNF521*, *IL17D* and *TNIK*; Figure 7E,H). There were also less substantial but significant trends toward increased expression of endothelial and microglia marker genes (Figure 7A,D) and decreased expression of OD and OD precursor genes (Figure 7A). Endothelial marker genes most strongly increased in the ALS cortex included *EPHA2*, *EDN1* and *PRSS23* (Figure 7D,G).

Additional marker gene databases were screened to provide a higher-resolution view of cell type-specific shifts in gene expression [62,63]. CellMarker 2.0 gene sets [62] associated with inhibitory neuron, fibroblast and microglia were each increased in the ALS cortex (FDR ≤ 0.05; Figure 7I,J), whereas those associated with the OD progenitor cell, OD precursor cell and astrocyte were decreased in the ALS cortex (FDR < 0.05; Figure 7I,J). The Cell Marker Accordion database provided the most detailed grouping of gene set markers [63]. Nearly all of the most strongly ALS-increased gene sets from this database were associated with interneurons (Figure 7K). For example, among 24 genes associated with GABAergic interneurons, the majority (>75%) were associated with positive SMD estimates (Figure 7K). The most strongly ALS-decreased gene sets were associated with astrocytes, precursor or progenitor cells, OD and glial cells (Figure 7K). Among those genes linked to cerebral cortex astrocytes, the majority (>75%) were associated with negative SMD estimates (SMD < 0) (e.g., *ABLIM1*, *ZHX3*; Figure 7K). Among Cell Marker Accordion genes linked to astrocytes from studies of the ALS post-mortem brain, the majority were again associated with negative SMD estimates (e.g., *SLC4A4*, *OPHN1*, *NHSL1*; Figure 7B).

### 3.12. Increased Expression of L3, L5 and L6 Cortical Layer Genes and Decreased Expression of L1 and White Matter Genes in ALS Post-Mortem Cortex

**Figure 8:**
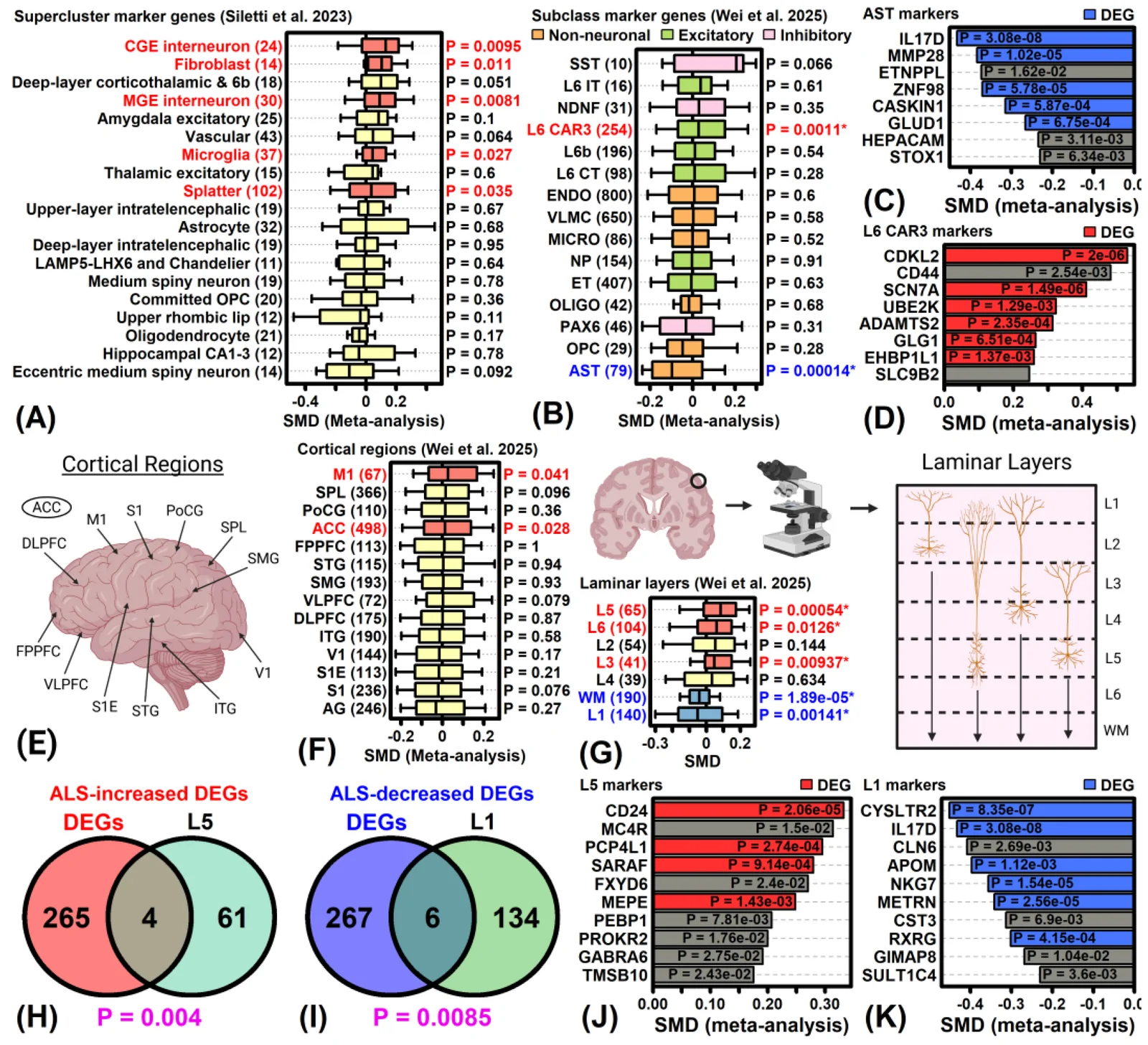
Human cortex snRNA-seq and spatial transcriptomic regional marker genes. (**A**) Supercluster gene SMD estimates [64]. (**B**) Subclass gene SMD estimates [65]. (**C**) Astrocyte (AST) marker genes. (**D**) Oligodendrocyte precursor cell (OPC) marker genes. (**E**) Cortical regions. The illustration shows locations of the angular gyrus (AG), anterior cingulate cortex (ACC), dorsolateral prefrontal cortex (DLPFC), frontopolar prefrontal cortex (FPPFC), inferior temporal gyrus (ITG), postcentral gyrus (PoCG), primary motor cortex (M1), primary somatosensory cortex (S1), primary somatosensory cortex of the eardrum (S1E), primary visual cortex (V1), superior parietal lobule (SPL), superior temporal gyrus (STG), supramarginal gyrus (SMG) and ventrolateral prefrontal cortex (VLPFC). (**F**) Cortical region SMD estimates [64]. (**G**) Laminar layer SMD estimates [64]. (**H**,**I**) Venn diagrams. Overlap is shown between DEGs and marker genes for the indicated layers. Significance of overlap was evaluated using Fisher’s exact test (bottom margin *p*-values). (**J**) L5 marker genes. (**K**) L1 marker genes. In (**A**,**B**,**F**,**G**), boxplots outline the middle 50% of marker gene SMD estimates (whiskers: 10th to 90th percentiles). Group or regional categories are listed (left margin), with the number of marker genes given in parentheses. *p*-values were generated by testing whether marker gene SMD estimates differ from all other genes included in the meta-analysis (* FDR < 0.05; Wilcoxon rank sum test). In (**C**,**D**,**J**,**K**), marker genes with the lowest or highest SMD meta-estimates are shown (red bars: FDR < 0.05 with SMD ≥ 0.20; blue bars: FDR < 0.05 with SMD ≤ −0.20). *p*-values associated with SMD estimates are indicated where space allows. Figure (**E**) and parts of (**G**) were created using Biorender (https://www.biorender.com/).

A recent snRNA-seq study of 3.34 million cells from post-mortem adult brains (mostly cerebral cortex) had classified cells into 31 superclusters spanning a broad range of CNS cell diversity [64]. Among these superclusters, genes belonging to several (annotated as CGE/MGE interneuron, fibroblast, microglia) exhibited trends towards ALS-increased expression, although these trends were non-significant following multiple test correction (Figure 8A). Stronger trends were seen among 16 subclasses defined by a recent snRNA-seq study of 1.1 million nuclei from five adult brains without neurological disease (Figure 8B) [65]. Marker genes associated with the L6 CAR3 subclass were on average associated with ALS-increased expression (FDR < 0.05; Figure 8B). ALS-increased DEGs associated with the L6 CAR3 subclass included *CDKL2*, *SCN7A* and *UBE2K* (Figure 8D). Conversely, marker genes associated with the astrocyte subclass were on average associated with ALS-decreased expression (FDR < 0.05; Figure 8B). ALS-decreased DEGs associated with the astrocyte subclass included *IL17D*, *MMP28* and *ZNF98* (Figure 8C).

This same prior study [65] had used spatial transcriptome sequencing from nine donors to localize gene expression to cortical regions (Figure 8E) and laminar layers (Figure 8G). Marker genes associated with the primary motor cortex (M1) and anterior cingulate cortex (ACC) were on average increased in the ALS cortex (*p* ≤ 0.041), although these trends were non-significant following multiple test correction (FDR > 0.05) (Figure 8F). Stronger trends were seen with respect to laminar layers (Figure 8G). Marker genes linked to the L3, L5 and L6 layers were frequently increased in the ALS cortex (*p* ≤ 0.0126), whereas genes linked to the L1 layer and white matter were often decreased in the ALS cortex (*p* ≤ 0.00141; Figure 8G). There was significant overlap between ALS-increased DEGs and L5 marker genes (e.g., *CD24*, *PCP4L1*, *SARAF*; *p* = 0.004; Figure 8H,J) and significant overlap between ALS-decreased DEGs and L1 marker genes (e.g., *CYSLTR2*, *IL17D*, *APOM*; *p* = 0.0085; Figure 8I,K).

## 4. Discussion

**Figure 9:**
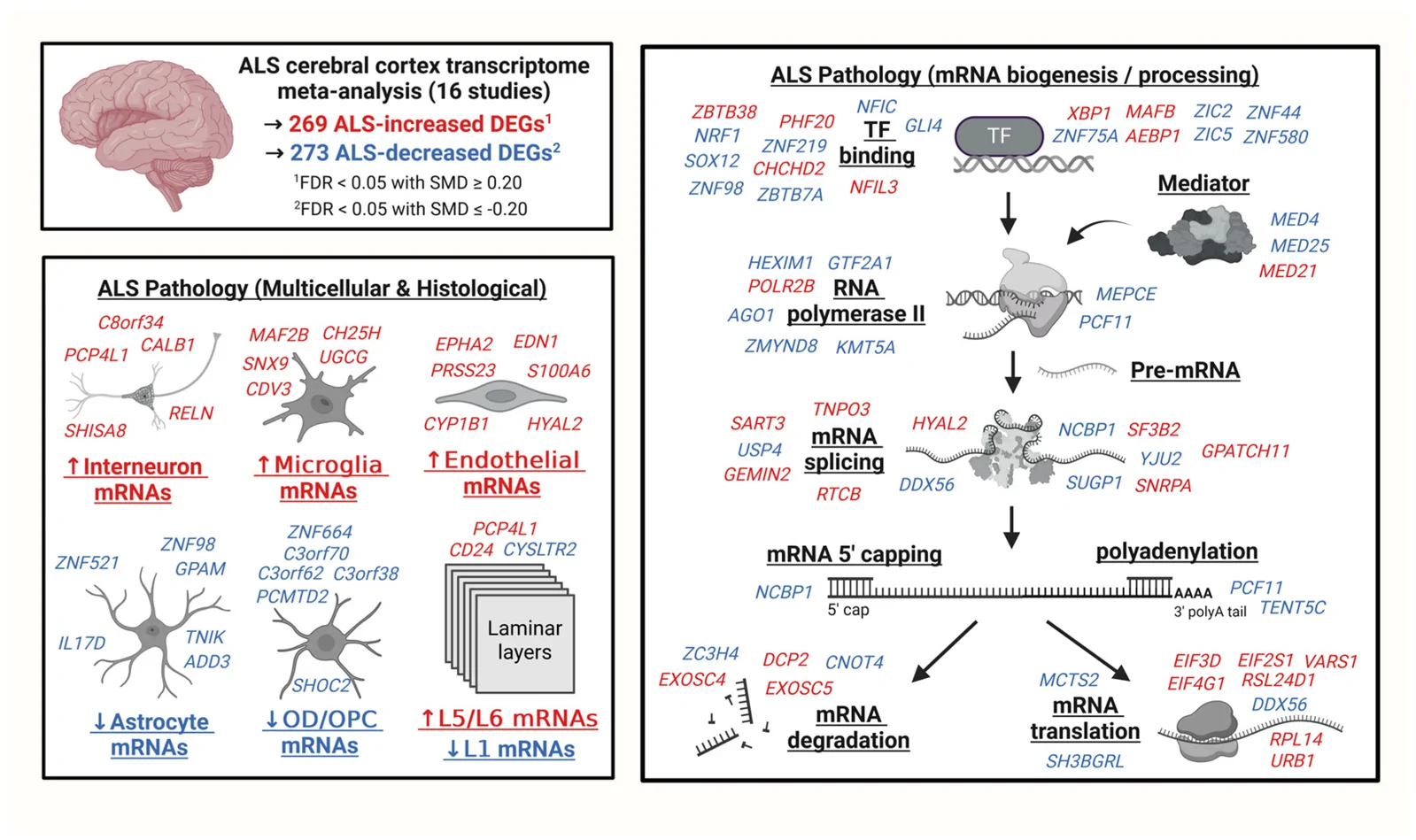
Pathology of end-stage ALS at the multicellular, histological and intracellular levels. This study identified 269 ALS-increased DEGs (FDR < 0.05, SMD ≥ 0.20) and 273 ALS-decreased DEGs (FDR < 0.05, SMD ≤ −0.20) based on meta-analysis of effect size estimates (ALS vs. CTL) from 16 transcriptome studies of post-mortem cortex from ALS patients and control subjects. The figure summarizes cell types, cortical regions and intracellular processes associated with DEGs based on over-represented gene annotations and comparison to other human brain data resources (snRNA-seq and/or spatial transcriptomics). Broad categories are underlined with associated DEGs shown in italicized font (red: ALS-increased DEGs; blue: ALS-decreased DEGs). Created using Biorender (https://www.biorender.com/).

The aggregation of evidence from multiple studies can separate robust findings from those that are non-repeatable [45]. Post-mortem transcriptome studies of the human cortex from ALS patients, generated from multiple technologies, have accrued over two decades. Despite this, there have been few attempts to reconcile conflicting results from different studies, or even to jointly analyze available data using a consistent methodological framework. This study reports findings from a meta-analysis of 16 studies that evaluated the post-mortem cortex in ALS patients and control subjects (Table 1). Altogether, the analysis incorporates expression data from 1118 ALS samples (639 unique patients) and 369 control samples (244 unique individuals). Since the current study focused on bulk (or pseudobulk) mRNA profiles, we cannot fully disentangle histological and cellular shifts underlying the identified ALS signature (269 ALS-increased DEGs, 273 ALS-decreased DEGs). Nonetheless, by leveraging human CNS snRNA-seq and spatial transcriptomic resources [61,64,65], our results support a working model of end-stage pathology (Figure 9). This model involves transcriptional dysregulation and disease-related changes in tissue structure and cellular composition, with mRNA profiles pointing towards diminution of the L1 layer and loss of glial cells (astrocytes and oligodendrocytes), together with relative expansion of deep layers (L5 and L6), interneurons, microglia and endothelial cells.

This study highlights between-study heterogeneity in prior work focusing on expression profiling of ALS patient post-mortem tissues. Approximately one-third of protein-coding genes (5930 of 16,579) evaluated exhibit significant between-study effect size heterogeneity (FDR < 0.05, Cochran’s Q statistic). Surprisingly, expression profiling technology was not the dominant factor associated with effect size heterogeneity, despite the inclusion of data from microarray, RNA-seq and scRNA-seq platforms (Table 1). Rather, our results demonstrate that other factors, such as PMI and cortical region, were more frequently associated with heterogeneous effect size estimates (Figure 2). The association between PMI and effect size heterogeneity has implications for future work. Among 16 included studies, the average PMI of samples ranged almost 6-fold, from 5 to 29.5 h, with more recent studies reporting a higher average PMI (>20 h) [14,20,29,53]. PMI prolongation likely contributes to RNA degradation and may amplify downstream biases [122,123]. We found, for example, that commonly used normalization approaches that do not adjust for GC content [83,101] resulted in differential expression patterns correlating with the GC content (and length) of transcripts (Figures S43 and S44). Differential expression GC bias was not resolved even after meta-analysis was used to synthesize effect sizes (Figure S47C). However, utilization of alternative normalization algorithms correcting for GC content bias [56,57] attenuated both GC content and gene length biases in differential expression patterns from individual datasets (Figures S41 and S42), with complete resolution of these biases following effect size integration by meta-analysis (Figure S46E,F). These results support the idea that, although GC content normalization may not be essential when profiling RNA samples generated under optimal laboratory conditions, such normalization algorithms may be needed when analyzing post-mortem cortex samples, particularly when PMI is prolonged.

Both ALS-increased and -decreased DEGs from this study encoded proteins involved in transcription and mRNA surveillance (Figure 9). This result had been emphasized by one early ALS cortex transcriptome study as well [78] and resonates with emerging work that has linked RNA dysregulation to disease pathology [124,125,126,127,128]. Several ALS-increased DEGs (*ZNF302*, *ZNF585A*, *ZNF189*) and ALS-decreased DEGs (*ZNF362*, *ZNF219*, *ZNF580*) encoded C2H2 zinc finger factor transcription factors (TFs), although we were unable to identify a significant TF-recognition motif with significantly increased proximity to ALS-increased or ALS-decreased DEGs (Figure S52). ALS-decreased DEGs included *MED4* (mediator complex subunit 4) and *MED25* (mediator complex subunit 25), each encoding a subunit of the mediator complex, which is involved in the recruitment of RNA polymerase II to gene promoters [129] and its coordination with transcription factors [130]. Mediator complex dysfunction has been implicated in neurodevelopmental [131] and neurodegenerative diseases [130]. Several DEGs were associated with RNA polymerase II and the initiation, elongation or termination of transcription; for example, expression of *PCF11* was decreased in the ALS post-mortem cortex (SMD = −0.30, *p* = 1.19 × 10^−04^, FDR = 0.009). *PCF11* encodes a component of the core cleavage and polyadenylation complex [132] and facilitates dissociation between DNA and RNA polymerase II [133], leading to transcript termination. RNA polymerase II is disrupted in familial models of ALS [134,135,136,137] and co-localizes with FUS and TDP-43 at sites of transcription arrest-associated DNA damage [138]. Interestingly, we noted decreased expression of *NCBP1* (SMD = −0.33, *p* = 1.32 × 10^−05^, FDR = 0.002) along with increased expression of *DCP2* (SMD = 0.28, *p* = 3.51 × 10^−04^; FDR = 0.02). NCBP1 encodes a nuclear cap-binding complex subunit [139], which adds 7-methylguanosine to the 5′ end of pre-mRNA, thereby protecting mRNAs from 5′-3′ exonuclease degradation [140]. *DCP2*, on the other hand, encodes an active enzyme of the decapping complex, which interacts with TDP-43 [141] and acts in a transcript-selective manner to promote mRNA breakdown [142,143]. Notably, *DCP2* was one of only four genes with evidence for increased expression in the ALS cortex (current study), spinal cord [66,67] and whole blood [121]. Finally, our meta-analysis identified increased expression of two genes (*EXOSC4*, *EXOSC5*) that encode components of the RNA exosome, which coordinates 3′-to-5′ RNA degradation [144] in both the cytoplasm and nucleus [145]. This RNase complex is critical for degrading defective repeat RNA in *C9orf72* mutation carriers [146], and in invertebrate models, loss of RNA exosome activity impairs RNA surveillance to engender a neurodegenerative phenotype [147]. DEGs identified by meta-analysis were thus connected to multiple phases of the mRNA lifecycle that have been linked to ALS and other neurodegenerative processes (Figure 9).

We observed loss of mRNAs expressed by glial support cells in the post-mortem ALS cortex, with the strongest trends observed for astrocytes, but significant trends were also observed with respect to oligodendrocytes (ODs), OD precursor cells and OD progenitor cells (Figure 7). Our findings thus support prior work linking such cell types to disease pathology [148,149,150,151,152]. ALS-decreased DEGs identified by meta-analysis, for example, overlapped significantly with astrocyte marker genes (e.g., *ZNF521*, *IL17D*, *TNIK*; Figure 7E,H). ALS involves development of reactive astrocytes that can release extracellular vesicles and neurotoxic factors to promote motor neuron degeneration [153,154,155]. This can result in DNA damage to motor neurons and contribute to neuronal excitability by interrupting astrocyte clearance of glutamate and the potassium cation [156,157]. Loss of astrocyte marker mRNAs in the ALS cortex may thus reflect disease-related shifts of astrocytes to dysfunctional and neurotoxic phenotypes, although degeneration of astrocytes surrounding motor neurons has also been seen in the SOD1-G93A mouse model [158]. Similarly, loss of OD-derived mRNAs observed in this study, and supported by prior ALS transcriptome analyses [66,79], may reflect the emergence of disease-related OD phenotypes and/or degeneration of mature ODs and their myelin-generating ability [66]. The latter process may be driven by oxidative stress or inflammation [159] and is supported by prior work demonstrating gray matter demyelination within the ALS motor cortex and spinal cord [160]. Increasingly, disease-associated glial states are emerging therapeutic targets [161]. Astrocyte loss, for example, is being addressed by transplantation of GDNF-secreting astrocyte progenitor cells into the spinal cord and motor cortex (NCT02943850, NCT05306457) [162]. Microglial and mast-cell activation is being targeted by masitinib (NCT07174492), a tyrosine kinase inhibitor [163]. However, immunomodulatory strategies to limit neurotoxic glial activity by preserving OD myelinating capacity, a mutation agnostic approach relevant to the majority of ALS patients, are unexplored clinically and may represent a therapeutic opportunity [164].

ALS-increased DEGs overlapped significantly with neuron marker genes (e.g., *CDKL2*, *SCN7A*, *AKR1C2*; Figure 7C,F), and inspection of more granular markers indicated that mRNAs expressed by GABAergic interneurons were frequently increased in the ALS cortex (Figure 7K). For example, expression of calbindin 1 (*CALB1*), which encodes an interneuron marker protein [165], was among the ALS-increased DEGs identified in this study (SMD = 0.37, *p* = 4.6 × 10^−04^, FDR = 0.021). Interneurons suppress electrical activity and normally comprise 20–30% of all cortical neurons [166]. Their activity in the ALS cortex may combat neuronal hyperexcitability resulting from an imbalance between excitatory (glutamate) and suppressive (GABA) neurotransmitters [156,167,168]. Such hyperexcitability of upper motor neurons early in the disease may initiate top-down cascades leading to lower motor neuron dysfunction, with clinical manifestations such as hyperreflexia, fasciculation and spasticity [167]. Loss of interneurons can drive cortical disinhibition to amplify this process [169], and increasing interneuron activity has slowed disease progression in the SOD1-G93A ALS mouse model [170]. Interestingly, autopsy studies of ALS patients have revealed interneuron damage within the primary motor cortex [171], while other studies have described loss of interneuron subtypes within the deep L5/L6 cortical layers [172]. On the other hand, immunohistochemical studies show preservation of interneuron density in the upper cortical layers as well as maintenance of interneuron size throughout all layers [172]. Increased abundance of interneuron-associated mRNAs in the ALS cortex may therefore reflect expansion and/or activation of certain interneuron phenotypes [173], such as those that express calbindin-D28K [174,175], which may itself demarcate only a heterogeneous set of interneuron subtypes [176]. Cortical and spinal motor neuron hyperexcitability is also a potential target in ALS drug development [177]. For example, Kv7/KCNQ potassium-channel activator ezogabine reduced motor neuron excitability in a randomized phase 2 study (NCT02450552) [178], leading to further development of novel Kv7.2/7.3 channel openers (e.g., QRL-101, NCT06714396).

Gene expression changes in ALS cortex overlapped significantly with those previously identified from ALS spinal cord (Figure 6 and Figure S49), although expression shifts in the cortex were associated with smaller effect sizes [66]. The current meta-analysis identified 542 DEGs (269 ALS-increased, 273 ALS-decreased), but applying the same FDR and effect size thresholds would have identified 3.9 times this number (2131 DEGs) in the ALS spinal cord [66]. In the current study, there were only three ALS-increased DEGs (*EPHA2*, *ACSM4*, *C1orf210*) for which the meta-SMD estimate was “strong” (SMD ≥ 0.80), and only one ALS-decreased DEG (*SMIM41*) for which the meta-SMD estimate was “strong” (SMD ≤ −0.80). In contrast, there were 213 DEGs from the prior spinal cord meta-analysis [66] for which effect size estimates were within the “strong” range. We propose four possible explanations (not mutually exclusive). First, post-mortem transcriptome studies of the ALS cortex may be more heterogeneous compared to those from spinal cord, leading to weaker meta-SMD estimates. Second, spinal cord motor neurons may have increased susceptibility to multiple stressors, such as ER stress, hyperexcitability and calcium dysregulation [179], augmenting severity of post-mortem shifts in gene expression. Third, end-stage ALS pathology in the cortex may manifest primarily at the level of protein abundance, malformation and dysfunction [180], rather than changes in mRNA abundance. A previous proteomics study, for example, identified a larger number of proteins with altered abundance in the ALS cortex as compared to spinal cord [181]. Fourth, many gene expression changes in ALS spinal cord may be driven by white matter pathology [66]. This would magnify transcriptome shifts in the ALS spinal cord, consisting of approximately 80% white matter [182], with less impact on expression shifts in the brain (<50% white matter) [183].

Finally, we note several limitations of this study. First, this study evaluated post-mortem tissues, which can only provide insight into end-stage disease and may be associated with patient-specific variables that are difficult to fully control for in statistical analyses (e.g., genetic structure, disease duration, prior treatment history and comorbid conditions) [184]. Second, although our study included all studies for which primary datasets were available, the overall sample size of 16 studies provides only a limited basis for identifying associations related to moderator variables (e.g., cortex region, profiling technology and PMI). Third, our meta-analysis methodology treats effect size estimates from each of the 16 datasets as statistically independent. We found no evidence to contradict this assumption, but nonetheless, we cannot fully exclude sample overlap between studies (e.g., if two studies had analyzed the same most-mortem sample but assigned different identifiers). Fourth, we have drawn inferences regarding disease-related cell types based upon gene expression changes linked to cell type-specific marker genes. This approach has proof-of-concept in prior work [61,62,63], although such changes do not necessarily reflect shifts in cellular abundance in bulk or pseudobulk tissues samples, but may additionally arise from intracellular up= or down-regulation of specific genes. We have attempted to balance these considerations in our interpretation of findings (Figure 9).

## 5. Conclusions

The depth and scale of genomic datasets from post-mortem ALS tissues will continue to expand in the coming years and decades [185,186]. As every post-mortem sample represents a gift from a donor to the scientific community, an ongoing task will be to draw connections between existing and emerging data resources, which may indeed be the most important pathway towards progress in clinical transcriptomic research [187]. This study illustrates a meta-analytic approach for continued aggregation of evidence from such datasets. Although one-third of genes evaluated exhibit significant effect size heterogeneity (FDR < 0.05), we could identify 269 ALS-increased DEGs (FDR < 0.05 with SMD ≥ 0.20) along with 273 ALS-decreased DEGs (FDR < 0.05 with SMD ≤ −0.20). Our functional analysis of DEGs supports a model of end-stage disease that involves dysregulated mRNA processing together with disruption of specific cell populations (interneurons, microglia, endothelial cells, astrocytes, OD, OPC) and laminar layers (L1, L5, L6) (Figure 9). These results sharpen our focus on repeatable findings in ALS transcriptomic research and can inform basic laboratory research and future translation-oriented clinical studies. We anticipate that our findings can be updated as new data resources become available, with the goal of generating robust data-driven hypotheses to support basic and translational work needed for therapy development.

## Figures and Tables

**Table 1:** Meta-analysis gene expression studies. Each study evaluated post-mortem cortex samples from ALS patients and neurologically normal controls. Further details for each dataset are provided in Section 2.2, Section 2.3, Section 2.4, Section 2.5, Section 2.6, Section 2.7, Section 2.8, Section 2.9, Section 2.10, Section 2.11, Section 2.12, Section 2.13, Section 2.14, Section 2.15, Section 2.16 and Section 2.17 and footnotes (see below). Datasets are referred to by letter (column 1) throughout the manuscript.

Dataset	Accession ^a^	Release ^b^	*n* (ALS) ^c^	*n* (CTL) ^d^	Gene Count ^e^
A ^1, ‡, #, ‡‡, ¶¶, 6^	GSE4595	26 March 2007	11 (11)	9 (9)	13,327
B ^1, ‡, #, §§, ##, 4^	E-MTAB-1925	28 October 2013	3 (3)	3 (3)	12,379
C ^1, ‡, #, ‡‡, ##, 6^	E-MTAB-2325	26 February 2015	31 (31)	10 (10)	17,207
D ^2, §, #, ††, ##, 4^	GSE67196	19 July 2015	18 (18)	9 (9)	14,210
E ^2, ‡, #, ‡‡, ¶¶, 5^	GSE122649	20 September 2019	26 (13)	12 (6)	16,057
F ^2, †, #, ††, ¶¶, 5^	GSE137810	20 September 2019	675 (312)	137 (74)	15,807
G ^2, ‡, #, ‡‡, ##, 5^	EGAS00001004286	3 April 2020	11 (11)	8 (8)	15,302
H ^3, †, #, ††, ¶¶, 5^	GSE174332	9 July 2021	66 (33)	32 (16)	14,678
I ^3, §, ¶, §§, ##, 5^	syn45351388	28 November 2022	36 (16)	24 (12)	14,721
J ^3, §,^ *^, ††, ##, 5^	PRJNA918304	10 January 2023	17 (17)	6 (6)	15,197
K ^3, †, ¶, §§, ¶¶, 5^	GSE219280	17 July 2023	12 (6)	12 (6)	14,798
L ^2, ‡, ¶, ‡‡, ##, 6^	None	21 December 2023	98 (98)	38 (38)	11,771
M ^2, §, ¶, ‡‡, ##, 6^	EGAS00001007318	5 June 2024	38 (38)	28 (28)	15,491
N ^3, ‡,^ *^, ‡‡,^ **^, 5^	GSE271156	19 July 2024	4 (4)	2 (2)	15,339
O ^2, †, ¶, ‡‡, ¶¶, 5^	NG00178	25 April 2025	66 (22)	33 (11)	14,601
P ^3, ‡,^ *^, ‡‡, ¶¶, 4^	JGAS000852	11 November 2025	6 (6)	6 (6)	14,970

^a^ Dataset accession identifier (when available). ^b^ Date on which data were made available via database (or publication). ^c^ Number of ALS patient samples (parentheses: number of unique patients). ^d^ Number of CTL patient samples (parentheses: number of unique patients). ^e^ Number of protein-coding genes with detectable expression for which effect size was estimated. ^1^ Microarray. ^2^ Bulk RNA-seq. ^3^ snRNA-seq. ^‡^ All samples from motor cortex. ^†^ Some samples from motor cortex (with other cortical regions). ^§^ All samples from non-motor cortex. ^#^ Average post-mortem interval (PMI) < 15 h. ^¶^ Average PMI ≥ 15 h. * Average PMI unknown. ^‡‡^ ALS patients had sporadic disease. ^††^ ALS patients had sporadic or familial disease. ^§§^ ALS patients had familial disease (*C9ORF72*). ^##^ Average ALS patient age < 65 years. ^¶¶^ Average ALS patient ≥ 65 years. ** Average ALS patient age unknown. ^4^ ALS patients < 40% male. ^5^ ALS patients 40-60% male. ^6^ ALS patients > 60% male.

## Data Availability

The gene expression data analyzed in this study are available from public repositories (see Table 1). Dataset L (KCL BrainBank) is available from primary study investigators on reasonable request.

## Supplementary Materials

The following supporting information can be downloaded at https://www.mdpi.com/article/10.3390/brainsci16101120/s1, Figure S1: Expression ratio distributions (GSE4595); Figure S2: Microarray pseudoimages (GSE4595); Figure S3: Covariate variables: sex and age at death (GSE4595); Figure S4: Microarray pseudoimages and probe-level metrics (E-MTAB-1925); Figure S5: Expression signal distributions (E-MTAB-2325); Figure S6: Read mapping quality control metrics (GSE67196); Figure S7: Covariate variables: sex and age at death (GSE67196); Figure S8: Read mapping quality control metrics (GSE122649); Figure S9: Covariate variables: sex and age at death (GSE122649); Figure S10: Read mapping quality control metrics (GSE137810); Figure S11: Gene expression heatmap (GSE137810); Figure S12: Principal component (PC) plots (biologic factors; GSE137810); Figure S13: Principal component (PC) plots (technical factors; GSE137810); Figure S14: Model selection (GSE137810); Figure S15: Read mapping quality control metrics (EGAS00001004286); Figure S16: Read mapping quality control metrics (GSE174332); Figure S17: Distribution of counts, features, and mitochondrial/ribosomal reads among cells (GSE174332); Figure S18: Covariate variables: sex, age at death and cortical region (GSE174332); Figure S19: Read mapping quality control metrics (syn45351388); Figure S20: Distribution of counts, features, and mitochondrial/ribosomal reads among cells (syn45351388); Figure S21: Covariate variables: sex, age at death and cortical region (syn45351388); Figure S22: Read mapping quality control metrics and distribution of counts, features, and mitochondrial/ribosomal reads among cells (PRJNA918304); Figure S23: Covariate variables: sex and age at death (PRJNA918304); Figure S24: Read mapping quality control metrics and distribution of counts, features, and mitochondrial/ribosomal reads among cells (GSE219280); Figure S25: Covariate variables: sex, age at death and cortical region (GSE219280); Figure S26: Covariate variable: RIN (KCL BrainBank); Figure S27: Read mapping quality control metrics (EGAS00001007318); Figure S28: Covariate variables: sex, age at death and RIN (EGAS00001007318); Figure S29: Read mapping quality control metrics and distribution of counts, features, and mitochondrial/ribosomal reads among cells (GSE271156); Figure S30: Read mapping quality control metrics (NG00178); Figure S31: Covariate variables: sex, age at death and cortex region (NG00178); Figure S32: Distribution of counts, features, and mitochondrial/ribosomal reads among cells (JGAS000852); Figure S33: Covariate variables: sex and age at death (JGAS000852); Figure S34: Principal component (PC) plots (ALS vs. CTL samples); Figure S35: Normal-probability contours (ALS vs. CTL samples); Figure S36: Raw p-value empirical cumulative distribution functions (ECDFs); Figure S37: Raw p-value distributions; Figure S38: Volcano plots; Figure S39: Comparison of SMD estimates by estimation method (Datasets E, F, H, I, K, O); Figure S40: MA plots. SMD estimates (vertical axis) are plotted with respect to average normalized expression (horizontal axis); Figure S41: Gene length and differential expression; Figure S42: GC content and differential expression; Figure S43: Gene length and differential expression (alternative normalization); Figure S44: GC content and differential expression (alternative normalization); Figure S45: Heterogeneous genes; Figure S46: Meta-analysis differential expression analysis; Figure S47: Meta-analysis differential expression analysis (alternative normalization); Figure S48: SMD versus gene length (kb) and GC content (%) scatterplots; Figure S49: Comparison to DEGs from LCM-dissected spinal cord motor neuron meta-analysis [67]; Figure S50: Comparison to DEGs from whole blood [121]; Figure S51: Overlap between DEGs and ALS-associated genes (NHGRI-EBI GWAS catalog); Figure S52: Transcription factor (TF) binding sites located near DEGs; Table S1: ALS-increased DEGs and enrichment analyses (Excel file); Table S2: ALS-decreased DEGs and enrichment analyses (Excel file).

## Abbreviations

The following abbreviations are used in this manuscript:

ACC	Anterior cingulate cortex
AG	Angular gyrus
AST	Astrocyte
BP	Biological process
CC	Cell component
CQN	Conditional quantile normalization
CTL	Control
DEG	Differentially expressed gene
DLPFC	Dorsolateral prefrontal cortex
DSigDB	Drug signatures database
ECDF	Empirical cumulative distribution function
FDR	False discovery rate
FPKM	Fragments per kilobase of transcript per million mapped reads
FPPFC	Frontopolar prefrontal cortex
GO	Gene ontology
GWA	Genome-wide association
ITG	Inferior temporal gyrus
KEGG	Kyoto Encyclopedia of Genes and Genomes
L1	Molecular layer
L2	External granular layer
L3	External pyramidal layer
L4	Internal granular layer
L5	Internal pyramidal layer
L6	Multiform layer
M1	Primary motor cortex
MESH	Medical Subject Headings
MF	Molecular function
MSigDB	Molecular signature database
NN	Nearest neighbor
NUSE	Normalized unscaled standard error
NYGC	New York Genome Center
OD	Oligodendrocyte
OPC	Oligodendrocyte precursor cell
PC	Principal component
PMI	Post-mortem interval
PoCG	Postcentral gyrus
PWM	Position weight matrix
RIN	RNA integrity number
RLE	Relative log expression
RMA	Robust multichip average
S1	Primary somatosensory cortex
S1E	Primary somatosensory cortex of the eardrum
SCAN	Single-channel array normalization
SMD	Standardized mean difference
SMG	Supramarginal gyrus
SOM	Self-organizing map
SPL	Superior parietal lobule
STG	Superior temporal gyrus
TF	Transcription factor
TSS	Transcription start site
V1	Primary visual cortex
VLPFC	Ventrolateral prefrontal cortex
VST	Variance-stabilizing transformation
WM	White matter
