# Supplementary material for: Meta-Analysis of ALS Post-Mortem Cerebral Cortex Transcriptome Studies Identifies Robust Shifts in Gene Expression Despite Significant Between-Study Heterogeneity
Source: Brain Sci. 2026 Oct 16;16(10):1120. doi: 10.3390/brainsci16101120 (PMC13702254; doi:10.3390/brainsci16101120)
Supplement: Supplementary file 1 [file brainsci-16-01120-s001.zip › Supplemental Files/Supplementary figures.pdf]

<u>Supplemental Figures</u>

Meta-analysis of ALS post-mortem cerebral cortex transcriptome studies identifies robust shifts in gene expression despite significant between-study heterogeneity

William R. Swindell[1,†], Aminah Ali[2], Alfredo Iacoangeli[3,4,5,6], Salman Bhai[7]
[1]Department of Internal Medicine, University of Texas Southwestern Medical Center, Dallas, TX, USA
[2]Department of Biostatistics & Health Informatics, Institute of Psychiatry Psychology & Neuroscience, King's College London, 16 De Crespigny Park, London, SE5 8AB, UK.
[3]Department of Biostatistics and Health Informatics, Institute of Psychiatry, Psychology and Neuroscience, King's College London, London, UK.
[4]Department of Basic and Clinical Neuroscience, Maurice Wohl Clinical Neuroscience Institute, Institute of Psychiatry, Psychology and Neuroscience, King's College London, London, UK.
[5]NIHR Maudsley Biomedical Research Centre (BRC), South London and Maudsley NHS Foundation Trust and King's College London, London, UK.
[6]Perron Institute for Neurological and Translational Science, Ground RR Block QE II Medical Centre Ralph & Patricia Sarich Neuroscience Building, 8 Verdun St, Nedlands, WA 6009, Australia.
[7]Department of Neurology, University of Texas Southwestern Medical Center, Dallas, TX, USA
[†]Corresponding author

<u>Correspondence</u>:
Dr. William R. Swindell, D.O., Ph.D.
Assistant Professor
University of Texas Southwestern Medical Center
Department of Internal Medicine
Division of Hospital Medicine
Dallas, Texas, 75390-9175
Email: William.Swindell@UTSouthwestern.edu
Phone: 214-645-3597
Fax: 214-645-6757

Figure S1. Expression ratio distributions (GSE4595). (A) Raw ratio distributions (no normalization). (B) Ratio distributions following global median location correction (*). (C) Ratio distributions after global median location correction and quantile normalization (**). (D) Ratio distributions following global median location correction and quantile normalization with loess-correction of spatial artifacts (***). In (A) - (D), boxes outline the middle 50% of ratios among probes for each array sample (middle line: median; whiskers: 10th to 90th percentiles).

Figure S2. Microarray pseudoimages (GSE4595). (A) Pseudoimages before spatial correction. (B) Pseudoimages after spatial correction. In (A) and (B), raw expression ratios (cy5 /cy3) were normalized by global median location correction, followed by quantile normalization, with z-score normalization as a final step (see scale). In (B), an additional loess spatial correction was applied after z-score normalization. Sample labels are shown (bottom margin). The value in parentheses is the pseudo R^2, which was calculated from the correlation between observed expression ratios and loess-fitted values, such that higher pseudo R^2 values indicate a stronger spatial bias pattern.

Figure S3. Covariate variables: sex and age at death (GSE4595). (A, D) Samples are plotted with respect to the first two PC axes. Points are color-coded as indicated by each legend (top margin). (B, E) Ellipses are drawn around sample subsets as indicated by each legend (top margin). Each ellipse outlines the 95% normal-probability contour for each sample subset. The percent overlap relative to the combined ellipse area is indicated. (C) Percentage of males by disease status. (F) Average age at death by disease status. In (C) and (F), the percentage or average value in each group is shown (± 1 standard error) and the test for group difference is indicated (top margin).

Figure S4. Microarray pseudoimages and probe-level metrics (E-MTAB-1925). (A) Microarray pseudoimages. (B) Normalized unscaled standard error (NUSE). (C) Relative log expression (RLE). In (B) and (C), boxes outline the middle 50% of NUSE or RLE values among probes on each array.

Figure S5. Expression signal distributions (E-MTAB-2325). (A, D) Signal distributions prior to normalization. (B, E) Signal distributions after background correction. (C, F) Signal distributions after background correction and quantile normalization. In (A) – (F), boxes outline the middle 50% of signals for each array sample (middle line: median; whiskers: 10th to 90th percentiles).

Figure S6. Read mapping quality control metrics (GSE67196). (A) Pre-filter read count. (B) Post-filter read count. (C) Percentage of mapped reads. (D) Percentage of uniquely mapped reads. (E) Percentage of intragenic reads. (F) Percentage of exonic reads. (G) Percentage of ribosomal reads. (H) Percentage of genes with detectable expression (count > 0 and FPKM ≥ 0.3).

Figure S7. Covariate variables: sex and age at death (GSE67196). (A, D) Samples are plotted with respect to the first two PC axes. Points are color-coded as indicated by each legend (top margin). (B, E) Ellipses are drawn around sample subsets as indicated by each legend (top margin). Each ellipse outlines the 95% normal-probability contour for each sample subset. The

percent overlap relative to the combined ellipse area is indicated. (C) Percentage of males by disease status. (F) Average age at death by disease status. In (C) and (F), the percentage or average value in each group is shown (± 1 standard error) and the test for group difference is indicated (top margin).

Figure S8. Read mapping quality control metrics (GSE122649). (A) Pre-filter read count. (B) Post-filter read count. (C) Percentage of mapped reads. (D) Percentage of uniquely mapped reads. (E) Percentage of intragenic reads. (F) Percentage of exonic reads. (G) Percentage of ribosomal reads. (H) Percentage of genes with detectable expression (count > 0 and FPKM ≥ 0.3).

Figure S9. Covariate variables: sex and age at death (GSE122649). (A, D) Samples are plotted with respect to the first two PC axes. Points are color-coded as indicated by each legend (top margin). (B, E) Ellipses are drawn around sample subsets as indicated by each legend (top margin). Each ellipse outlines the 95% normal-probability contour for each sample subset. The percent overlap relative to the combined ellipse area is indicated. (C) Percentage of males by disease status. (F) Average age at death by disease status. In (C) and (F), the percentage or average value in each group is shown (± 1 standard error) and the test for group difference is indicated (top margin).

Figure S10. Read mapping quality control metrics (GSE137810). Results are shown for 911 sequencing runs each associated with a unique SRR identifier. (A) Pre-filter read count. (B) Post-filter read count. (C) Percentage of mapped reads. (D) Percentage of uniquely mapped reads. (E) Percentage of intragenic reads. (F) Percentage of exonic reads. (G) Percentage of ribosomal reads. (H) Percentage of genes with detectable expression (count > 0 and FPKM ≥ 0.3). In (A) - (H), box plots (top panel) outline the middle 50% of runs (whiskers: 25th to 75th percentiles; round symbols: > 90th percentile or < 10th percentile). Histograms (bottom panel) show the distribution among all runs (dark grey background: middle 50% of runs; light grey background: middle 80% of runs). The median value is shown within each figure.

Figure S11. Gene expression heatmap (GSE137810). The yellow-blue heatmap (right) shows expression of 15954 protein-coding genes (rows) across 812 samples (columns). Expression of each gene was normalized by subtracting the mean value across all samples. Genes were clustered using hierarchical clustering with average linkage and the Euclidian distance metric (see dendrogram, left). Sample characteristics are indicated by color-coded categories (see legends, bottom).

Figure S12. Principal component (PC) plots (biologic factors; GSE137810). Results are shown for 812 unique samples. (A, C, E) Samples are plotted with respect to the first two PC axes. Points are color-coded as indicated by each legend (top margin). (B, D, F) Ellipses are drawn around sample subsets as indicated by each legend (top margin). Each ellipse outlines the 95% normal-probability contour for each sample subset.

Figure S13. Principal component (PC) plots (technical factors; GSE137810). Results are shown for 812 unique samples. (A, C, E, G, I, K) Samples are plotted with respect to the first two PC axes. Points are color-coded as indicated by each legend (top margin). (B, D, F, H, J, L)

Ellipses are drawn around sample subsets as indicated by each legend (top margin). The 95% normal-probability contour is drawn for each subset.

Figure S14. Model selection (GSE137810). (A) Random forest variable importance. Samples were clustered into 20 non-overlapping groups (hierarchical clustering with average linkage). Random forest was then used to predict the cluster assignment of each sample utilizing the variables listed (left margin). The percentage increase in classification error rate associated with omission of each variable is shown. (B) Likelihood ratio test (LRT) p-values. LR testing was used to compare goodness-of-fit between a univariate base (expression = subject) and bivariate full model (expression = subject + factor). The LRT was performed for each factor listed (left margin). Linear mixed-effect models were fit using maximum likelihood estimation. A higher -Log_{10}(P-value) supports increased variable importance with respect to improved goodness-of-fit (decreased model deviance). In (A) and (B), red font corresponds to biological factors, whereas black font corresponds to technical factors. (C) Model screen. All possible models with 6-8 variables were screened. Models included a core set of 5 base variables (subject, ALS, age, sex, region) with 1-3 added technical variables. An LRT was performed in each case to compare the base (5 variables only) and full models (6-8 variables). The top 10 full models are shown. In (B) and (C), LRT testing was performed using a subset of representative genes, deterministically selected by choosing a representative gene from each cluster (identified by hierarchical cluster analysis). Boxplots outline the middle 50% of -Log_{10}(P-value) estimates for each model (whiskers: 10th to 90th percentiles). The median -Log_{10}(P-value) among representative genes is shown for each model (right margin).

Figure S15. Read mapping quality control metrics (EGAS00001004286). (A) Pre-filter read count. (B) Post-filter read count. (C) Percentage of mapped reads. (D) Percentage of uniquely mapped reads. (E) Percentage of intragenic reads. (F) Percentage of exonic reads. (G) Percentage of ribosomal reads. (H) Percentage of genes with detectable expression (count > 0 and FPKM $\geq$ 0.3).

Figure S16. Read mapping quality control metrics (GSE174332). (A) Read count. (B) Number of cells per sample. (C) Reads per cell. (D) Sequencing saturation. (E) Percentage of RNA read bases with Phred quality score $\geq$ 30. (F) Percentage of reads in cells. (G) Percentage of mapped reads. (H) Percentage of protein-coding genes having detectable expression (count > 0 with FPKM > 0.05). In (A) - (H), box plots (top panel) outline the middle 50% of samples (whiskers: 25th to 75th percentiles; round symbols: > 90th percentile or < 10th percentile). Histograms (bottom panel) show the distribution among all samples (dark grey background: middle 50% of samples; light grey background: middle 80% of samples). The median value is shown within each figure.

Figure S17. Distribution of counts, features, and mitochondrial/ribosomal reads among cells (GSE174332). (A) Number of RNA molecules among cells. (B) Number of genes detected among cells. (C) Percentage of mitochondrial reads. (D) Percentage of ribosomal reads. For each sample, boxes outline the middle 50% of values among cells (whiskers: 10th to 90th percentiles).

Figure S18. Covariate variables: sex, age at death and cortical region (GSE174332). (A, D, G) Samples are plotted with respect to the first two PC axes. Points are color-coded as indicated

by each legend (top margin). (B, E, H) Ellipses are drawn around sample subsets as indicated by each legend (top margin). Each ellipse outlines the 95% normal-probability contour for each sample subset. The percent overlap relative to the combined ellipse area is indicated (upper left). (C) Percentage of males by disease status. (F) Average age at death by disease status. (I) Percent motor cortex (MCX) by disease status. In (C), (F) and (I), the percentage or average value in each group is shown (± 1 standard error) and the test for group difference is indicated (top margin). In part (I), a generalized linear mixed model (GLMM) was used to test for any group difference (with subject as a random effect).

Figure S19. Read mapping quality control metrics (syn45351388). (A) Read count. (B) Number of cells per sample. (C) Reads per cell. (D) Sequencing saturation. (E) Percentage of RNA read bases with Phred quality score ≥ 30. (F) Percentage of reads in cells. (G) Percentage of mapped reads. (H) Percentage of protein-coding genes having detectable expression (count > 0 with FPKM > 0.05). In (A) - (H), box plots (top panel) outline the middle 50% of samples (whiskers: 25th to 75th percentiles; round symbols: > 90th percentile or < 10th percentile). Histograms (bottom panel) show the distribution among all samples (dark grey background: middle 50% of samples; light grey background: middle 80% of samples). The median value is shown within each figure.

Figure S20. Distribution of counts, features, and mitochondrial/ribosomal reads among cells (syn45351388). (A) Number of RNA molecules among cells. (B) Number of genes detected among cells. (C) Percentage of mitochondrial reads. (D) Percentage of ribosomal reads. For each sample, boxes outline the middle 50% of values among cells (whiskers: 10th to 90th percentiles).

Figure S21. Covariate variables: sex, age at death and cortical region (syn45351388). (A, D, G) Samples are plotted with respect to the first two PC axes. Points are color-coded as indicated by each legend (top margin). (B, E, H) Ellipses are drawn around sample subsets as indicated by each legend (top margin). Each ellipse outlines the 95% normal-probability contour for each sample subset. The percent overlap relative to the combined ellipse area is indicated (bottom left). (C) Percentage of males by disease status. (F) Average age at death by disease status. (I) Percent frontal cortex (FCX) by disease status. In (C), (F) and (I), the percentage or average value in each group is shown (± 1 standard error) and the test for group difference is indicated (top margin). In part (I), a generalized linear mixed model (GLMM) was used to test for any group difference (with subject as a random effect).

Figure S22. Read mapping quality control metrics and distribution of counts, features, and mitochondrial/ribosomal reads among cells (PRJNA918304). (A) Read count. (B) Number of cells per sample. (C) Reads per cell. (D) Sequencing saturation. (E) Percentage of RNA read bases with Phred quality score ≥ 30. (F) Percentage of reads in cells. (G) Percentage of mapped reads. (H) Percentage of protein-coding genes having detectable expression (count > 0 with FPKM > 0.05). In (A) - (H), box plots (top panel) outline the middle 50% of samples (whiskers: 25th to 75th percentiles; round symbols: > 90th percentile or < 10th percentile). Histograms (bottom panel) show the distribution among all samples (dark grey background: middle 50% of samples; light grey background: middle 80% of samples). The median value is shown within each figure. (I) Number of RNA molecules among cells. (J) Number of genes detected among

cells. (K) Percentage of mitochondrial reads. (L) Percentage of ribosomal reads. For each sample, boxes outline the middle 50% of values among cells (whiskers: 10th to 90th percentiles).

Figure S23. Covariate variables: sex and age at death (PRJNA918304). (A, D) Samples are plotted with respect to the first two PC axes. Points are color-coded as indicated by each legend (top margin). (B, E) Ellipses are drawn around sample subsets as indicated by each legend (top margin). Each ellipse outlines the 95% normal-probability contour for each sample subset. The percent overlap relative to the combined ellipse area is indicated. (C) Percentage of males by disease status. (F) Average age at death by disease status. In (C) and (F), the percentage or average value in each group is shown (± 1 standard error) and the test for group difference is indicated (top margin).

Figure S24. Read mapping quality control metrics and distribution of counts, features, and mitochondrial/ribosomal reads among cells (GSE219280). (A) Read count. (B) Number of cells per sample. (C) Reads per cell. (D) Sequencing saturation. (E) Percentage of RNA read bases with Phred quality score ≥ 30. (F) Percentage of reads in cells. (G) Percentage of mapped reads. (H) Percentage of protein-coding genes having detectable expression (count > 0 with FPKM > 0.05). In (A) - (H), box plots (top panel) outline the middle 50% of samples (whiskers: 25th to 75th percentiles; round symbols: > 90th percentile or < 10th percentile). Histograms (bottom panel) show the distribution among all samples (dark grey background: middle 50% of samples; light grey background: middle 80% of samples). The median value is shown within each figure. (I) Number of RNA molecules among cells. (J) Number of genes detected among cells. (K) Percentage of mitochondrial reads. (L) Percentage of ribosomal reads. For each sample, boxes outline the middle 50% of values among cells (whiskers: 10th to 90th percentiles).

Figure S25. Covariate variables: sex, age at death and cortical region (GSE219280). (A, D, G) Samples are plotted with respect to the first two PC axes. Points are color-coded as indicated by each legend (top margin). (B, E, H) Ellipses are drawn around sample subsets as indicated by each legend (top margin). Each ellipse outlines the 95% normal-probability contour for each sample subset. The percent overlap relative to the combined ellipse area is indicated. (C) Percentage of males by disease status. (F) Average age at death by disease status. (I) Percent motor cortex (MCX) by disease status. In (C), (F) and (I), the percentage or average value in each group is shown (± 1 standard error) and the test for group difference is indicated (top margin). In part (I), a generalized linear mixed model (GLMM) was used to test for any group difference (with subject as a random effect).

Figure S26. Covariate variable: RIN (KCL BrainBank). (A) Samples are plotted with respect to the first two PC axes. Points are color-coded as indicated by the legend (top margin). (B) Ellipses are drawn around sample subsets as indicated by each legend (top margin). Each ellipse outlines the 95% normal-probability contour for each sample subset. The percent overlap relative to the combined ellipse area is indicated. (C) Average RIN by disease status (all samples). (D) Average RIN by disease status (samples with RIN ≥ 5). In (C) and (D), the average value in each group is shown (± 1 standard error) and the test for group difference is indicated (two-sample two-tailed t-test; top margin).

Figure S27. Read mapping quality control metrics (EGAS00001007318). (A) Pre-filter read count. (B) Post-filter read count. (C) Percentage of mapped reads. (D) Percentage of uniquely mapped reads. (E) Percentage of intragenic reads. (F) Percentage of exonic reads. (G) Percentage of ribosomal reads. (H) Percentage of genes with detectable expression (count > 0 and FPKM ≥ 0.3).

Figure S28. Covariate variables: sex, age at death and RIN (EGAS00001007318). (A, D, G) Samples are plotted with respect to the first two PC axes. Points are color-coded as indicated by each legend (top margin). (B, E, H) Ellipses are drawn around sample subsets as indicated by each legend (top margin). Each ellipse outlines the 95% normal-probability contour for each sample subset. The percent overlap relative to the combined ellipse area is indicated. (C) Percentage of males by disease status. (F) Average age at death by disease status. (I) Average RIN by disease status. In (C), (F) and (I), the percentage or average value in each group is shown (± 1 standard error) and the test for group difference is indicated (top margin).

Figure S29. Read mapping quality control metrics and distribution of counts, features, and mitochondrial/ribosomal reads among cells (GSE271156). (A) Read count. (B) Number of cells per sample. (C) Reads per cell. (D) Sequencing saturation. (E) Percentage of RNA read bases with Phred quality score ≥ 30. (F) Percentage of reads in cells. (G) Percentage of mapped reads. (H) Percentage of protein-coding genes having detectable expression (count > 0 with FPKM > 0.05). In (A) - (H), box plots (top panel) outline the middle 50% of samples (whiskers: 25th to 75th percentiles; round symbols: > 90th percentile or < 10th percentile). Histograms (bottom panel) show the distribution among all samples (dark grey background: middle 50% of samples; light grey background: middle 80% of samples). The median value is shown within each figure. (I) Number of RNA molecules among cells. (J) Number of genes detected among cells. (K) Percentage of mitochondrial reads. (L) Percentage of ribosomal reads. For each sample, boxes outline the middle 50% of values among cells (whiskers: 10th to 90th percentiles).

Figure S30. Read mapping quality control metrics (NG00178). Results are shown for 911 sequencing runs each associated with a unique SRR identifier. (A) Pre-filter read count. (B) Post-filter read count. (C) Percentage of mapped reads. (D) Percentage of uniquely mapped reads. (E) Percentage of intragenic reads. (F) Percentage of exonic reads. (G) Percentage of ribosomal reads. (H) Percentage of genes with detectable expression (count > 0 and FPKM ≥ 0.3). In (A) - (H), box plots (top panel) outline the middle 50% of runs (whiskers: 25th to 75th percentiles; round symbols: > 90th percentile or < 10th percentile). Histograms (bottom panel) show the distribution among all runs (dark grey background: middle 50% of runs; light grey background: middle 80% of runs). The median value is shown within each figure.

Figure S31. Covariate variables: sex, age at death and cortex region (NG00178). (A, D, G) Samples are plotted with respect to the first two PC axes. Points are color-coded as indicated by each legend (top margin; FC: frontal cortex, MC: motor cortex, OC: occipital cortex). (B, E, H) Ellipses are drawn around sample subsets as indicated by each legend (top margin). Each ellipse outlines the 95% normal-probability contour for each sample subset. The percent overlap relative to the combined ellipse area is indicated in (B) and (E). (C) Percentage of males by disease status. (F) Average age at death by disease status. In (C) and (F), the percentage or average value

in each group is shown (± 1 standard error) and the test for group difference is indicated (top margin).

Figure S32. Distribution of counts, features, and mitochondrial/ribosomal reads among cells (JGAS000852). (A) Number of RNA molecules among cells. (B) Number of genes detected among cells. (C) Percentage of mitochondrial reads. (D) Percentage of ribosomal reads. For each sample, boxes outline the middle 50% of values among cells (whiskers: 10th to 90th percentiles).

Figure S33. Covariate variables: sex and age at death (JGAS000852). (A, D) Samples are plotted with respect to the first two PC axes. Points are color-coded as indicated by each legend (top margin). (B, E) Ellipses are drawn around sample subsets as indicated by each legend (top margin). Each ellipse outlines the 95% normal-probability contour for each sample subset. The percent overlap relative to the combined ellipse area is indicated. (C) Percentage of males by disease status. (F) Average age at death by disease status. In (C) and (F), the percentage or average value in each group is shown (± 1 standard error) and the test for group difference is indicated (top margin).

Figure S34. Principal component (PC) plots (ALS vs. CTL samples). Samples are plotted with respect to the first two PC axes. The green dotted line is the linear discriminant function boundary distinguishing ALS and CTL samples. The weighted classification accuracy (%) is shown (green font).

Figure S35. Normal-probability contours (ALS vs. CTL samples). Samples are plotted with respect to the first two PC axes. Ellipses are drawn around sample subsets as indicated by each legend (top margin). Each ellipse outlines the 95% normal-probability contour for each sample subset. The percent overlap relative to the combined ellipse area is indicated (green font).

Figure S36. Raw p-value empirical cumulative distribution functions (ECDFs). Raw p-values were obtained using linear models based on coefficients estimated for terms delineating ALS and CTL samples. The raw p-value ECDF is shown for each dataset analyzed. The Kolmogorov-Smirnov test was used to test for non-uniformity of the p-value distribution (see test statistic and p-value, lower right).

Figure S37. Raw p-value distributions. Linear models were used to test for a significant difference in the expression of protein-coding genes between ALS and CTL samples. P-values were derived from coefficient estimates associated with the model term delineating ALS and CTL samples. The distribution of raw p-values is shown for each dataset analyzed.

Figure S38. Volcano plots. The $-\log_{10}$-transformed p-value (vertical axis) is plotted with respect to SMD estimates (horizontal axis) for each dataset. Each symbol represents an individual protein-coding gene. Symbols are color-coded based upon their significance using either nominal p-value or FDR thresholds (top margin).

Figure S39. Comparison of SMD estimates by estimation method (Datasets E, F, H, I, K, O). (A - F) Each figure compares SMD estimates obtained using non-singular mixed effects

models (left) and fixed effect models (right). The latter approach involved averaging samples by subject (see Methods) and was used only for genes in which the initially formulated mixed effect model generated a singular fit. The number of SMD estimates generated by each approach is indicated (bottom margin). Boxes outline the middle 50% of SMD estimates in each group (whiskers: 10th to 90th percentiles). The p-value generated by comparing SMD estimates from each group is indicated (top margin, Wilcoxon rank sum test). (G) SMD estimate comparison across datasets (Spearman rank correlation). SMD estimates from datasets E, H, I, K and O were each compared to those from the other 15 datasets. Correlation coefficients were calculated separately based on model type (see legend, top margin). A total of 15 correlation coefficients were calculated from each dataset and estimation method. Boxes outline the middle 50% of these correlations (whiskers: 10th to 90th percentiles). The analysis was not performed with respect to dataset F because too few SMD estimates (n = 8) had been generated using fixed effect models.

Figure S40. MA plots. SMD estimates (vertical axis) are plotted with respect to average normalized expression (horizontal axis). Each symbol represents an individual protein-coding gene. Symbols are color-coded based upon their significance using either nominal p-value or FDR thresholds (top margin). The non-parametric locally estimated scatterplot smoothing (LOESS) estimate is shown (green line). In part (A), it was not possible to quantify absolute gene abundance, since microarray data files provided only ratios between Cy3 and Cy5 labelled cRNA signal intensities. The horizontal axis in (A) therefore shows gene expression quantiles calculated from bulk RNA-seq and snRNA-seq experiments (datasets *D* - *P*).

Figure S41. Gene length and differential expression. Protein-coding genes were binned based on gene length and the percentage of increased (red) or decreased (blue) DEGs in each bin is shown (DEGs: $P < 0.05$ with SMD ≥ 0.20 or SMD ≤ 0.20). An asterisk indicates an overabundance of DEGs within any one bin ($P < 0.05$, Fisher's exact test). Yellow font is used to denote an overabundance of increased or decreased DEGs within any one bin ($P < 0.05$, Fisher's exact test).

Figure S42. GC content and differential expression. Protein-coding genes were binned based on GC content and the percentage of increased (red) or decreased (blue) DEGs in each bin is shown (DEGs: $P < 0.05$ with SMD ≥ 0.20 or SMD ≤ 0.20). An asterisk indicates an overabundance of DEGs within any one bin ($P < 0.05$, Fisher's exact test). Yellow font is used to denote an overabundance of increased or decreased DEGs within any one bin ($P < 0.05$, Fisher's exact test).

Figure S43. Gene length and differential expression (alternative normalization). The analysis was repeated using data normalized by robust multichip average (dataset *B*) or variance stabilizing transformation (datasets *D* – *P*). No change was made to the normalization approach used for datasets *A* and *C*. Protein-coding genes were binned based on gene length and the percentage of increased (red) or decreased (blue) DEGs in each bin is shown (DEGs: $P < 0.05$ with SMD ≥ 0.20 or SMD ≤ 0.20). An asterisk indicates an overabundance of DEGs within any one bin ($P < 0.05$, Fisher's exact test). Yellow font is used to denote an overabundance of increased or decreased DEGs within any one bin ($P < 0.05$, Fisher's exact test).

Figure S44. GC content and differential expression (alternative normalization). The analysis was repeated using data normalized by robust multichip average (dataset *B*) or variance stabilizing transformation (datasets *D* – *P*). No change was made to the normalization approach used for datasets *A* and *C*. Protein-coding genes were binned based on GC content and the percentage of increased (red) or decreased (blue) DEGs in each bin is shown (DEGs: $P < 0.05$ with $SMD \geq 0.20$ or $SMD \leq 0.20$). An asterisk indicates an overabundance of DEGs within any one bin ($P < 0.05$, Fisher's exact test). Yellow font is used to denote an overabundance of increased or decreased DEGs within any one bin ($P < 0.05$, Fisher's exact test).

Figure S45. Heterogeneous genes. (A) Genes with greatest between-study heterogeneity. The top-ranked 32 genes with lowest Cochran's Q statistic p-value are shown. Heatmaps are color-coded based on SMD estimates from each study (see legend). SMD meta-estimates are listed with p-values in parentheses (right margin). (B – E) Forest plots. SMD estimates are shown for each study with 95% confidence intervals. The size of point estimates (squares) is proportional to the total number of samples from each dataset (left margin parentheses). The meta-estimate is represented by a magenta diamond and dashed vertical line. The SMD estimate from each study is listed with 95% confidence interval (right margin). Cochran's Q statistic for heterogeneity is shown with associated p-value (top margin).

Figure S46. Meta-analysis differential expression analysis. (A) Raw p-value distribution. (B) Raw p-value empirical cumulative distribution function. The Kolmogorov-Smirnov test was used to test if the p-value distribution was non-uniform (see test statistic and p-value, lower right). (C) Volcano plot. The -log10-transformed p-value (vertical axis) is plotted with respect to the SMD meta-estimate (horizontal axis). Each symbol represents a protein-coding gene. Genes meeting differential expression criteria are color-coded (see top margin legend). (D) MA plot. The SMD meta-estimate (vertical axis) is plotted with respect to average expression quantile (horizontal axis). Each symbol represents a protein-coding gene with genes meeting differential expression criteria color-coded (see top margin legend). The non-parametric locally estimated scatterplot smoothing (LOESS) estimate is shown (green line). (E) Gene length and differential expression. (F) GC content and differential expression. In (E) and (F), protein-coding genes were binned based on gene length or GC content and the percentage of increased (red) or decreased (blue) DEGs in each bin is shown (DEGs: $FDR < 0.05$ with $SMD \geq 0.20$ or $SMD \leq -0.20$). An asterisk indicates an overabundance of DEGs within any one bin ($P < 0.05$, Fisher's exact test). Yellow font is used to denote an overabundance of increased or decreased DEGs within any one bin ($P < 0.05$, Fisher's exact test).

Figure S47. Meta-analysis differential expression analysis (alternative normalization). The analysis was repeated using data normalized by robust multichip average (dataset *B*) or variance stabilizing transformation (datasets *D* – *P*). No change was made to the normalization approach used for datasets *A* and *C*. (A) MA plot. The SMD meta-estimate (vertical axis) is plotted with respect to average expression quantile (horizontal axis). Each symbol represents a protein-coding gene with genes meeting differential expression criteria color-coded (see top margin legend). The non-parametric locally estimated scatterplot smoothing (LOESS) estimate is shown (green line). (B) Gene length and differential expression. (C) GC content and differential expression. In (B) and (C), protein-coding genes were binned based on gene length or GC content and the percentage of increased (red) or decreased (blue) DEGs in each bin is shown (DEGs: $FDR < 0.05$

with SMD ≥ 0.20 or SMD ≤ -0.20). An asterisk indicates an overabundance of DEGs within any one bin (P < 0.05, Fisher's exact test). Yellow font is used to denote an overabundance of increased or decreased DEGs within any one bin (P < 0.05, Fisher's exact test).

Figure S48. SMD versus gene length (kb) and GC content (%) scatterplots. (A) SMD vs. gene length. (B) SMD vs. GC content. (C) SMD vs. gene length (alternative normalization). (D) SMD vs. GC content (alternative normalization). In (A) – (D), each point represents an individual protein-coding gene. The linear regression fit is shown (straight yellow line). The yellow ellipse outlines the 90% of genes closest to the bivariate centroid (Mahalanobis distance). The Spearman correlation estimate and associated p-value are shown (upper right). Color-coded quadrants are defined based on SMD (SMD > 0 or SMD < 0) and GC content percentage (above median or below median). The percentage of genes within each quadrant is indicated (top margin). Top margin percentage values in red font denote quadrants containing a percentage of genes that significantly exceeds 25% (P < 0.05, one-sample proportion test with Yates' continuity correction). The magnitude of each percentage is graphically indicated (right sidebar). In parts (C) and (D), results are shown using meta-SMD estimates generated using an alternative normalization approach, in which data were normalized by robust multichip average (dataset *B*), variance stabilizing transformation (datasets *D* – *P*), or without any change in normalization (datasets *A* and *C*).

Figure S49. Comparison to DEGs from LCM-dissected spinal cord motor neurons meta-analysis (Swindell 2025, Frontiers in Genetics 15:1385114). (A, B) Venn diagrams. Overlap is shown between meta-analysis ALS DEGs from cortex (current study) and LCM-dissected spinal cord motor neurons (prior study). Significance of overlap was evaluated using Fisher's exact test (bottom margin). Both gene sets are defined based on the same thresholds (FDR < 0.05 with SMD ≥ 0.20 or SMD ≤ -0.20). (C, D) Cortex SMD estimates of LCM-dissected spinal cord motor neuron DEGs (blue: SMD < 0 in cortex; red: SMD > 0 in cortex). The percentage of genes with SMD > 0 in cortex (red) or SMD < 0 in cortex is shown (upper left). This percentage is also represented by the pie chart. The outer pie chart (lower right) shows the percentage of genes with SMD > 0 in cortex (red) and SMD < 0 in cortex (blue) among all genes from both meta-analyses. The p-value was obtained by comparing inner and outer pie chart frequencies (Fisher's exact test). (E) ALS-increased genes (cortex and LCM-dissected spinal cord motor neurons). (F) ALS-decreased genes (cortex and LCM-dissected spinal cord motor neurons). In (E) and (F), bars show SMD estimates in cortex and circles denote SMD estimates from spinal cord motor neurons. The size of circles is proportional to the p-value associated with the SMD estimate from the spinal cord meta-analysis (see scale, right margin). Genes shown in red font (bottom margin) were significantly increased in both meta-analysis studies (FDR < 0.05, SMD ≥ 0.20). Genes shown in blue font (bottom margin) were significantly decreased in both meta-analysis studies (FDR < 0.05, SMD ≤ -0.20).

Figure S50. Comparison to DEGs from whole blood (Swindell et al. 2019, J Transl Med 17:170). (A, B) Venn diagrams. Overlap is shown between meta-analysis ALS DEGs from cortex (current study) and whole blood (prior study). Significance of overlap was evaluated using Fisher's exact test (bottom margin). To remain consistent with the original publication, DEGs from cortex are defined based on different criteria (FDR < 0.05, SMD ≥ 0.20 or SMD ≤ -0.20) than those from whole blood (FDR < 0.10 with > 10% expression change). (C, D) Cortex SMD

estimates of whole blood DEGs (blue: SMD < 0 in cortex; red: SMD > 0 in cortex). The percentage of genes with SMD > 0 in cortex (red) or SMD < 0 in cortex is shown (upper left). This percentage is also represented by the pie chart. The outer pie chart (lower right) shows the percentage of genes with SMD > 0 in cortex (red) and SMD < 0 in cortex (blue) among all genes from both meta-analyses. The p-value was obtained by comparing inner and outer pie chart frequencies (Fisher's exact test). (E) ALS-increased DEGs (cortex and whole blood). (F) ALS-decreased DEGs (cortex and whole blood). In (E) and (F), bars show SMD estimates in cortex and circles denote fold-change estimates in spinal cord. The size of circles is proportional to the p-value associated with the fold-change estimate from whole blood (see scale, right margin). (G, H) Top-ranked GO BP terms. GO BP terms most strongly enriched with respect to (G) ALS-increased DEGs or (H) ALS-decreased DEGs from both cortex and whole blood. Enrichment is shown on the horizontal axis (conditional hypergeometric test). The number of DEGs associated with each term is given in parentheses (left margin). Example DEGs are listed within each figure.

Figure S51. Overlap between DEGs and ALS-associated genes (NHGRI-EBI GWAS catalog). (A) Manhattan plot. ALS-associated genes are shown with associated -log10-transformed p-value (vertical axis). Genes linked to the most significant loci are listed (right). An asterisk is used to denote genes having detectable expression in cortex (included in the differential expression meta-analysis). (B) SMD estimates of ALS-associated genes (blue: SMD < 0; red: SMD > 0). The percentage of genes with SMD > 0 (red) or SMD < 0 is shown (upper left). This percentage is also represented by the inner pie chart (lower right). The outer pie chart (lower right) shows the percentage of genes with SMD > 0 (red) and SMD < 0 (blue) among all meta-analysis genes. The p-value was obtained by comparing frequencies between the inner and outer pie charts (Fisher's exact test). (C - E) Venn diagrams. Venn diagrams compare overlap between DEGs and ALS-associated genes. Significance of overlap was evaluated using Fisher's exact test (bottom p-values). (F) Overlap between DEGs and ALS-associated genes. Overlap is shown between ALS-increased DEGs (red) or ALS-decreased DEGs (blue) and ALS-associated genes located varying distances from disease-associated genetic variants (horizontal axis). An asterisk (top) denotes instances where overlap is larger than expected (P < 0.05, Fisher's exact test). (G -I) Average distance between ALS DEGs and ALS-associated genetic variants. The distance between each ALS DEG and the nearest GWAS locus was calculated and the average of these distances among DEGs is shown (arrows). Background null distributions were obtained by randomly selecting gene sets of the same size and calculating the average distance to GWAS loci in the same fashion. P-values (upper right) were generated by comparing observed values (arrows) to the null distribution shown. (J - M) Gene annotations enriched among ALS-associated genes. Annotations enriched among ALS-associated genes were identified (P < 0.05, conditional hypergeometric test). Enrichment p-values were then calculated with respect to the set of ALS-increased DEGs (horizontal axis) and ALS-decreased genes (vertical axis). The number of annotations enriched with respect to ALS-increased (red) or ALS-decreased (blue) DEGs is shown (upper right) (P < 0.05, Fisher's exact test). P-values were obtained by comparing the number of enriched annotations to that observed to that observed in randomly selected gene sets of the same size.

Figure S52. Transcription factor (TF) binding sites located near DEGs. (A) Motifs with increased proximity to ALS-increased DEGs. (B) Motifs with increased proximity to ALS-decreased DEGs. In (A) and (B), fractions (within bars) show the average distance (kb) between

the listed motif and the transcription start site (TSS) associated with DEGs (numerator) and non-DEGs (denominator). The value of this ratio is plotted (horizontal axis) and listed (right margin). P-values were generated by comparing DEG motif-TSS distances to non-DEG motif-TSS distances (Wilcoxon rank sum test). Primary and reverse complement sequence logos for each motif are shown (far right).

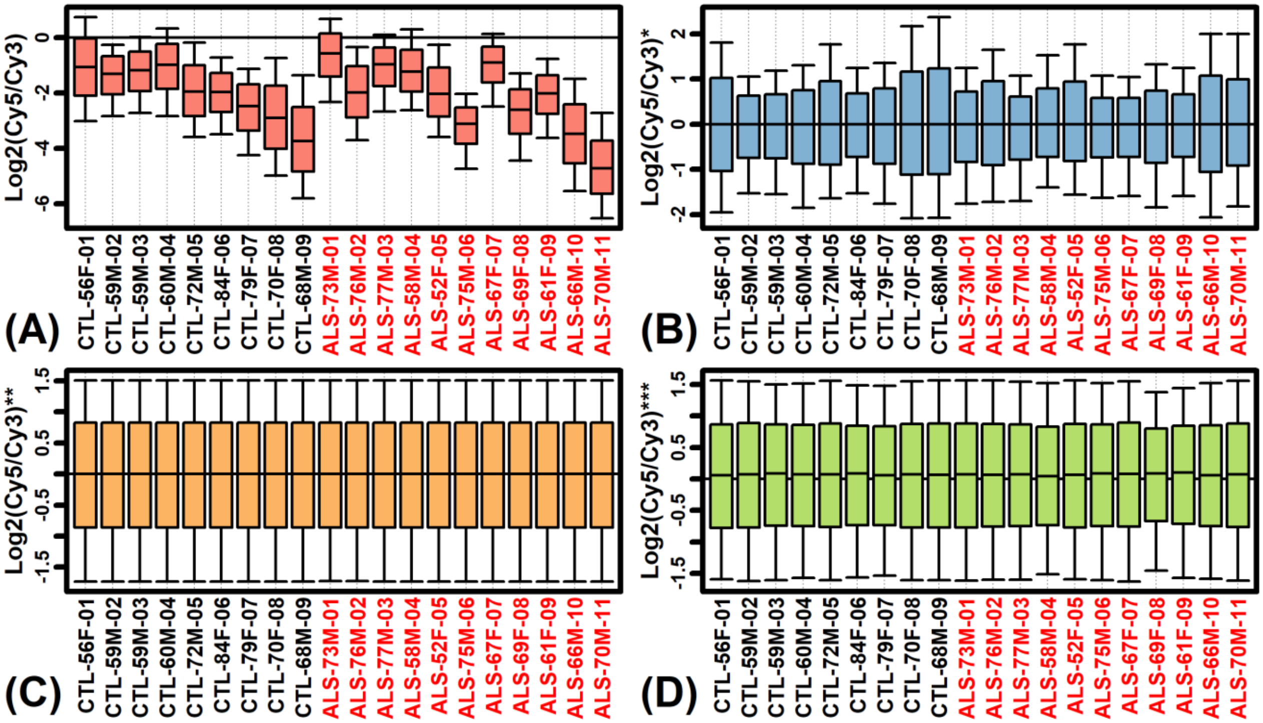

Figure S1. Expression ratio distributions (GSE4595).

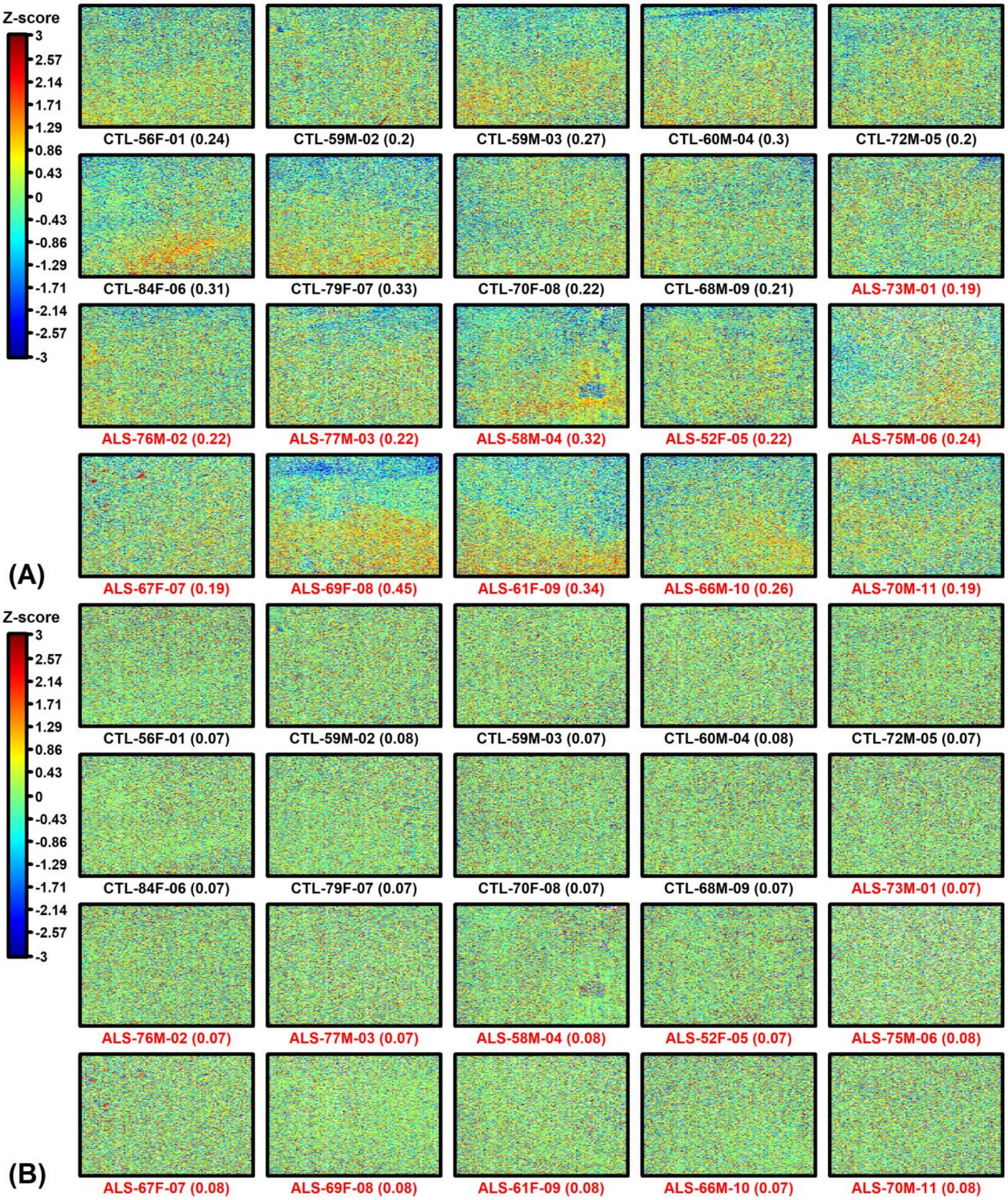

Figure S2. Microarray pseudoimages (GSE4595).

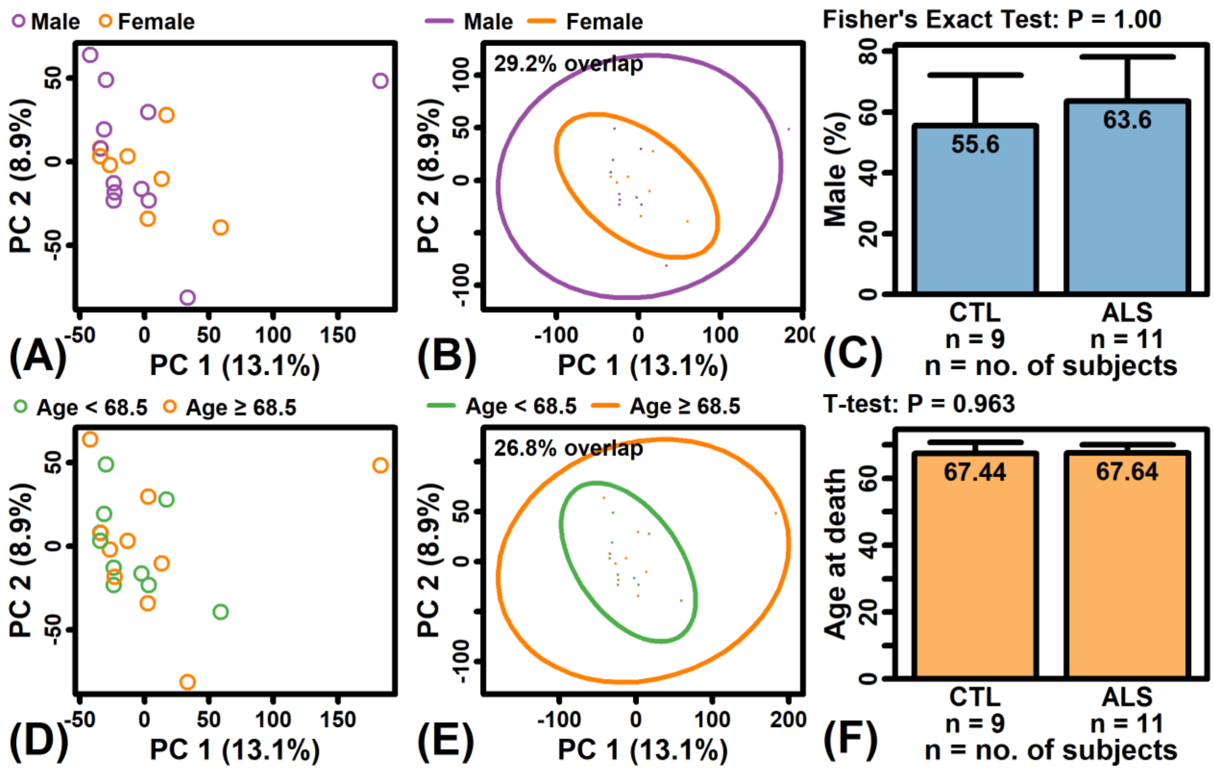

Figure S3. Covariate variables: sex and age at death (GSE4595).

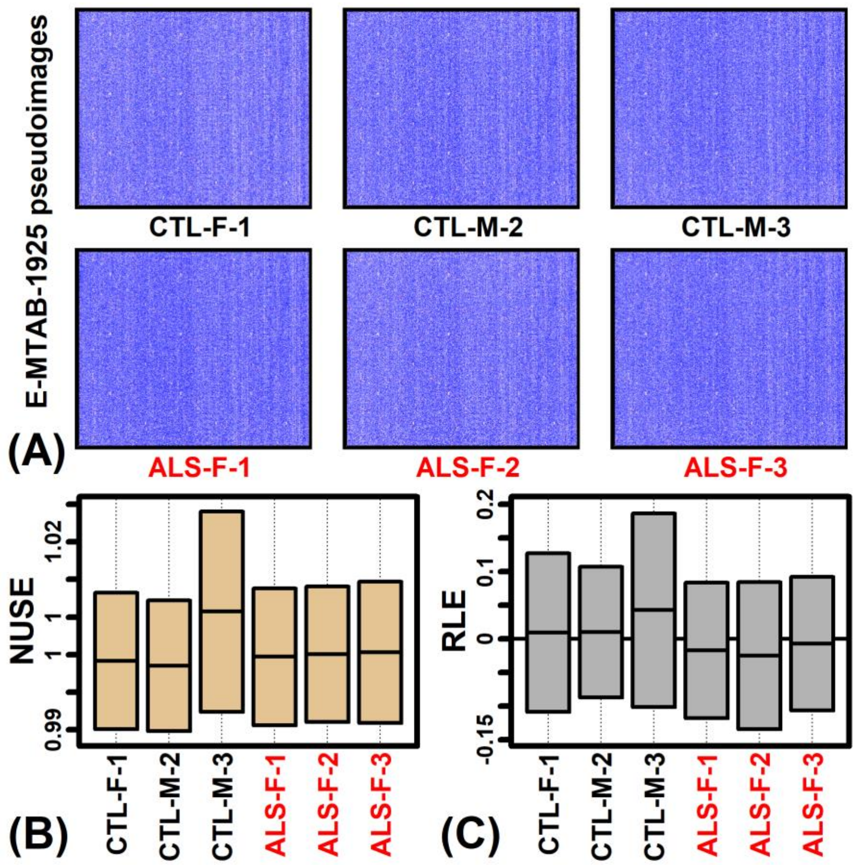

Figure S4. Microarray pseudoimages and probe-level metrics (E-MTAB-1925).

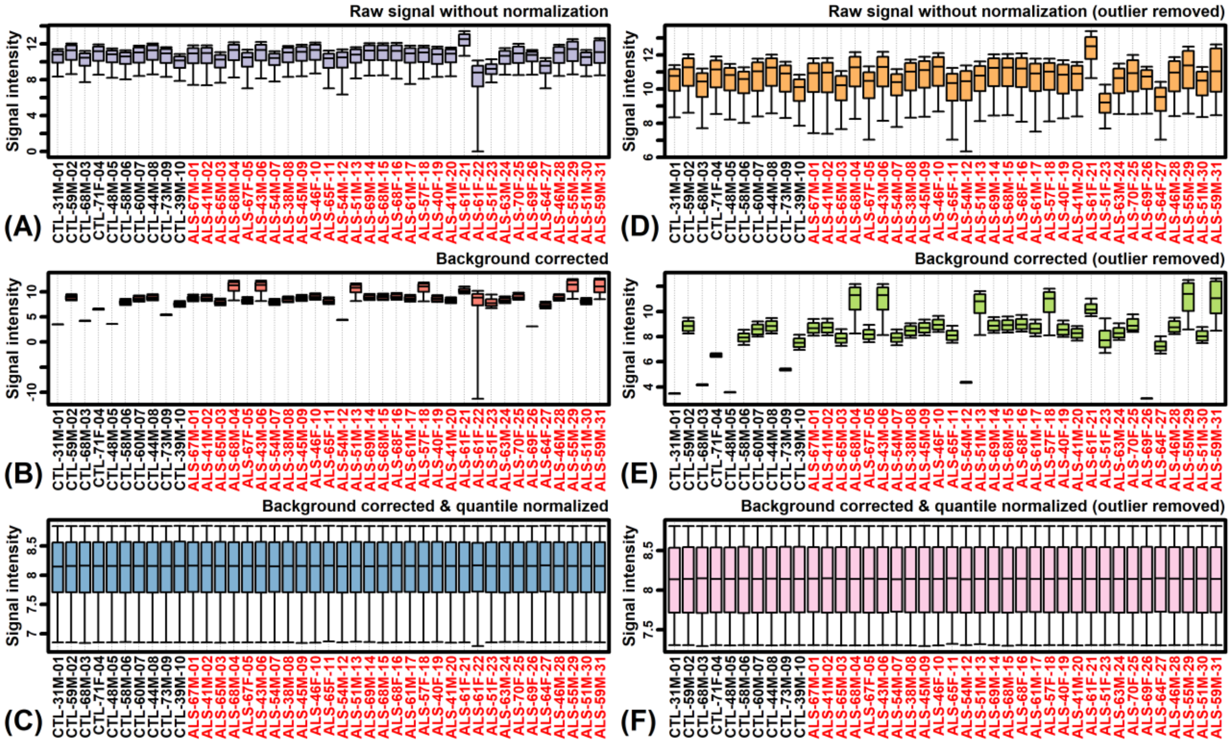

Figure S5. Expression signal distributions (E-MTAB-2325).

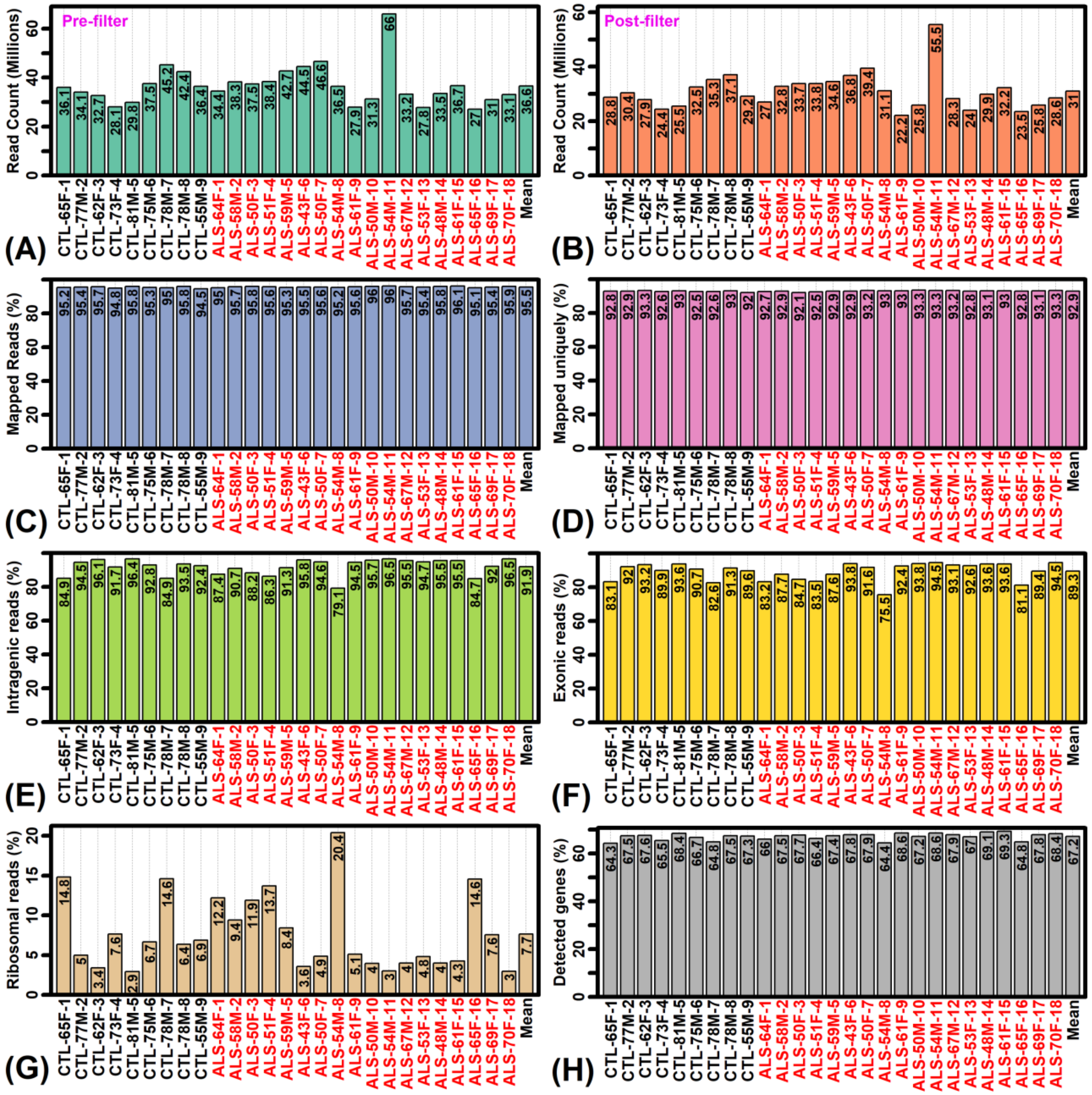

Figure S6. Read mapping quality control metrics (GSE67196).

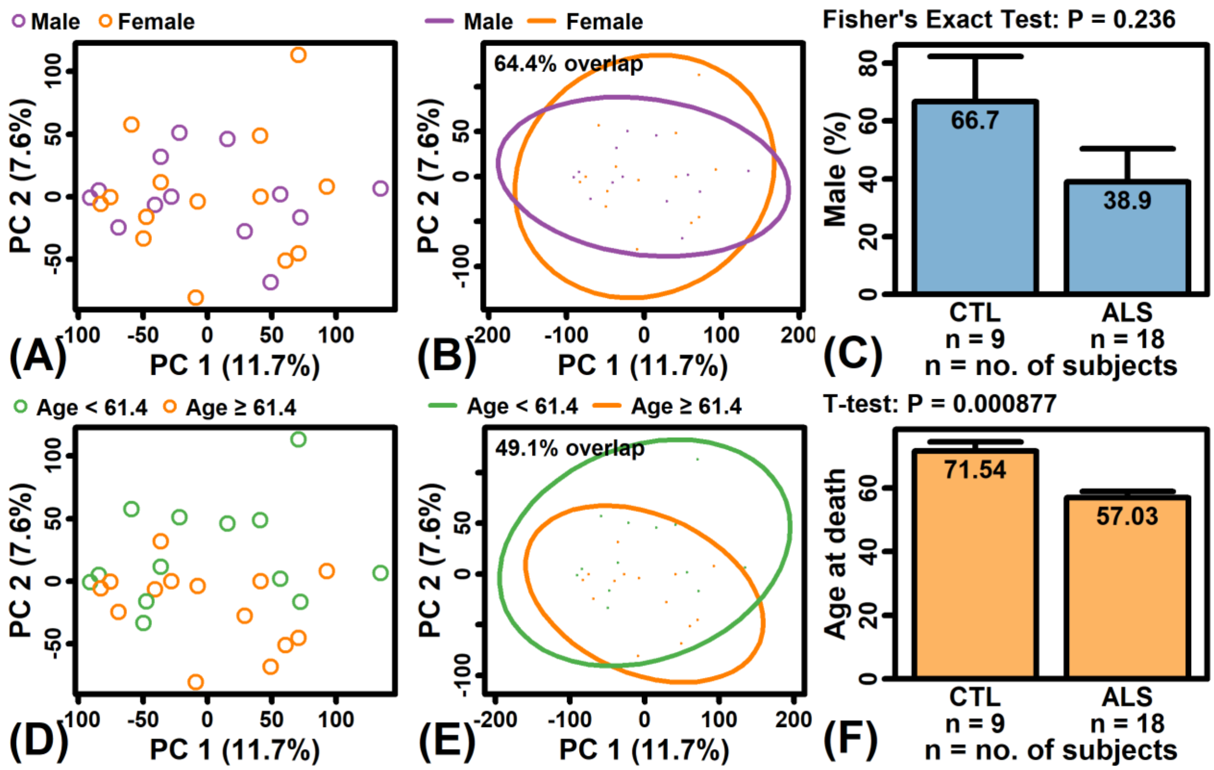

Figure S7. Covariate variables: sex and age at death (GSE67196).

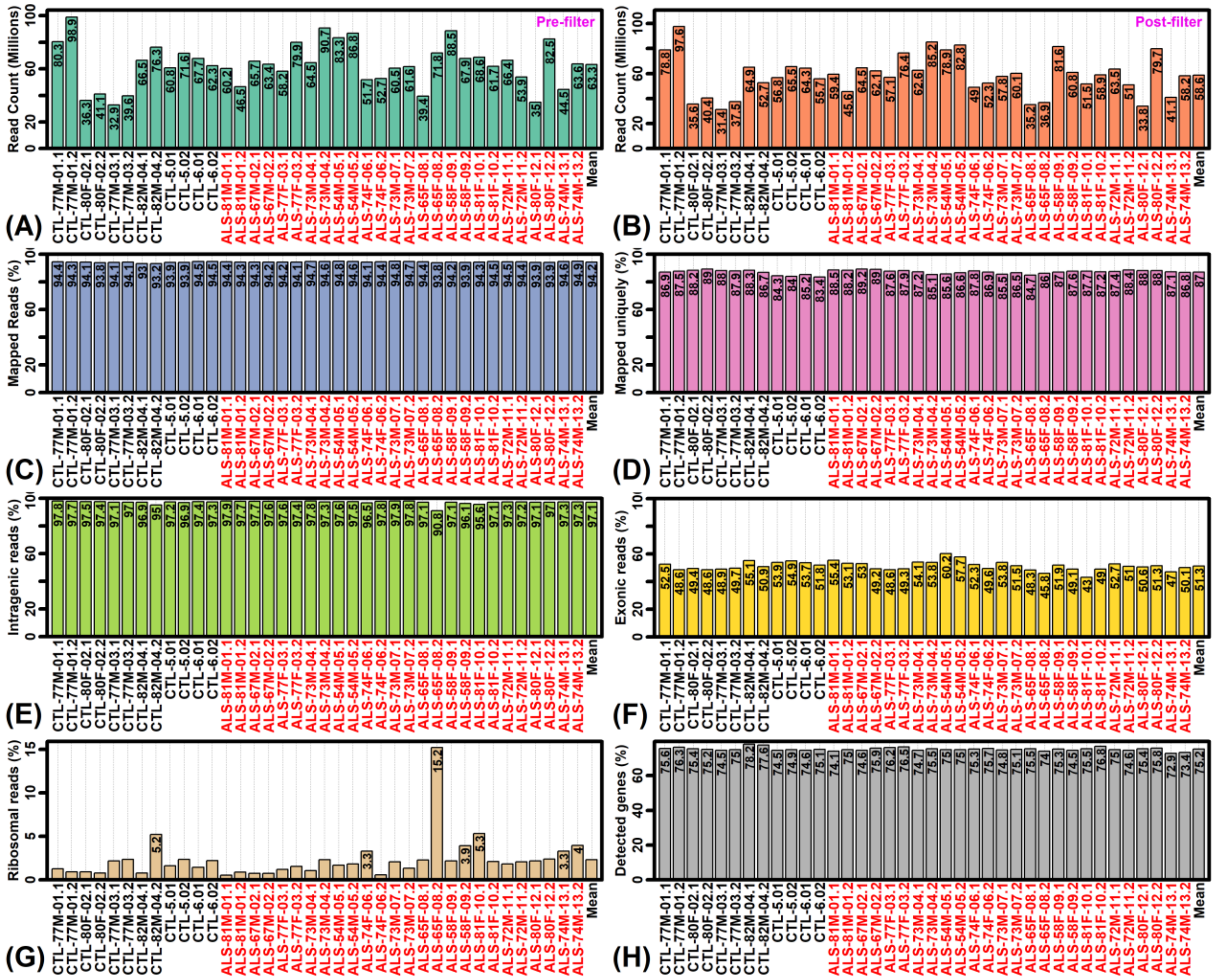

Figure S8. Read mapping quality control metrics (GSE122649).

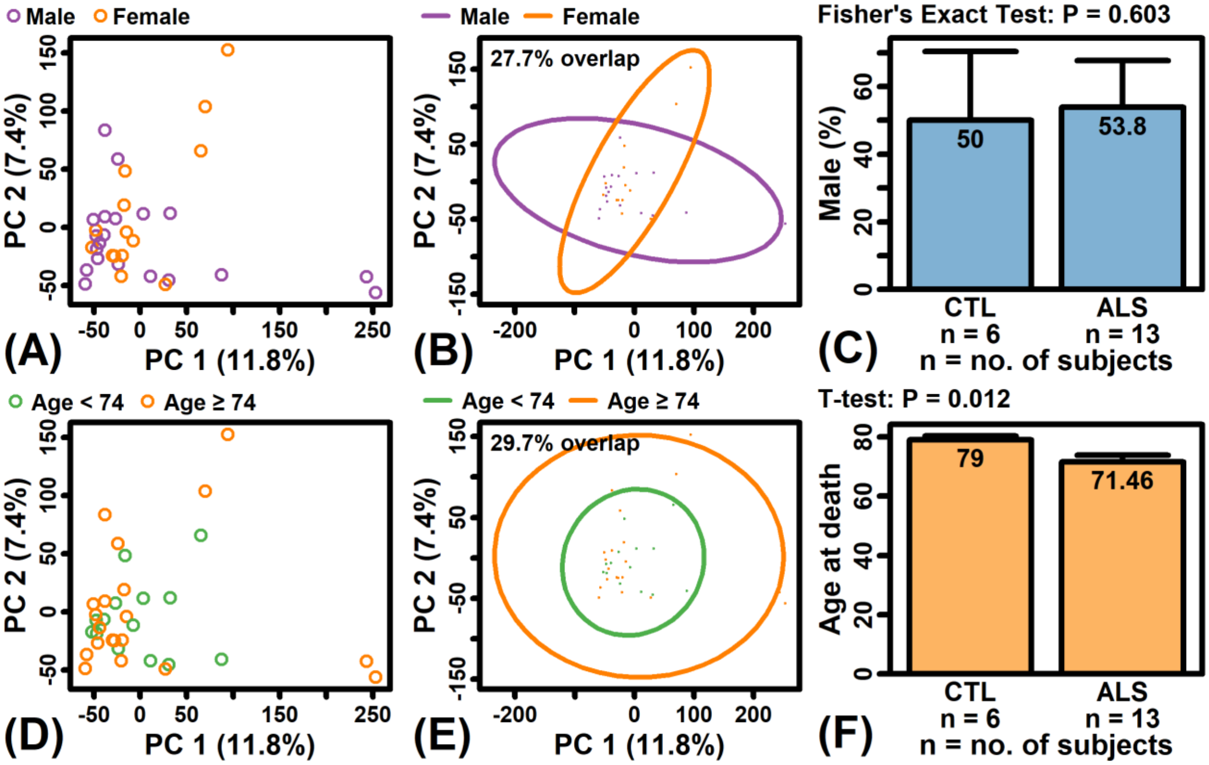

Figure S9. Covariate variables: sex and age at death (GSE122649).

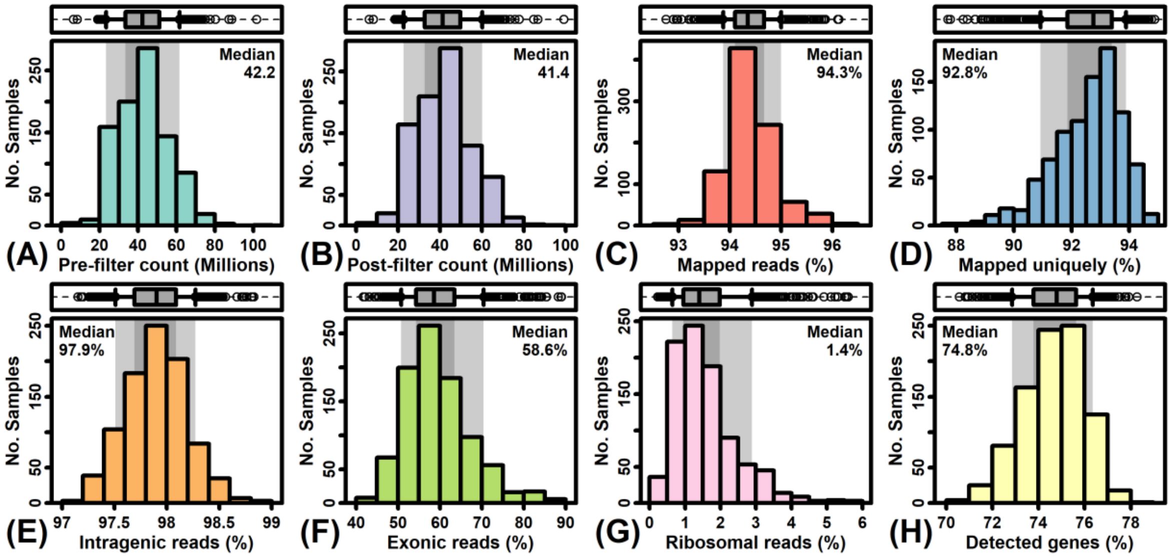

Figure S10. Read mapping quality control metrics (GSE137810).

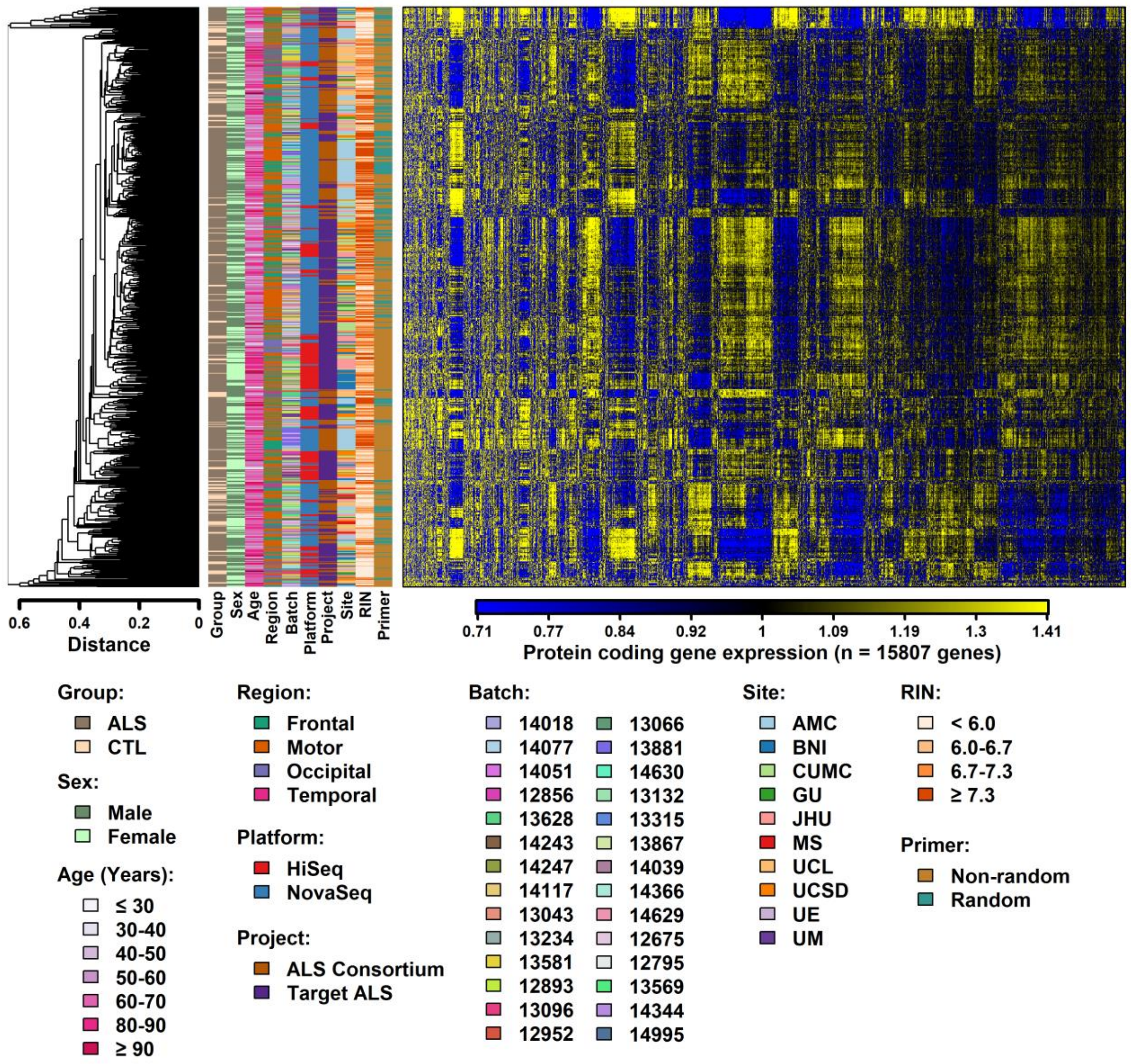

Figure S11. Gene expression heatmap (GSE137810).

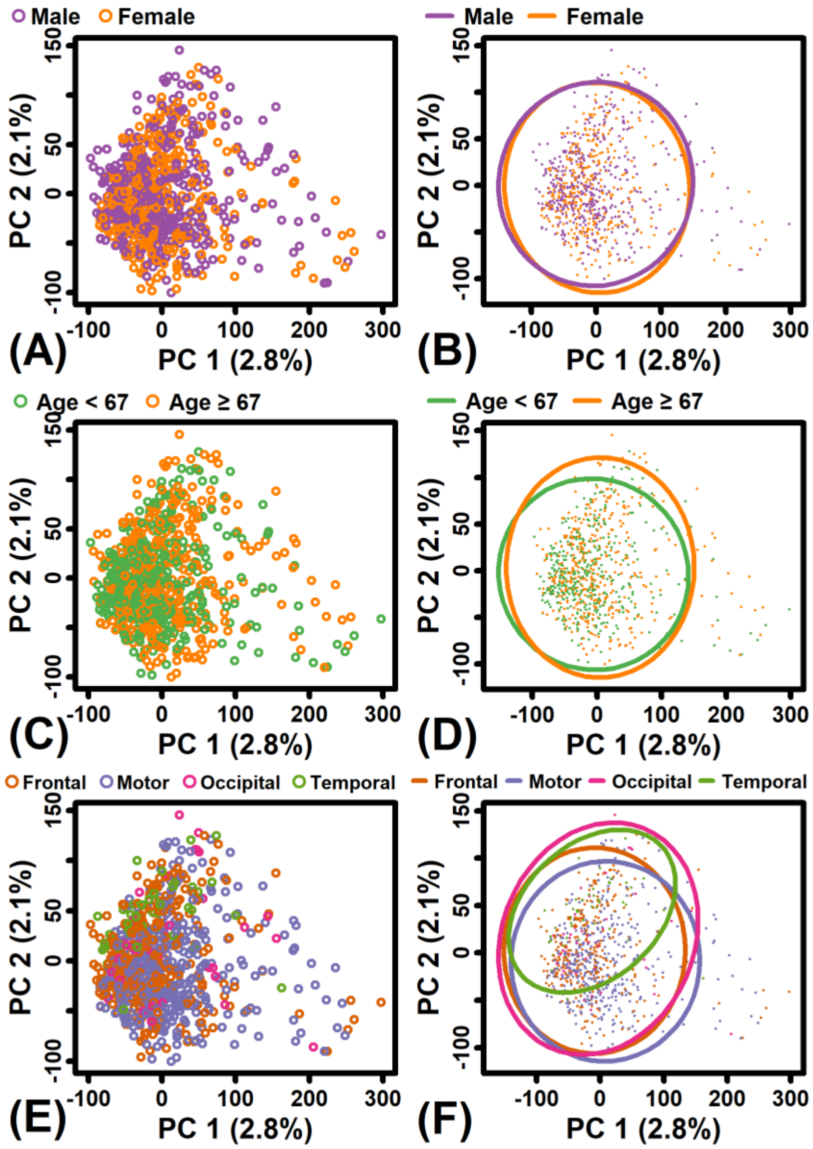

Figure S12. Principal component (PC) plots (biologic factors; GSE137810).

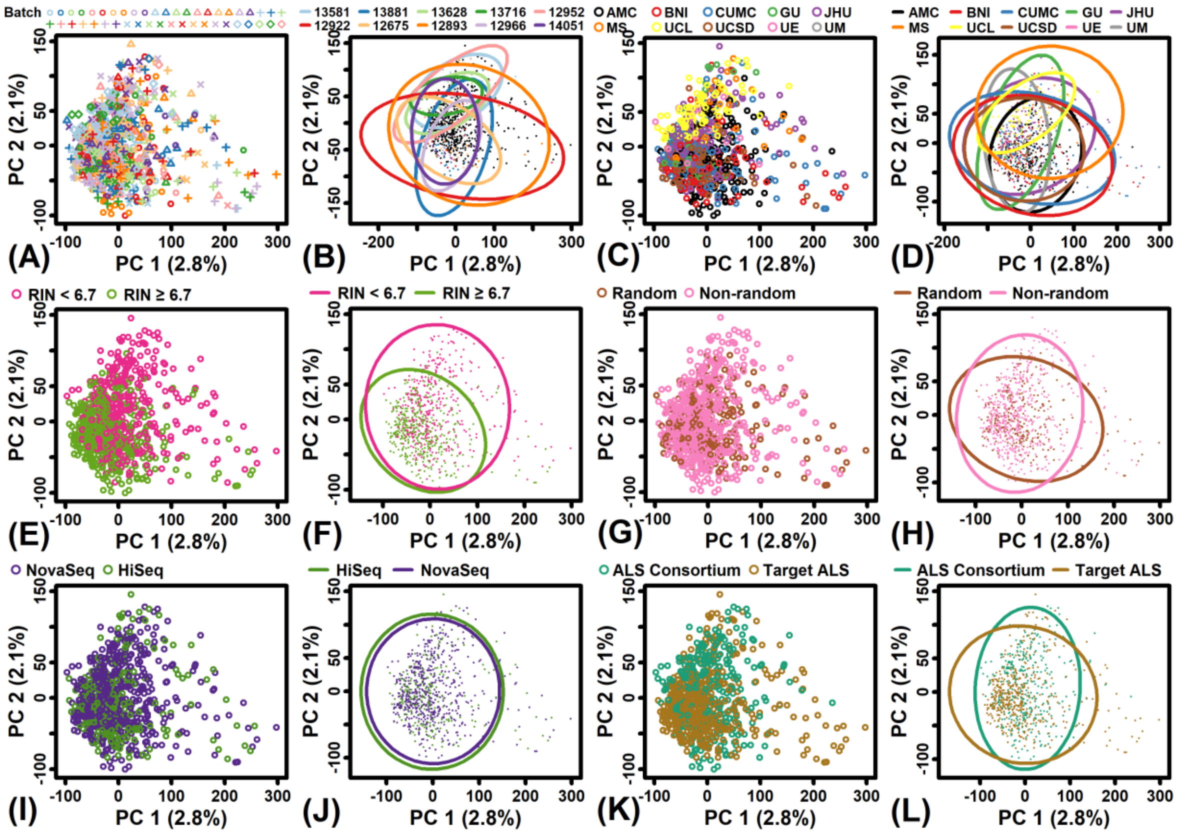

Figure S13. Principal component (PC) plots (technical factors; GSE137810).

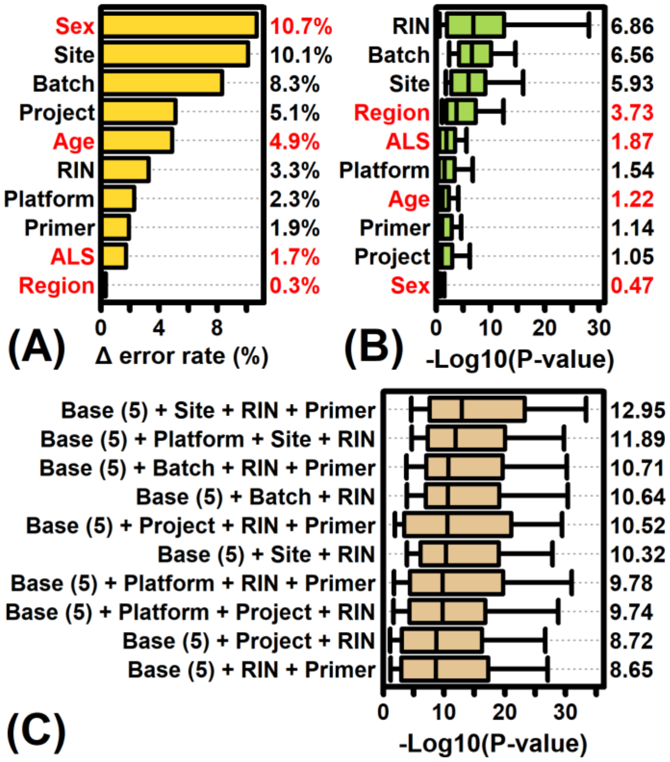

Figure S14. Model selection (GSE137810).

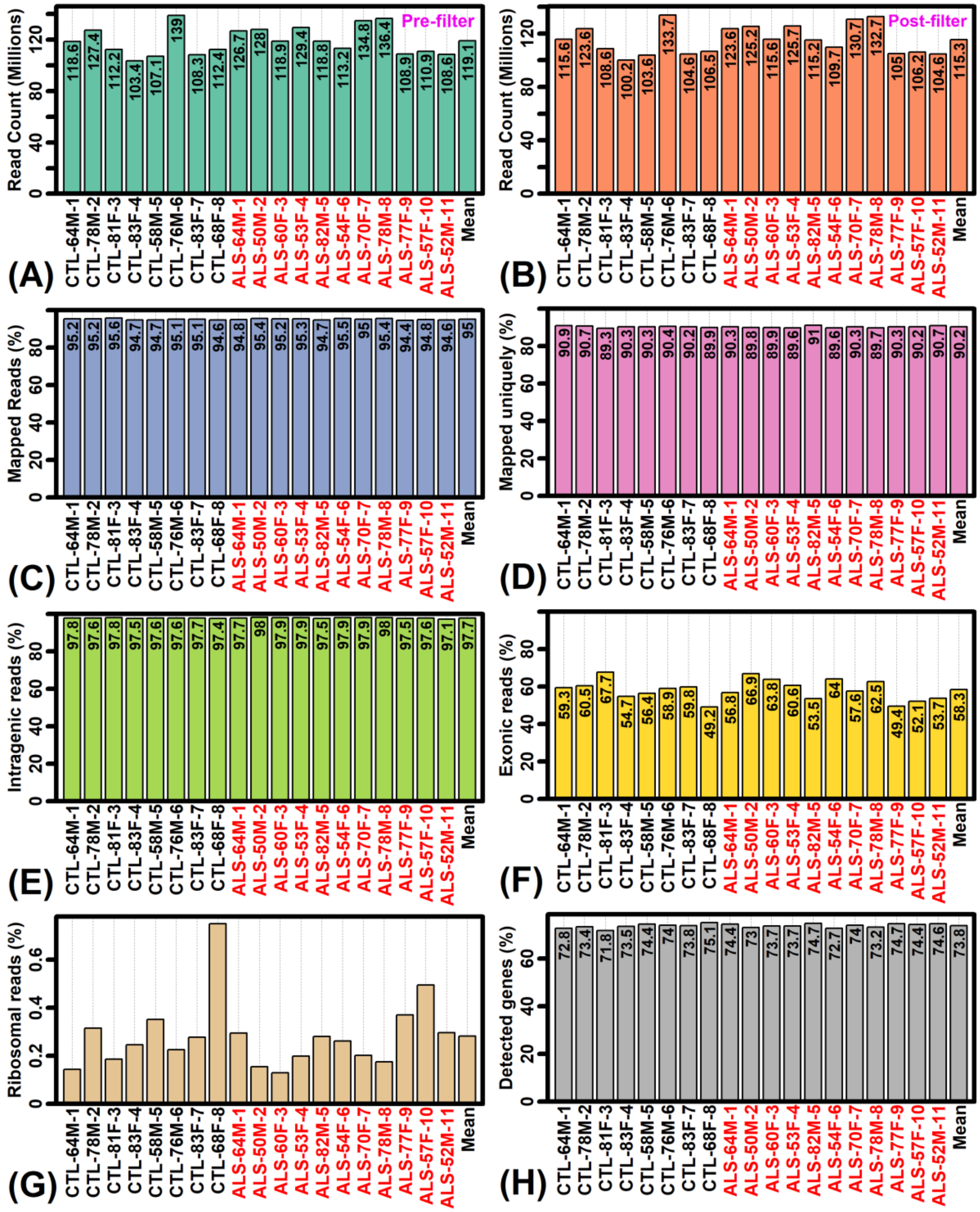

Figure S15. Read mapping quality control metrics (EGAS00001004286).

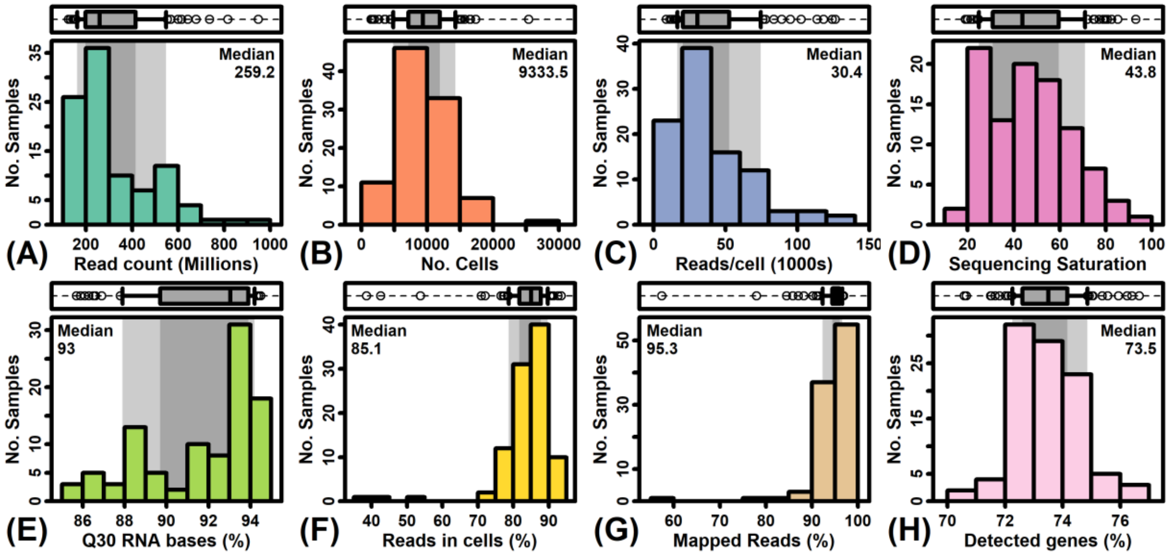

Figure S16. Read mapping quality control metrics (GSE174332).

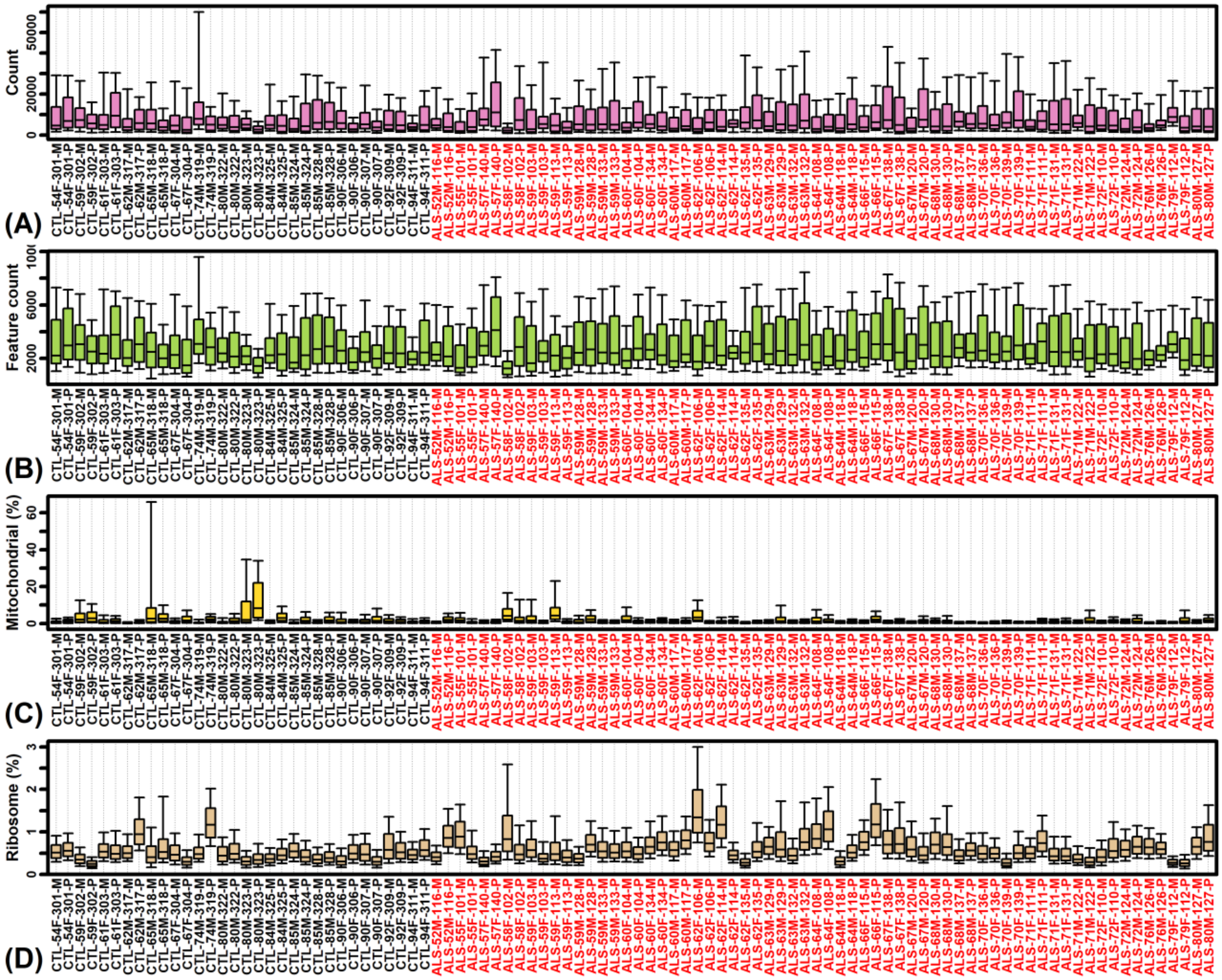

Figure S17. Distribution of counts, features, and mitochondrial/ribosomal reads among cells (GSE174332).

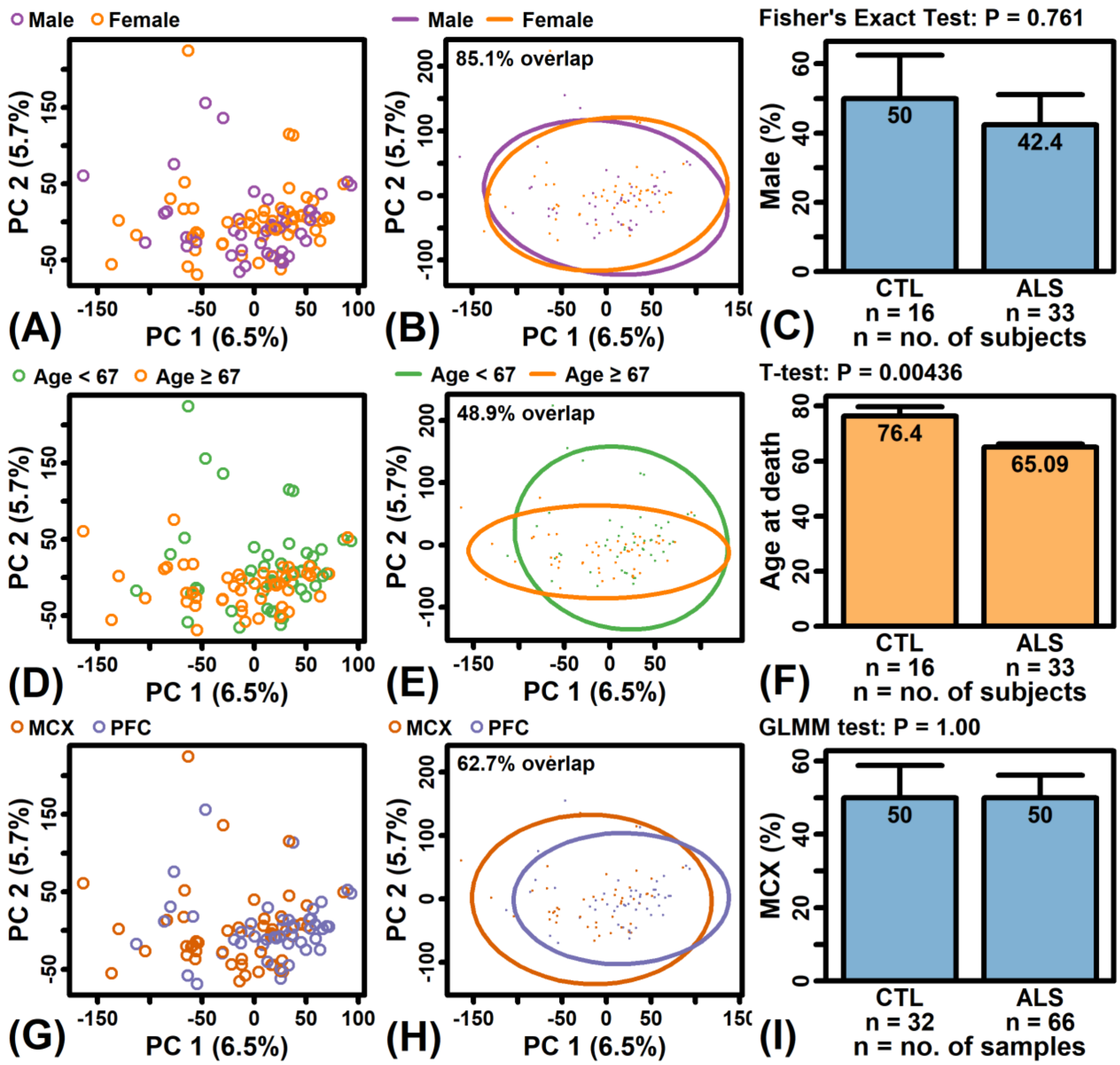

Figure S18. Covariate variables: sex, age at death and cortical region (GSE174332).

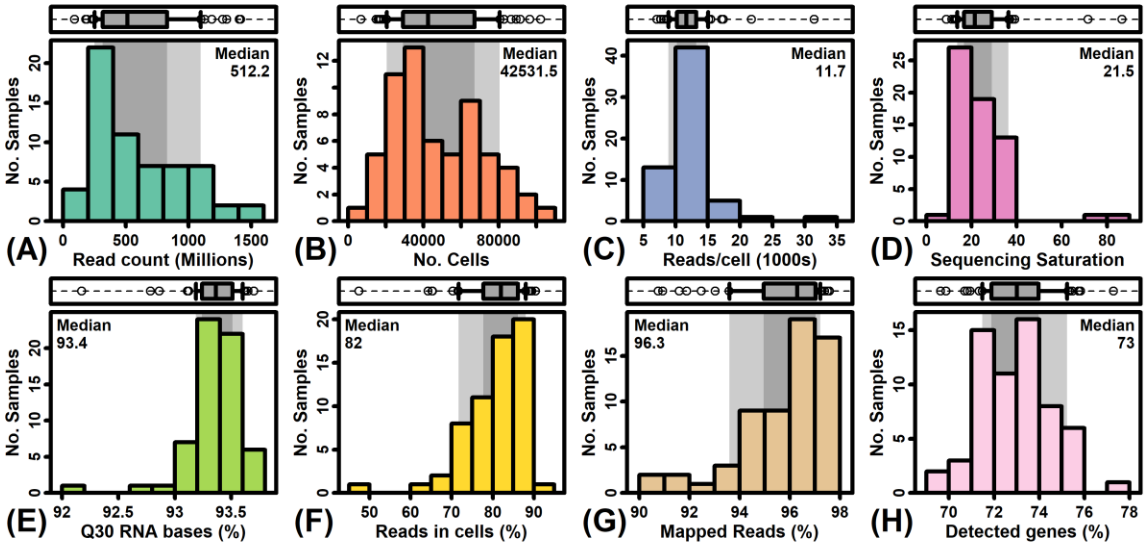

Figure S19. Read mapping quality control metrics (syn45351388).

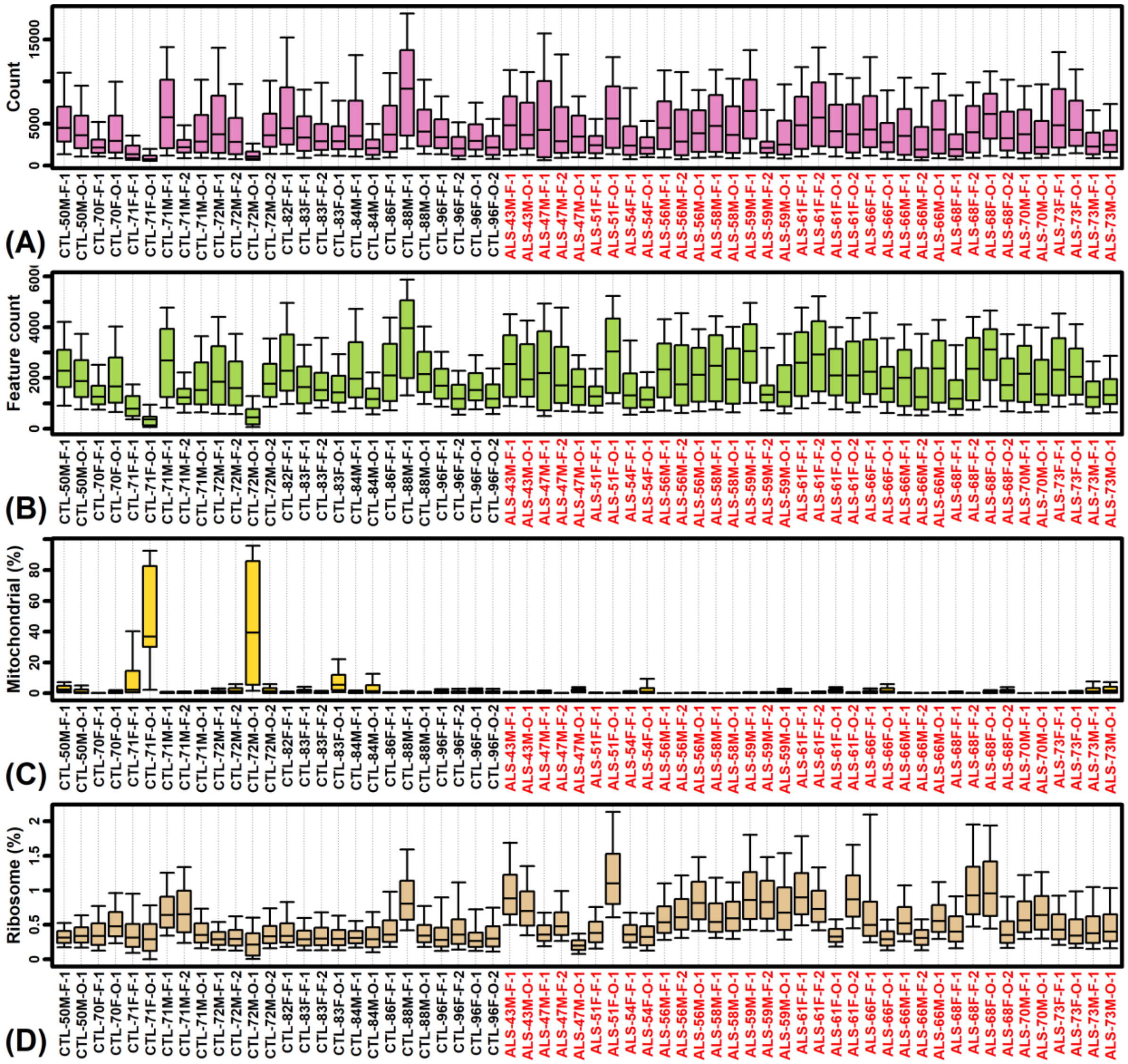

Figure S20. Distribution of counts, features, and mitochondrial/ribosomal reads among cells (syn45351388).

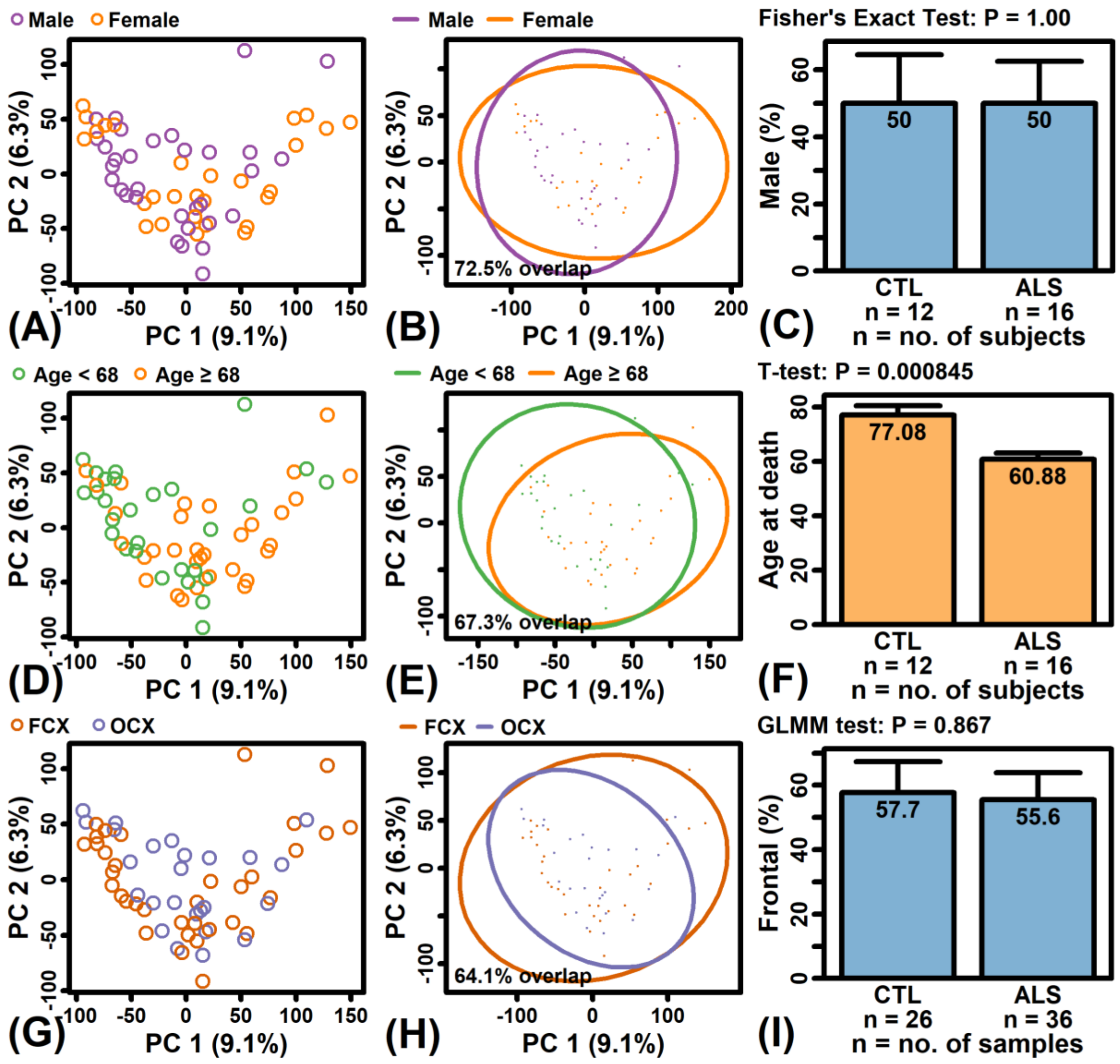

Figure S21. Covariate variables: sex, age at death and cortical region (syn45351388).

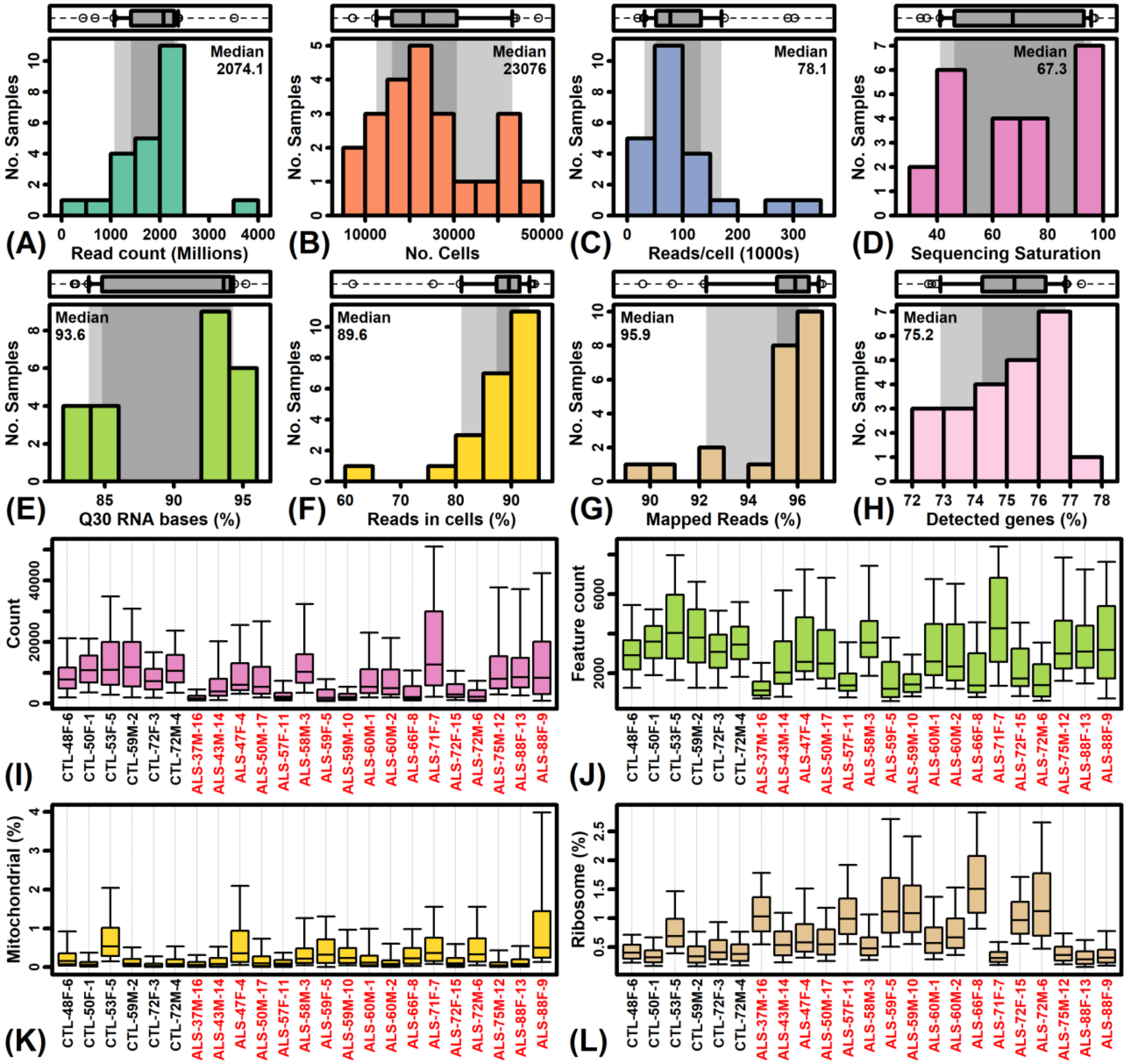

Figure S22. Read mapping quality control metrics and distribution of counts, features, and mitochondrial/ribosomal reads among cells (PRJNA918304).

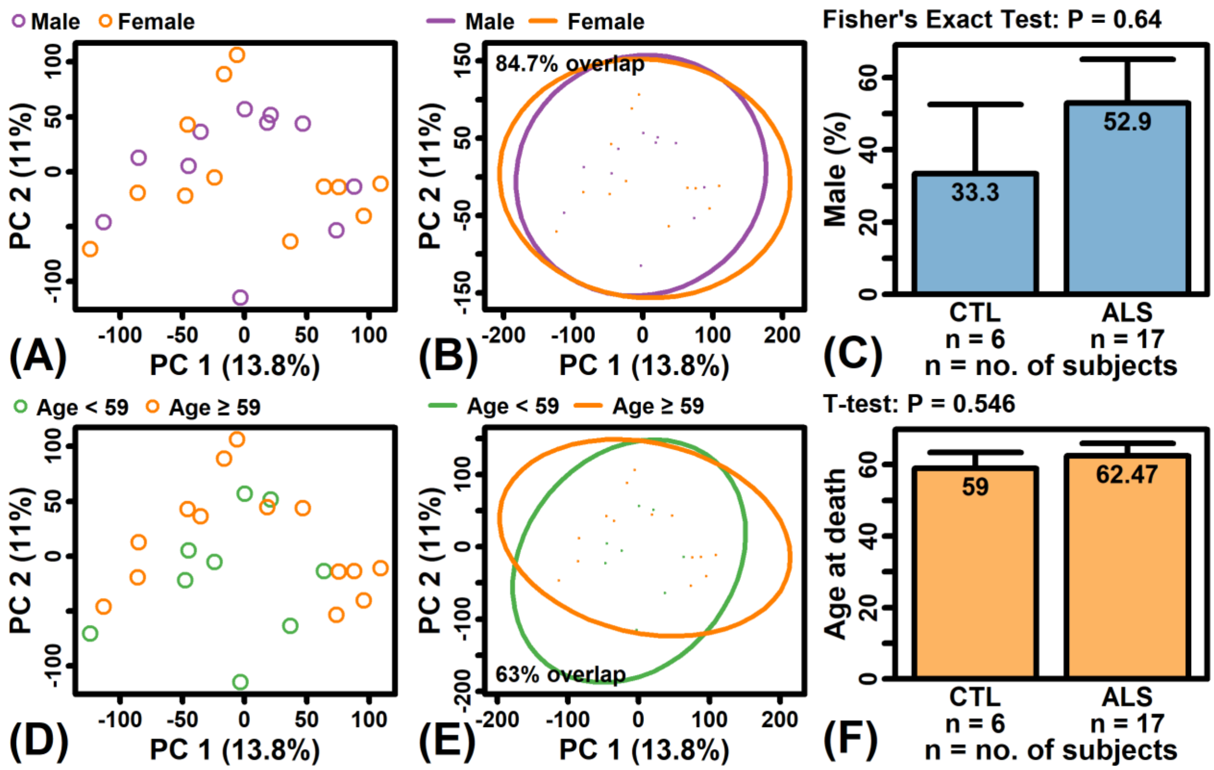

Figure S23. Covariate variables: sex and age at death (PRJNA918304).

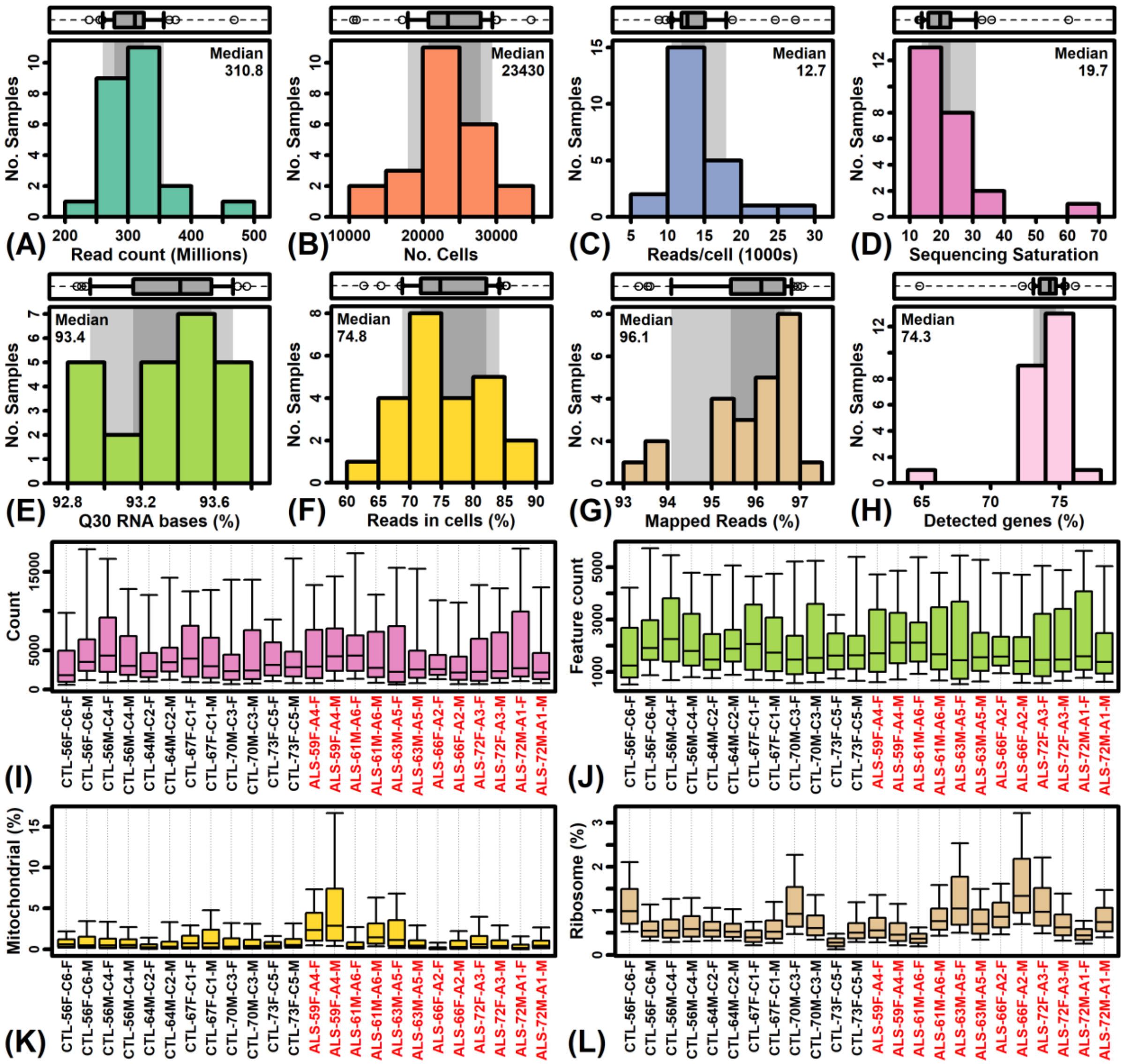

Figure S24. Read mapping quality control metrics and distribution of counts, features, and mitochondrial/ribosomal reads among cells (GSE219280).

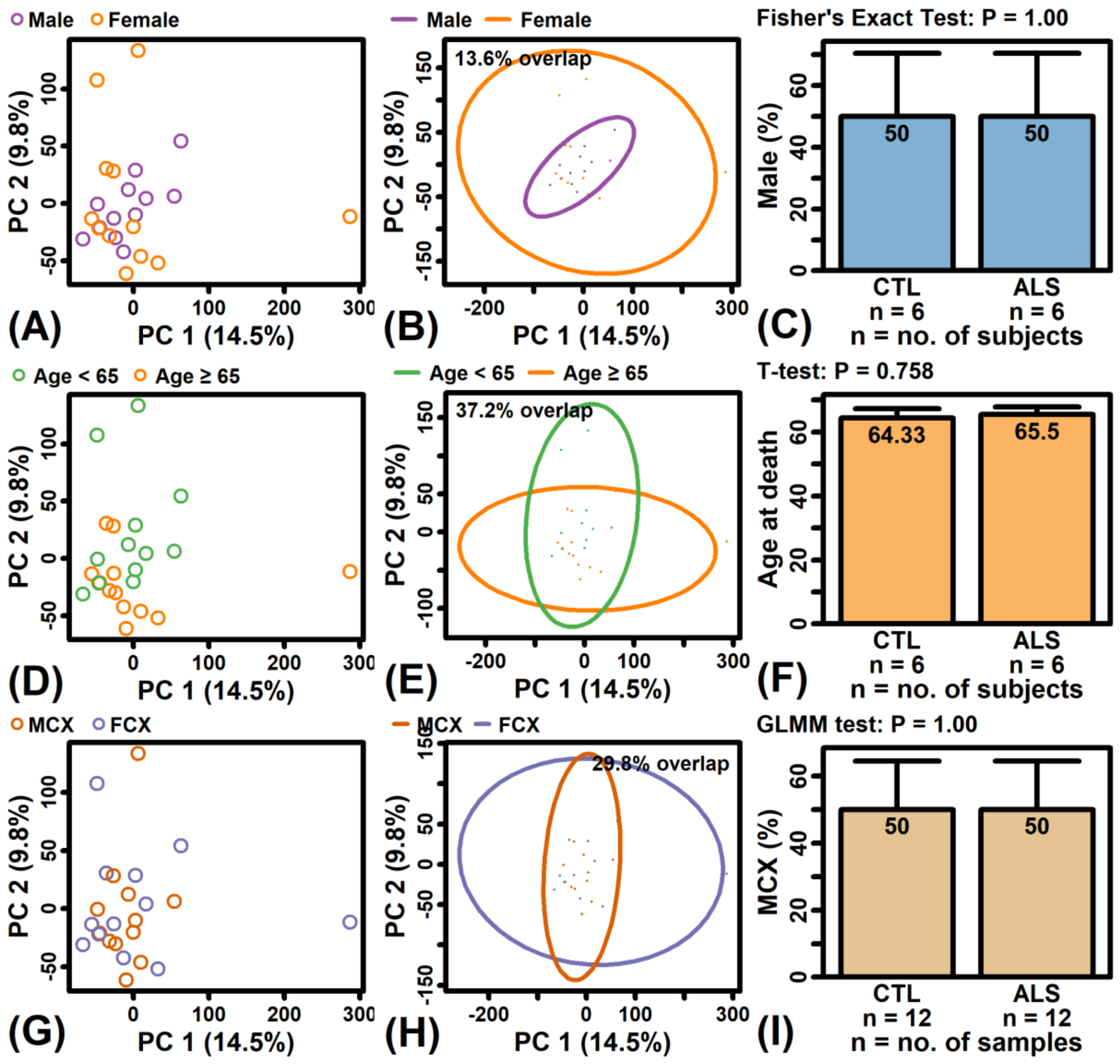

Figure S25. Covariate variables: sex, age at death and cortical region (GSE219280).

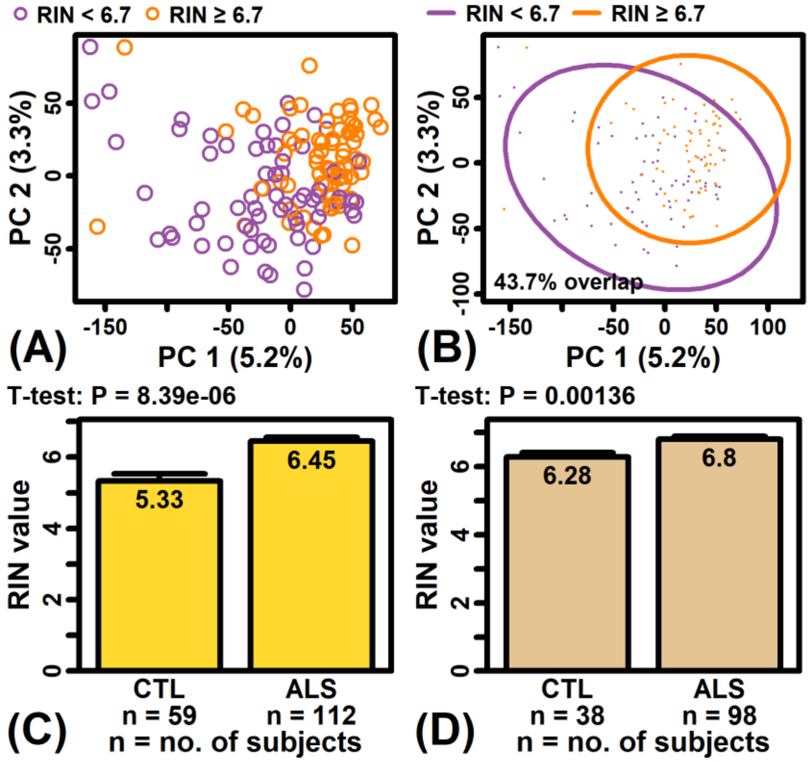

Figure S26. Covariate variable: RIN (KCL BrainBank).

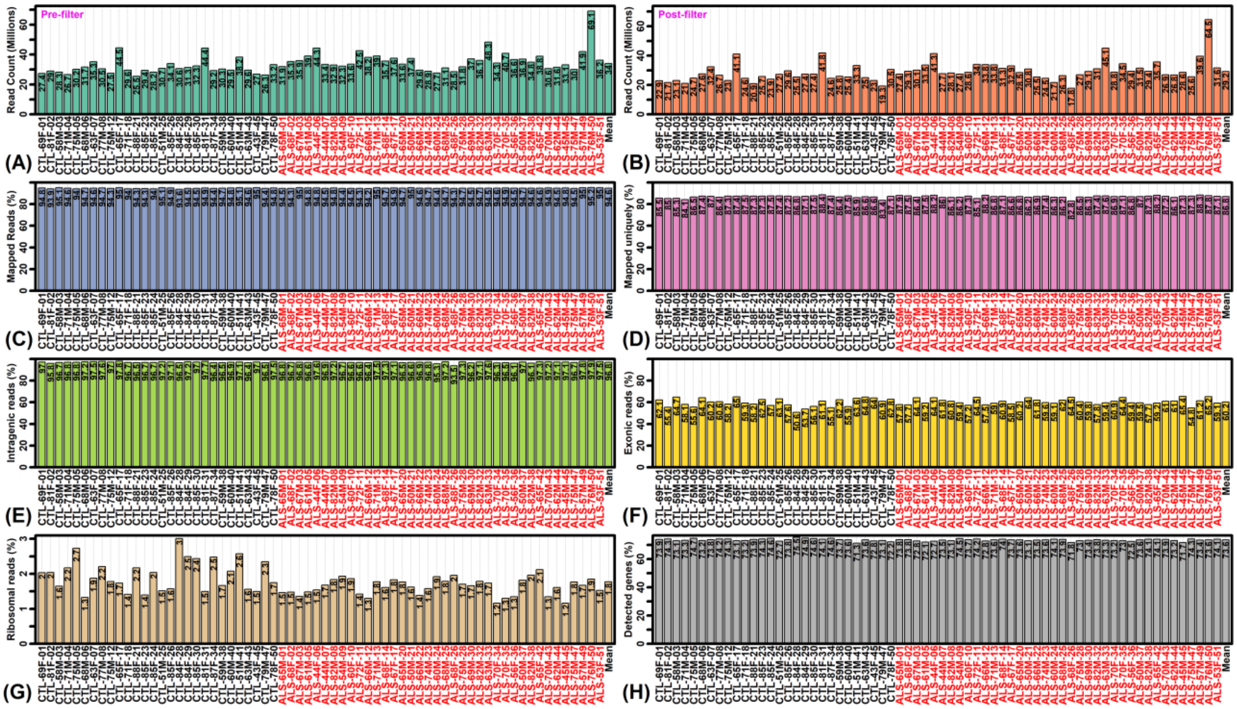

Figure S27. Read mapping quality control metrics (EGAS00001007318).

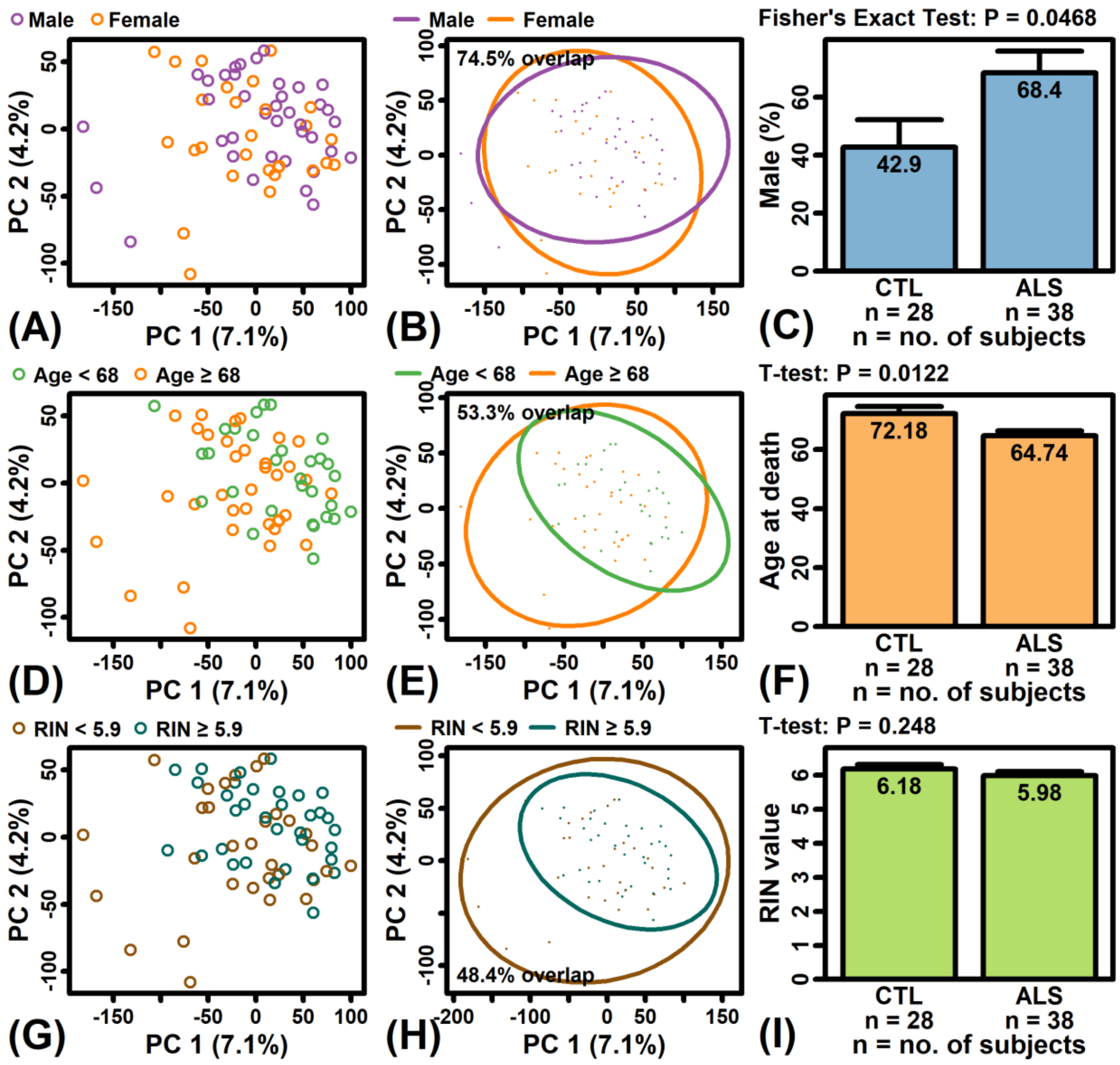

Figure S28. Covariate variables: sex, age at death and RIN (EGAS00001007318).

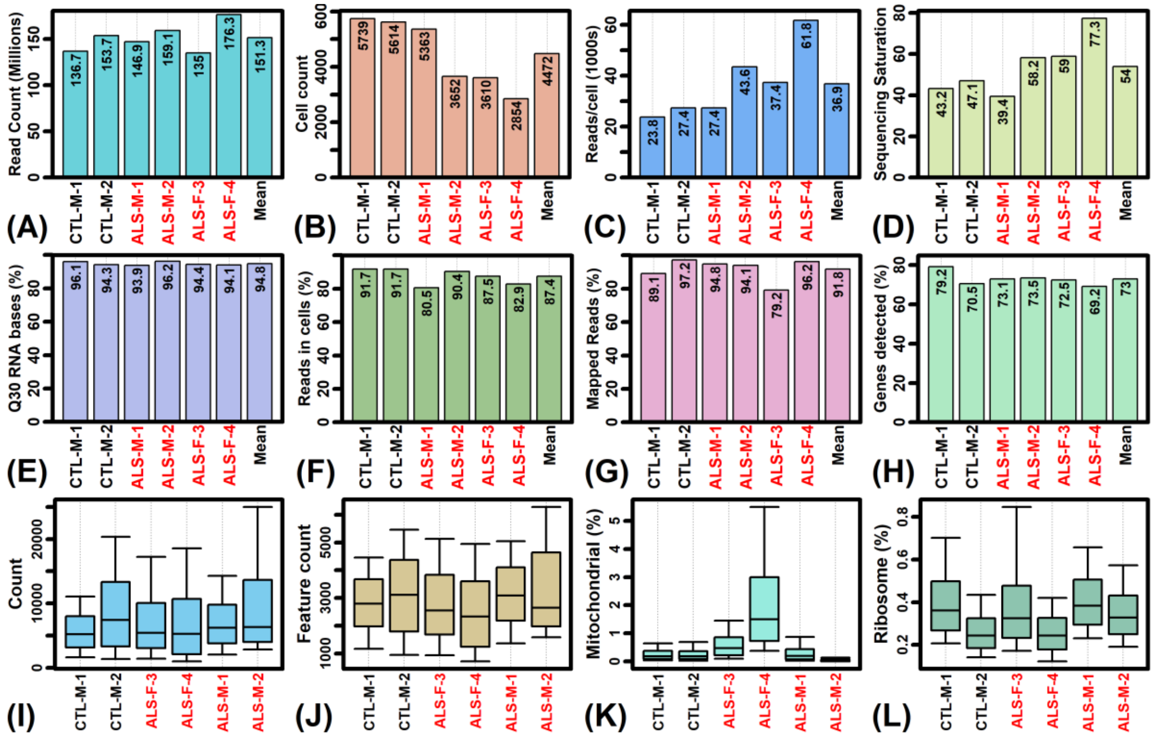

Figure S29. Read mapping quality control metrics and distribution of counts, features, and mitochondrial/ribosomal reads among cells (GSE271156).

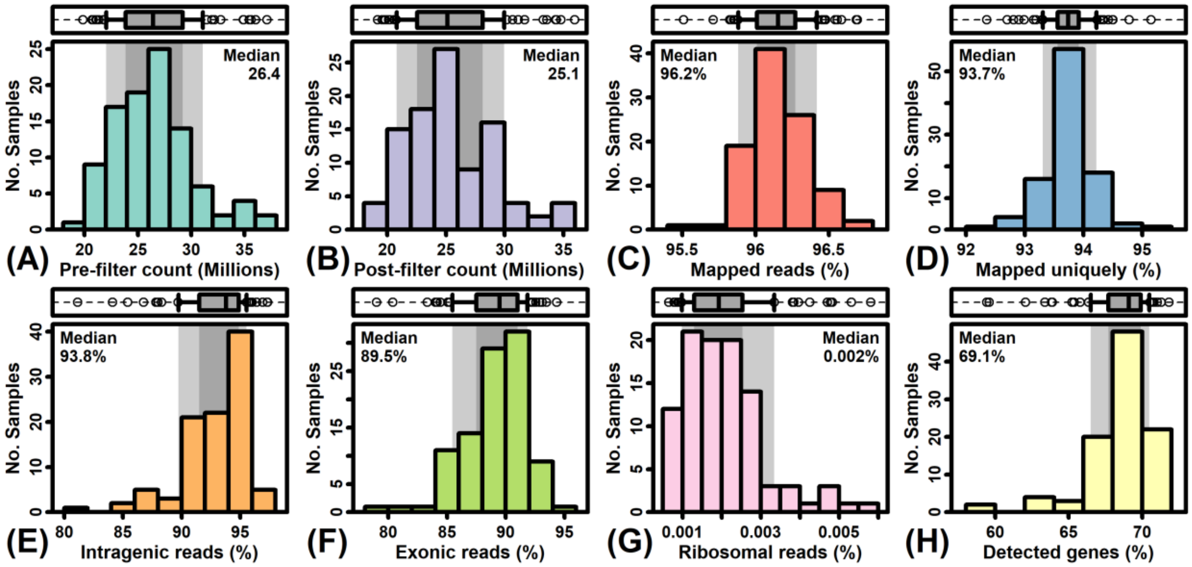

Figure S30. Read mapping quality control metrics (NG00178).

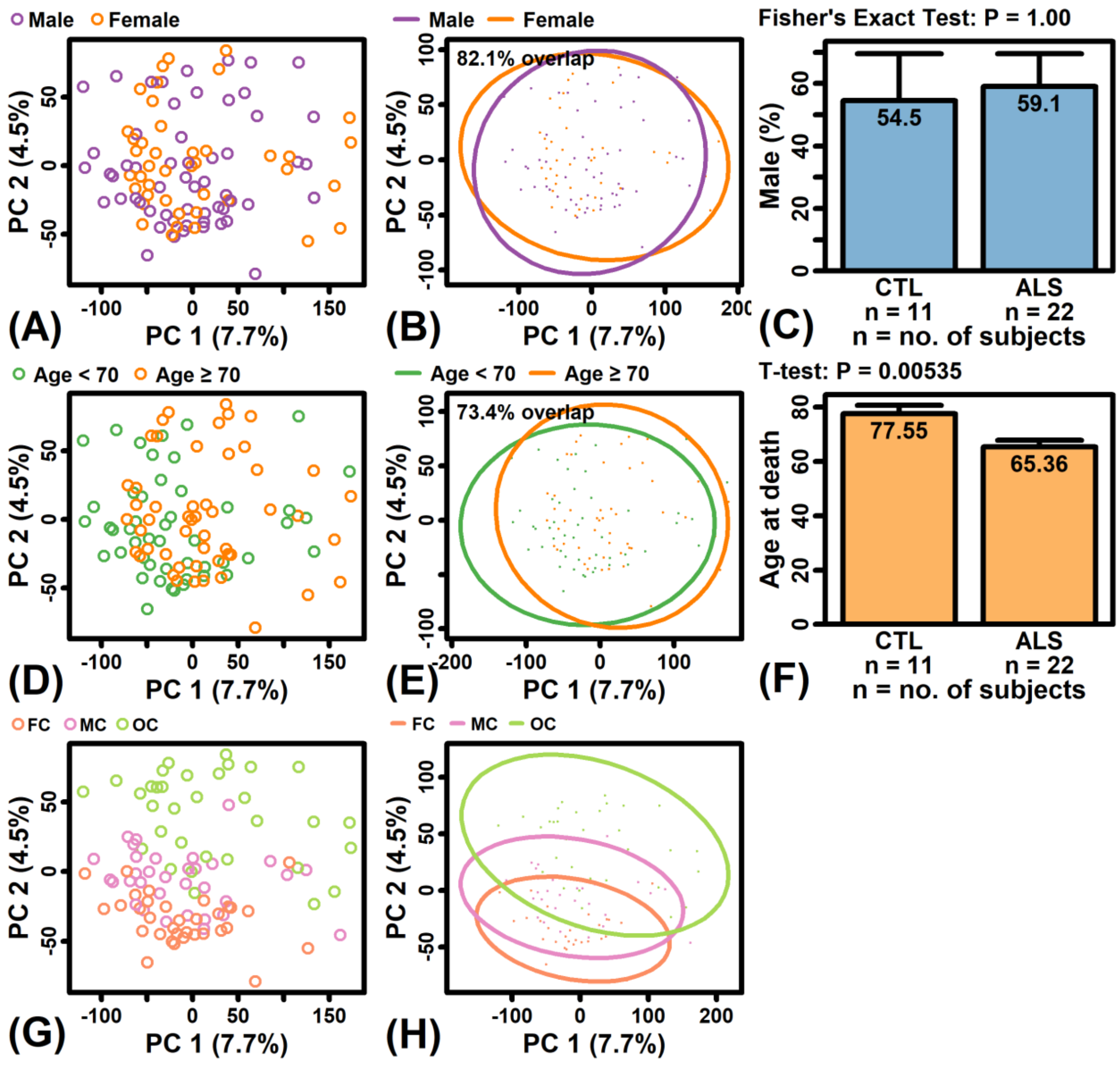

Figure S31. Covariate variables: sex, age at death and cortex region (NG00178).

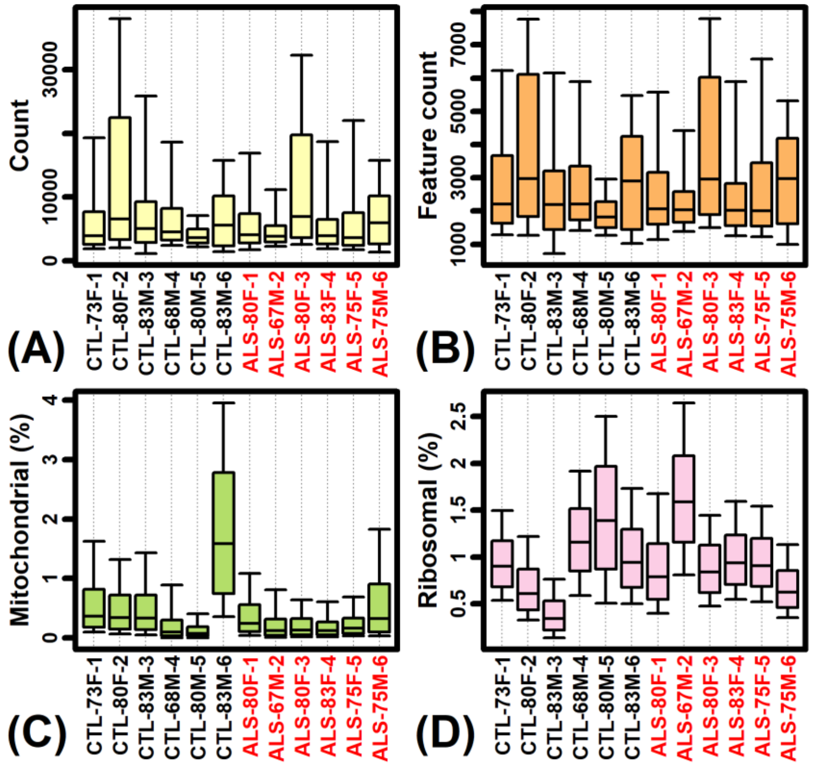

Figure S32. Distribution of counts, features, and mitochondrial/ribosomal reads among cells (JGAS000852).

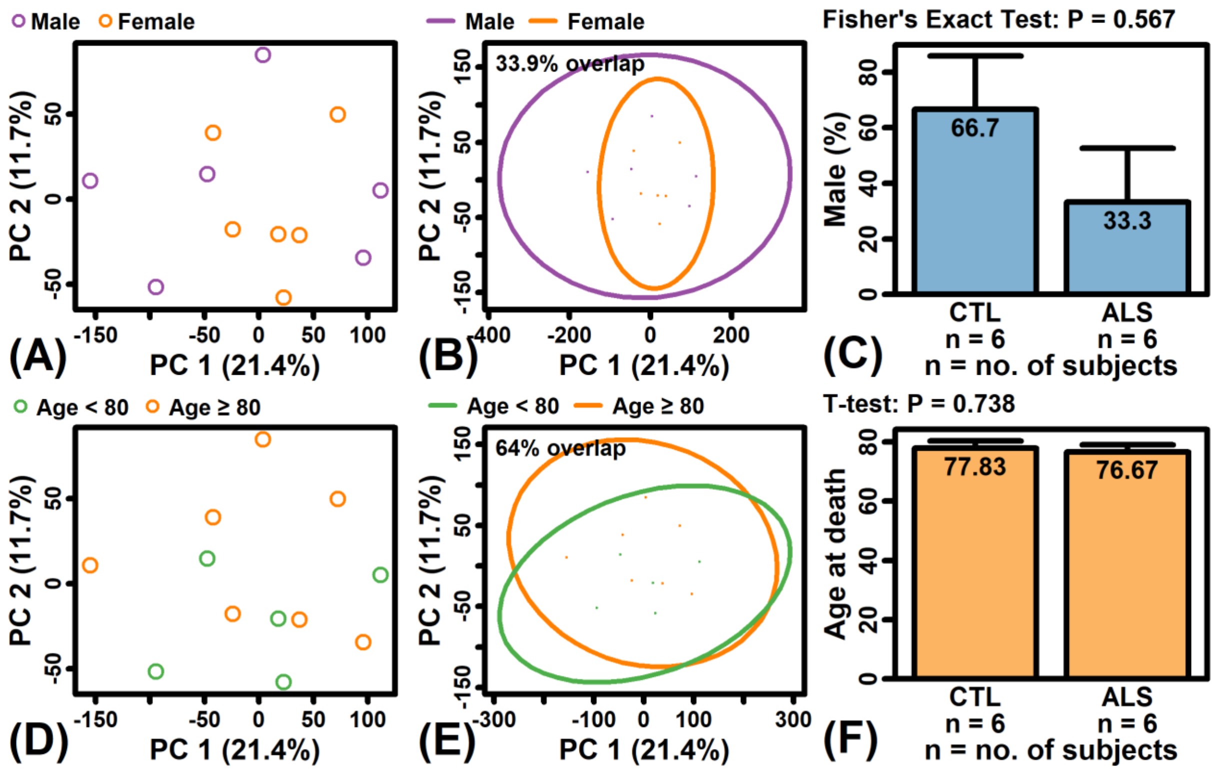

Figure S33. Covariate variables: sex and age at death (JGAS000852).

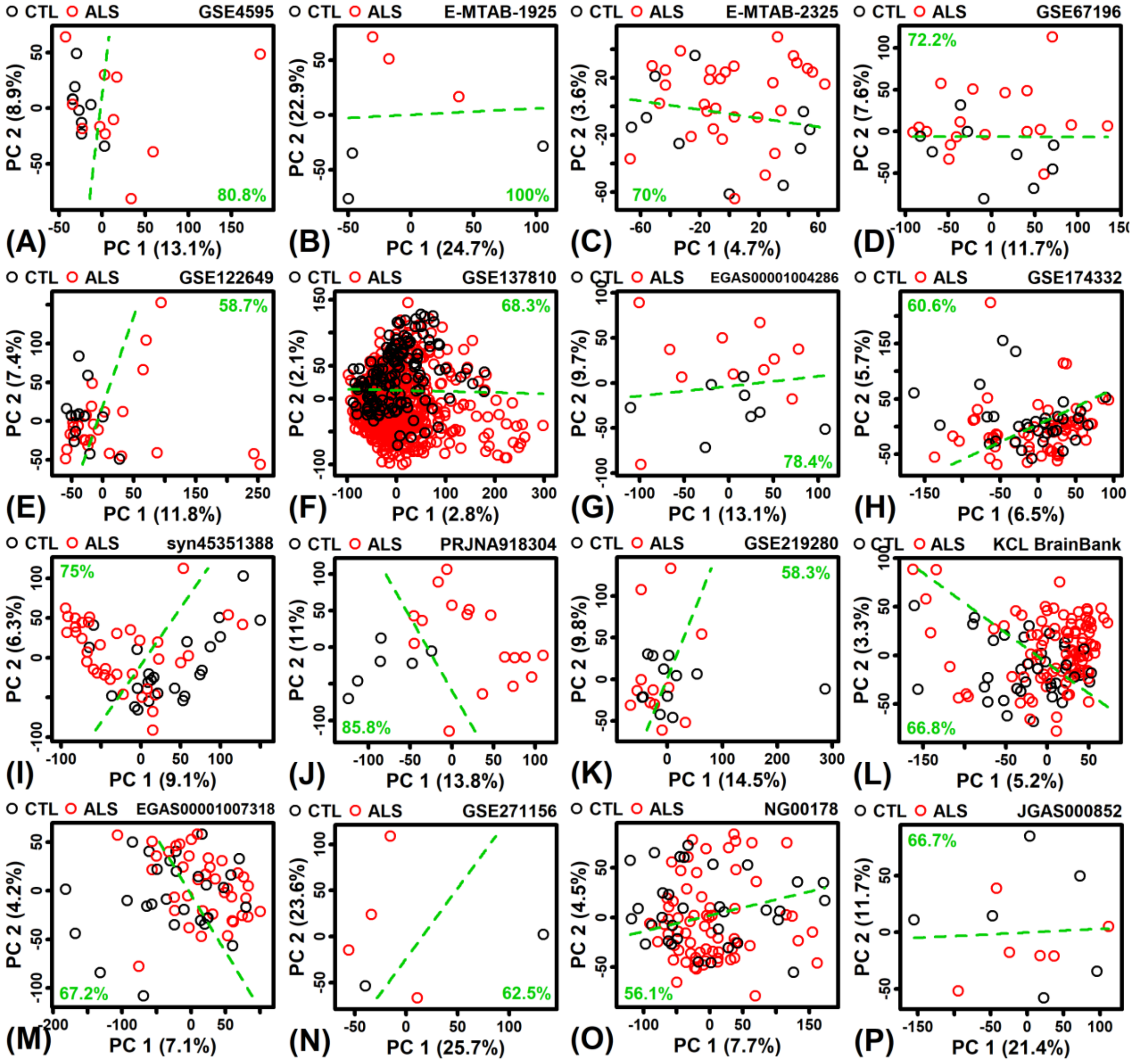

Figure S34. Principal component (PC) plots (ALS vs. CTL samples).

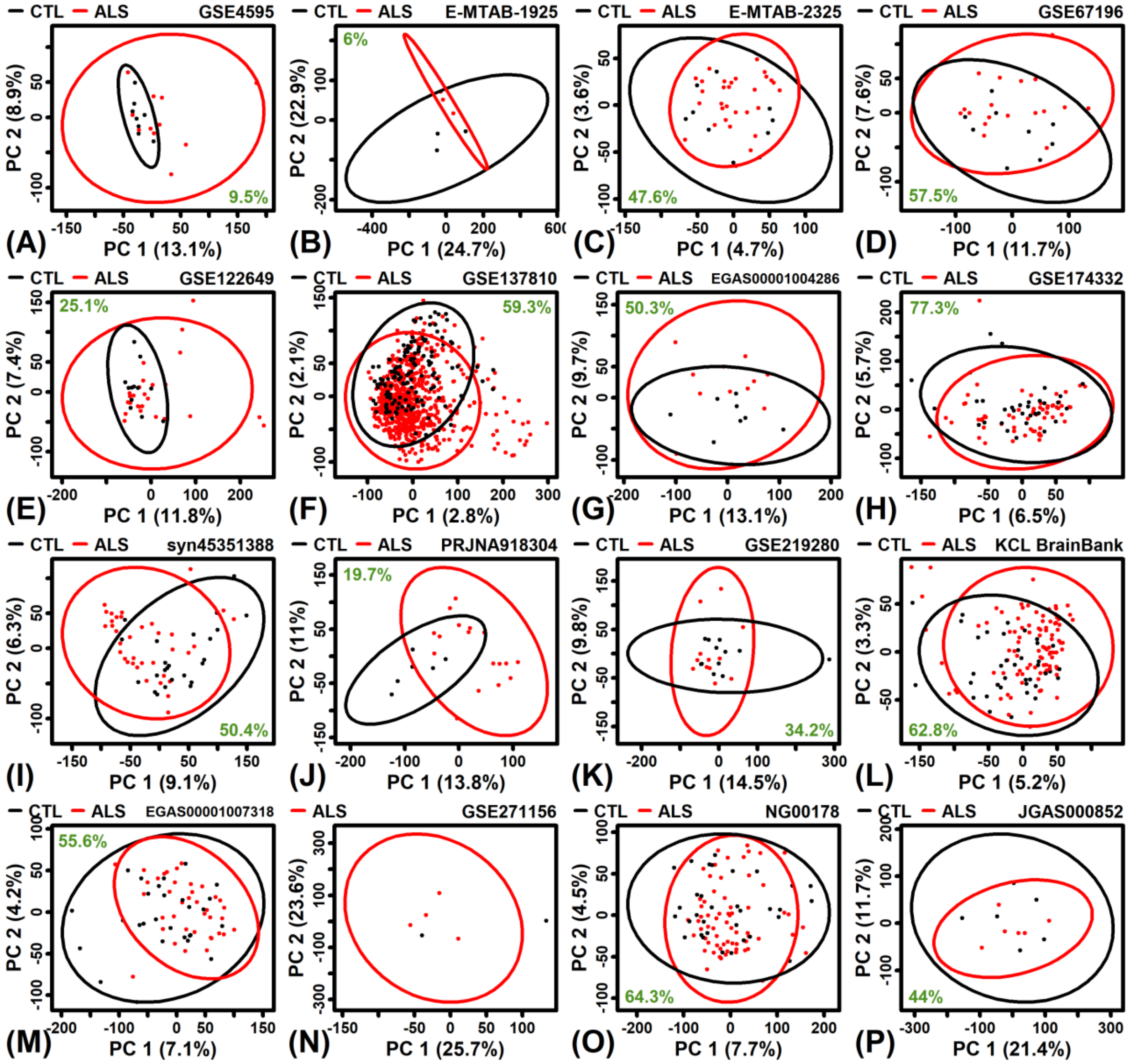

Figure S35. Normal-probability contours (ALS vs. CTL samples).

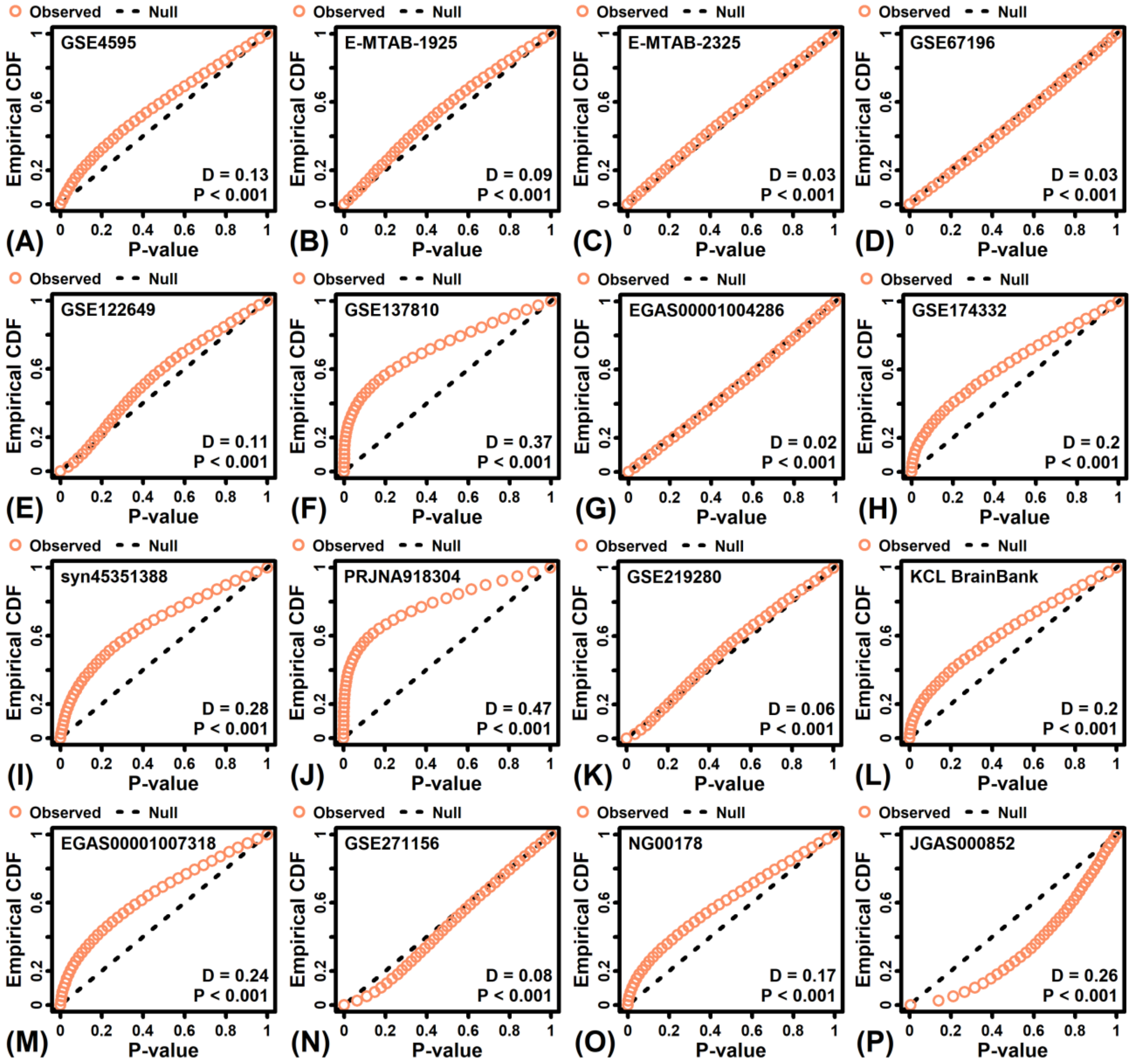

Figure S36. Raw p-value empirical cumulative distribution functions (ECDFs).

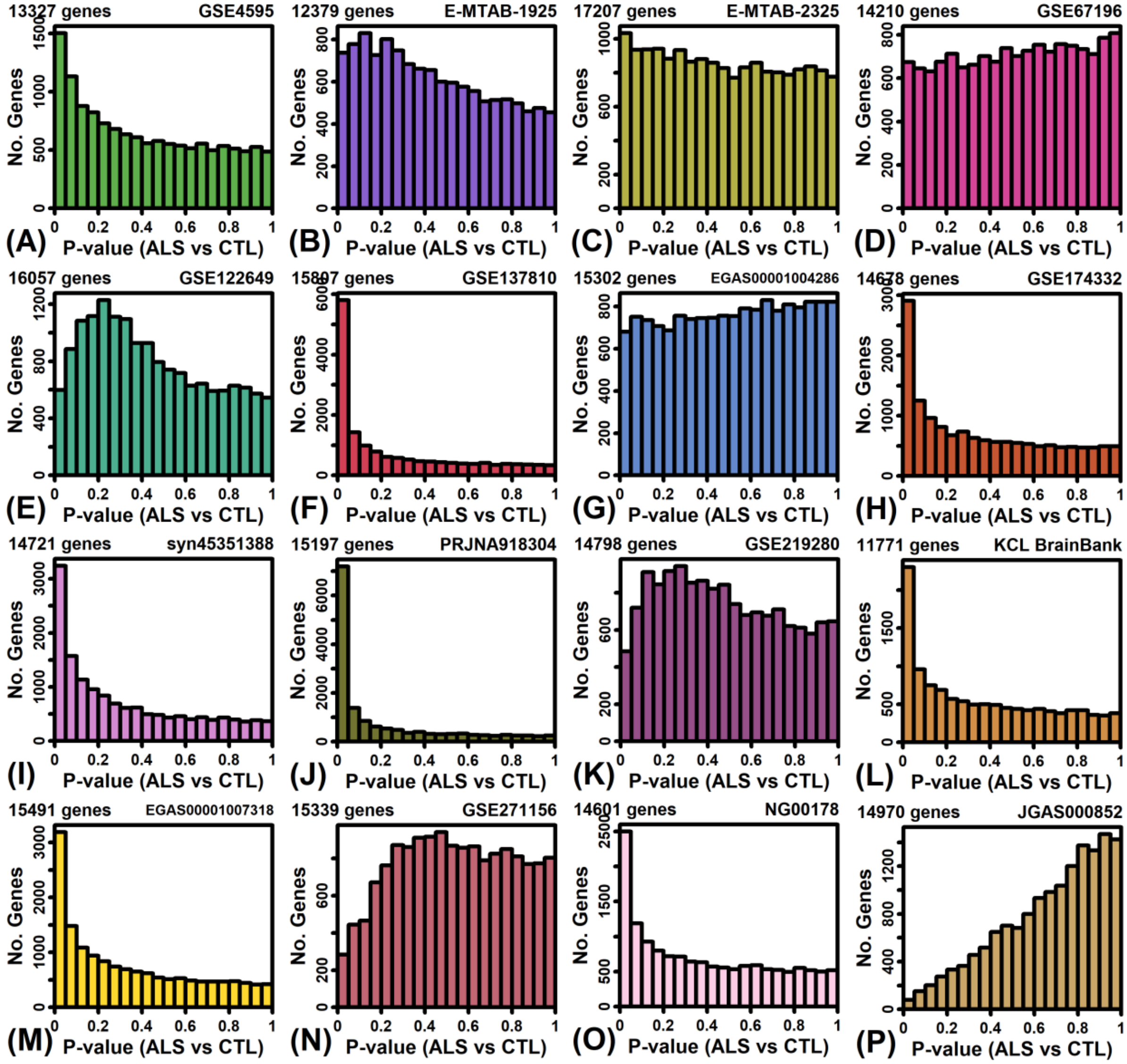

Figure S37. Raw p-value distributions.

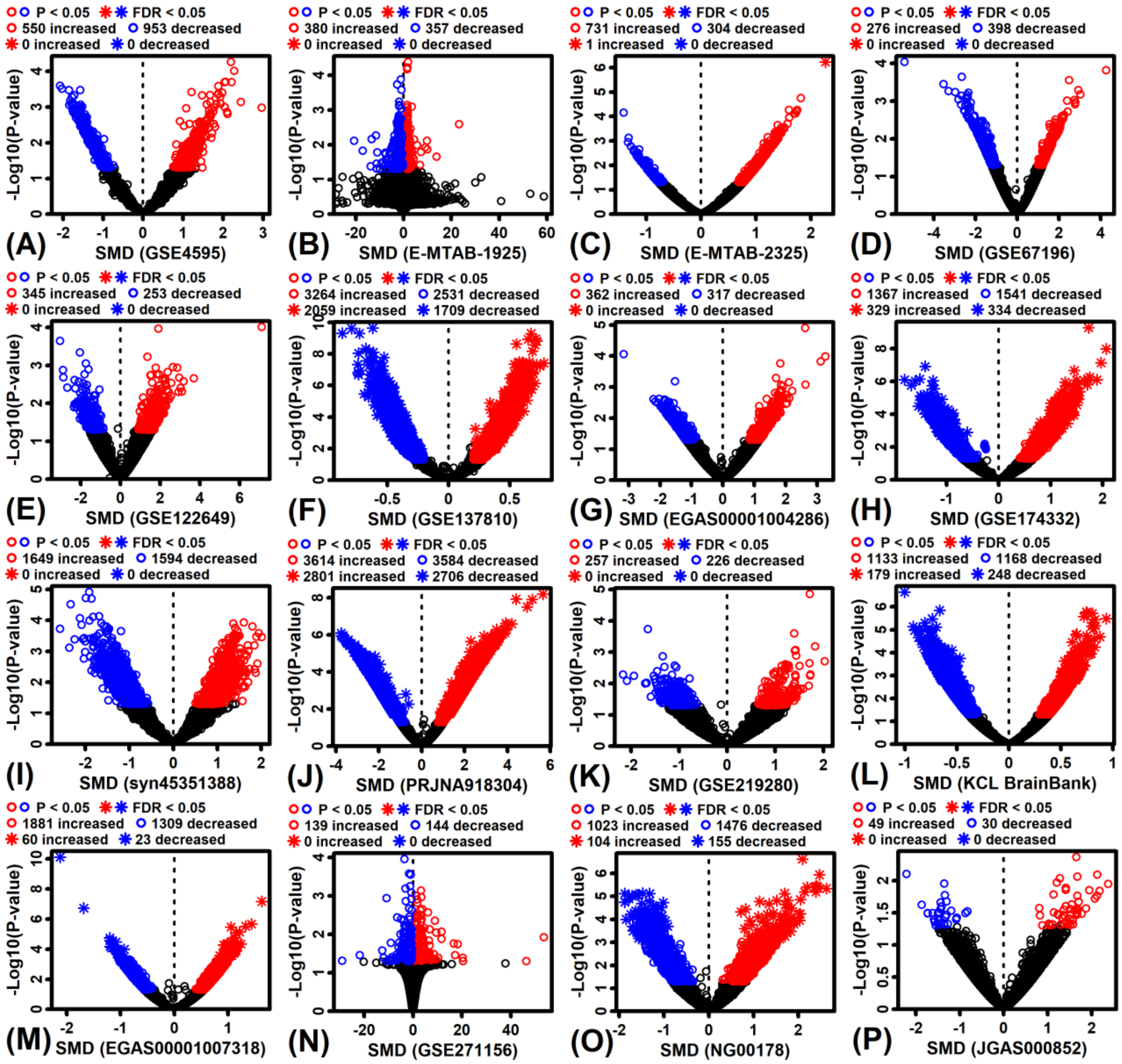

Figure S38. Volcano plots.

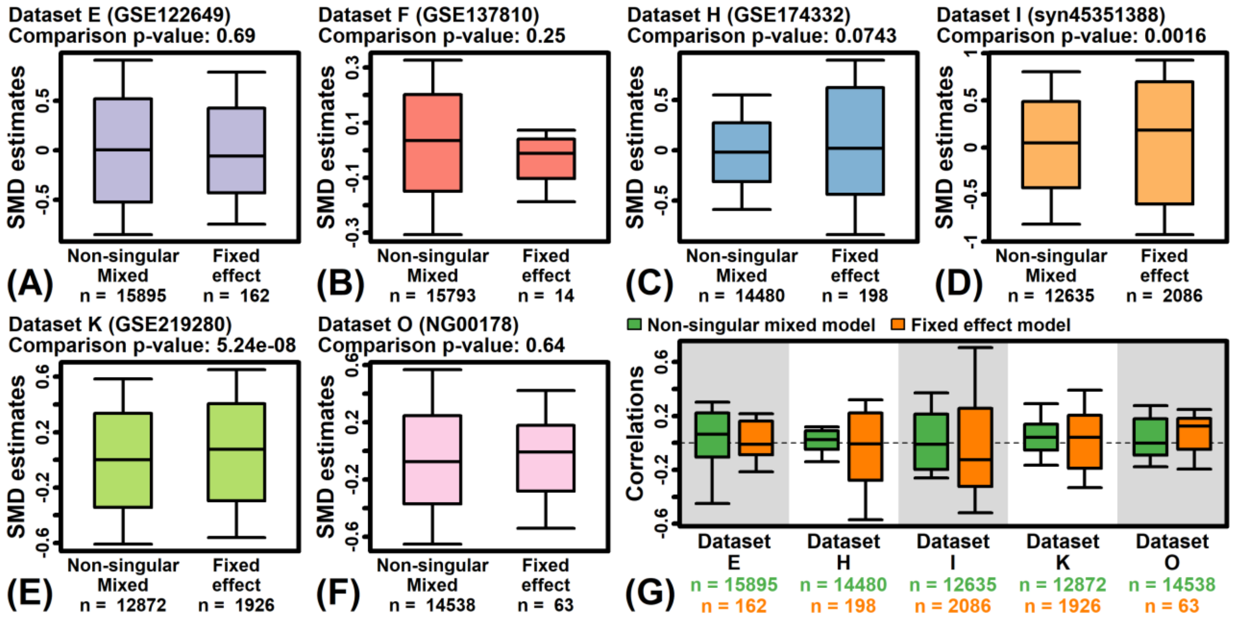

Figure S39. Comparison of SMD estimates by estimation method (Datasets E, F, H, I, K, O).

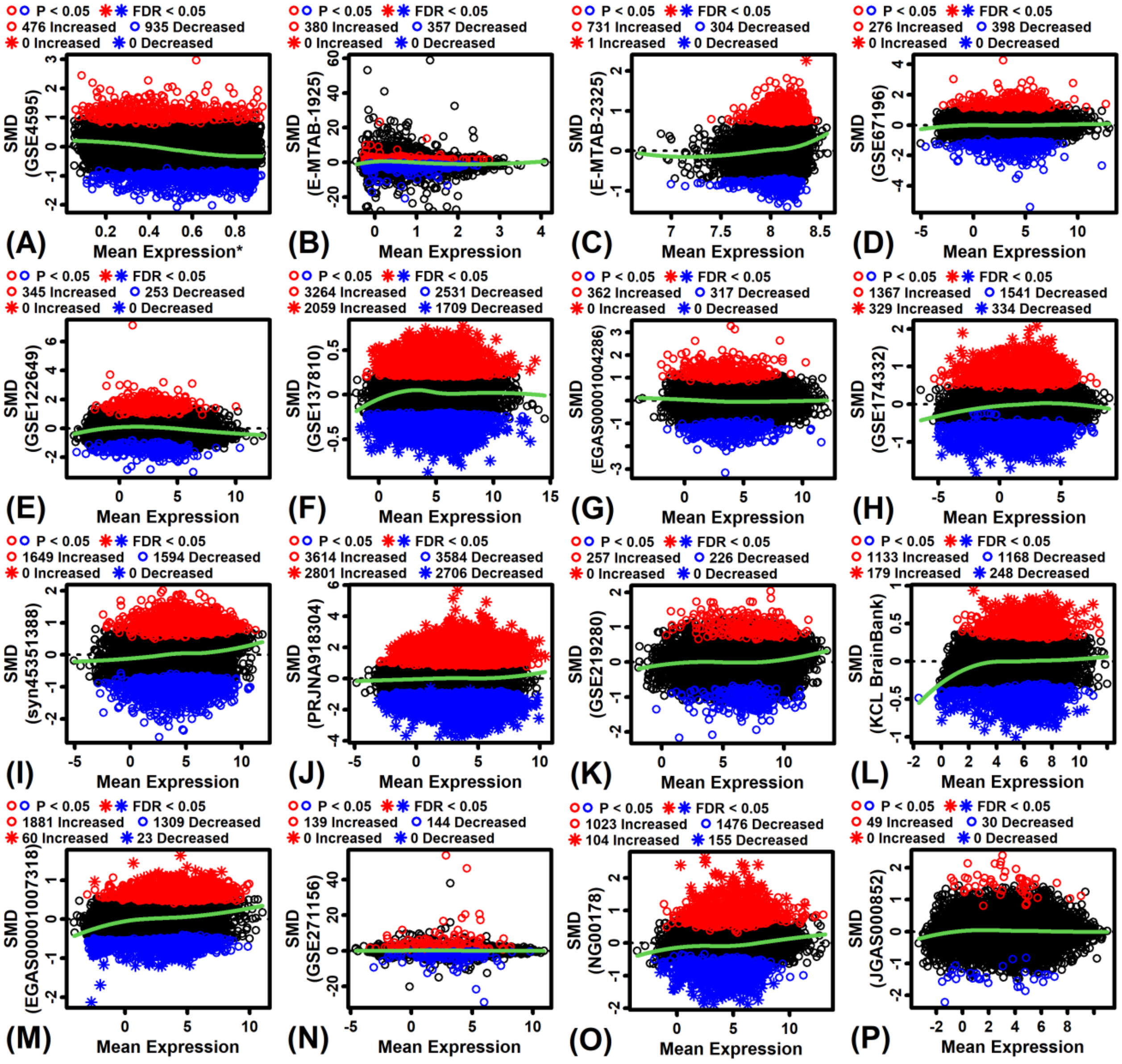

Figure S40. MA plots.

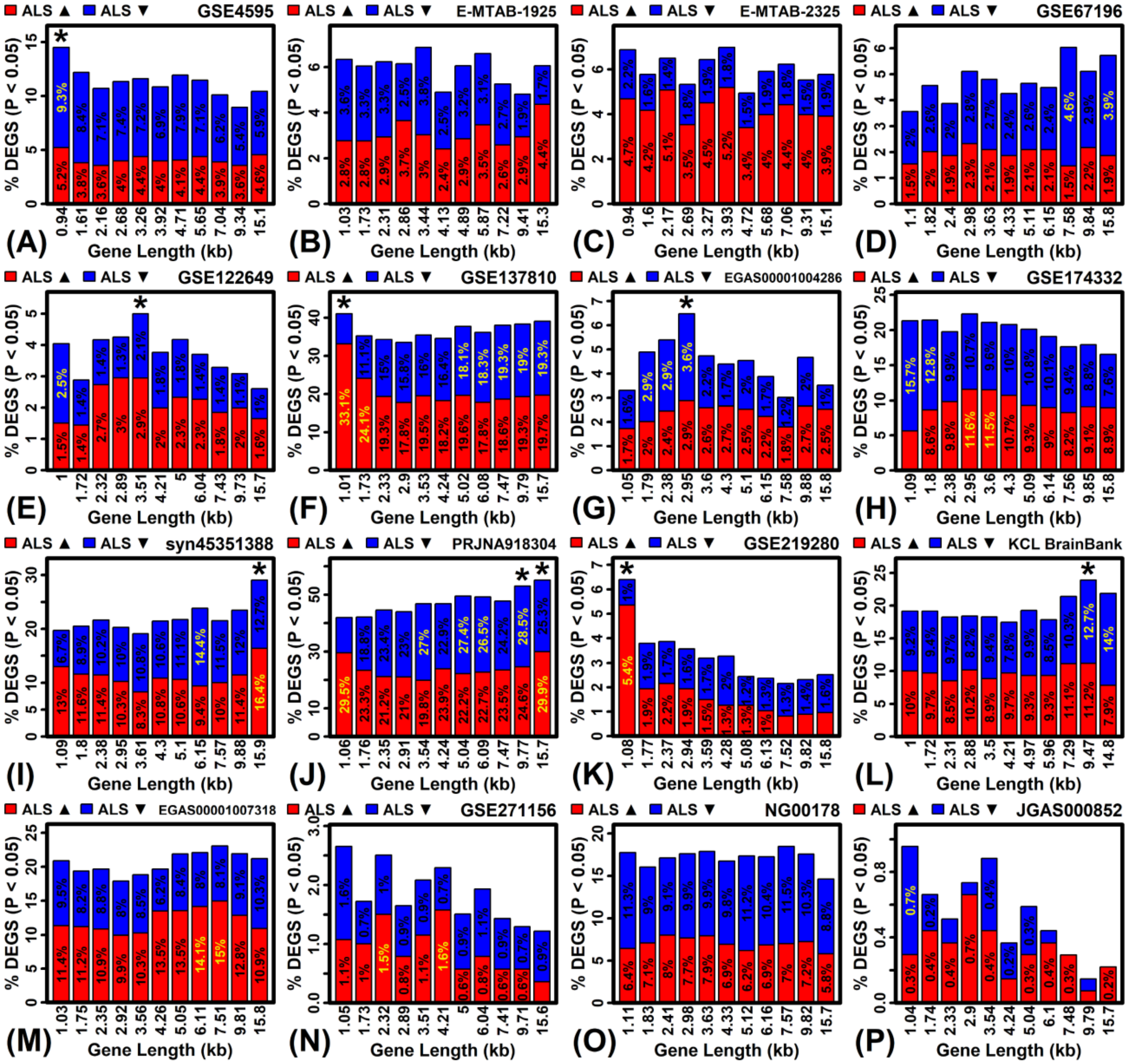

Figure S41. Gene length and differential expression.

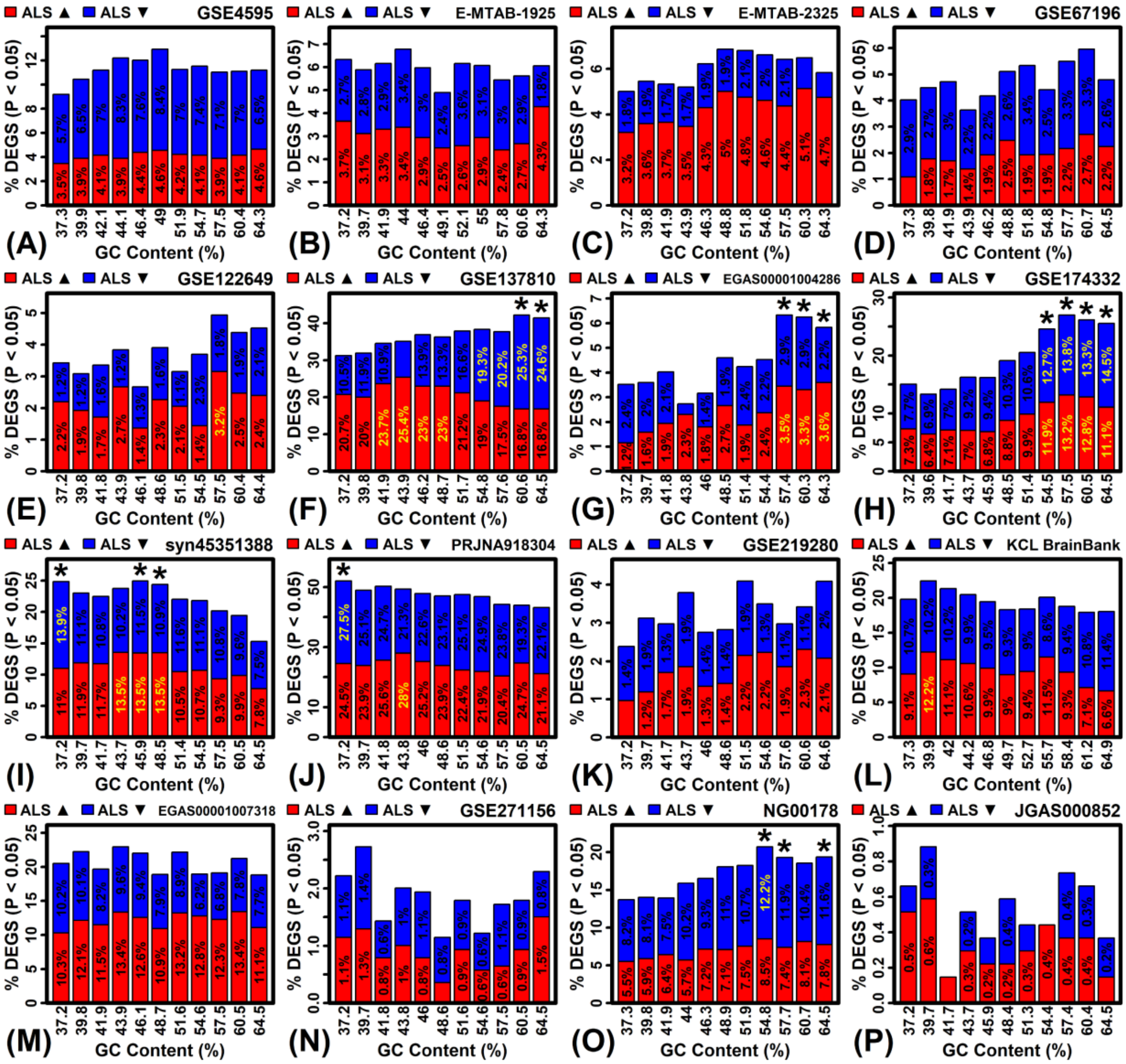

Figure S42. GC content and differential expression.

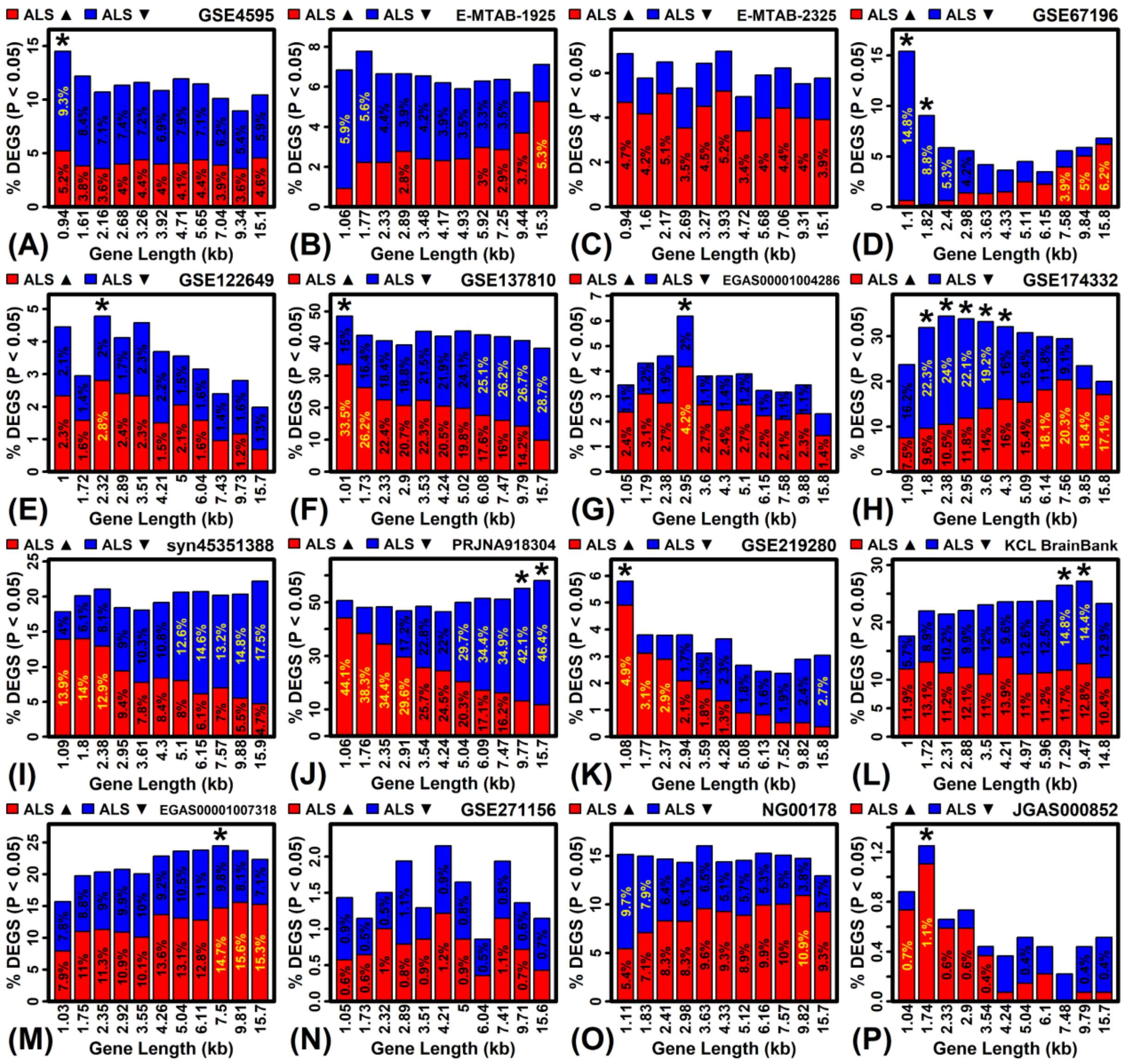

Figure S43. Gene length and differential expression (alternative normalization).

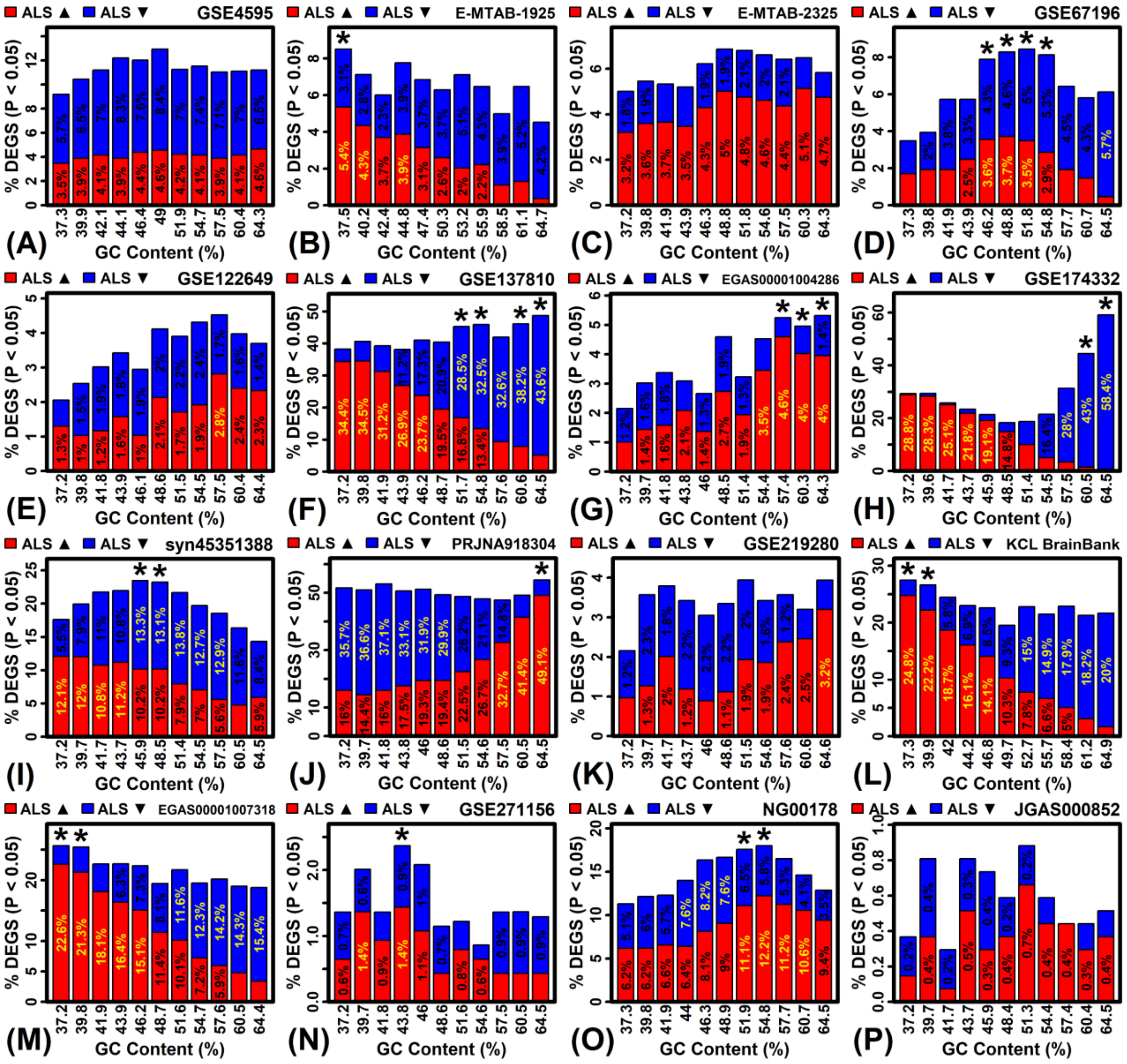

Figure S44. GC content and differential expression (alternative normalization).

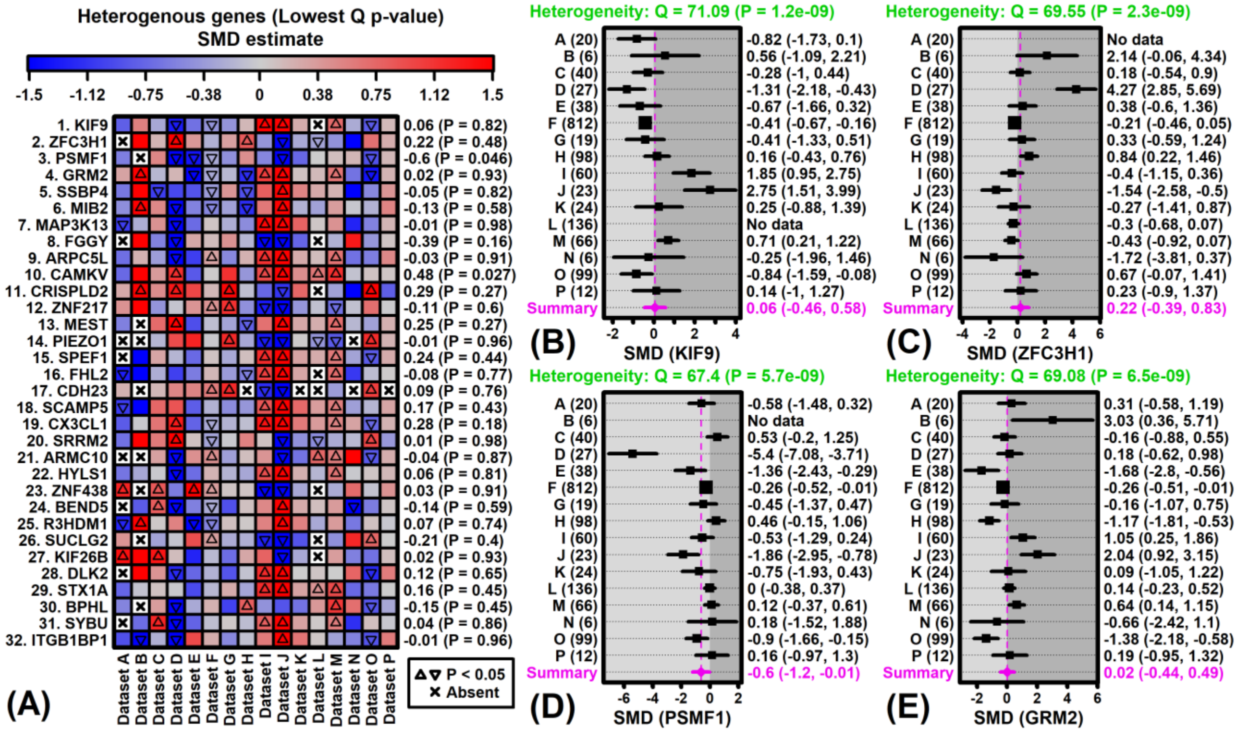

Figure S45. Heterogeneous genes.

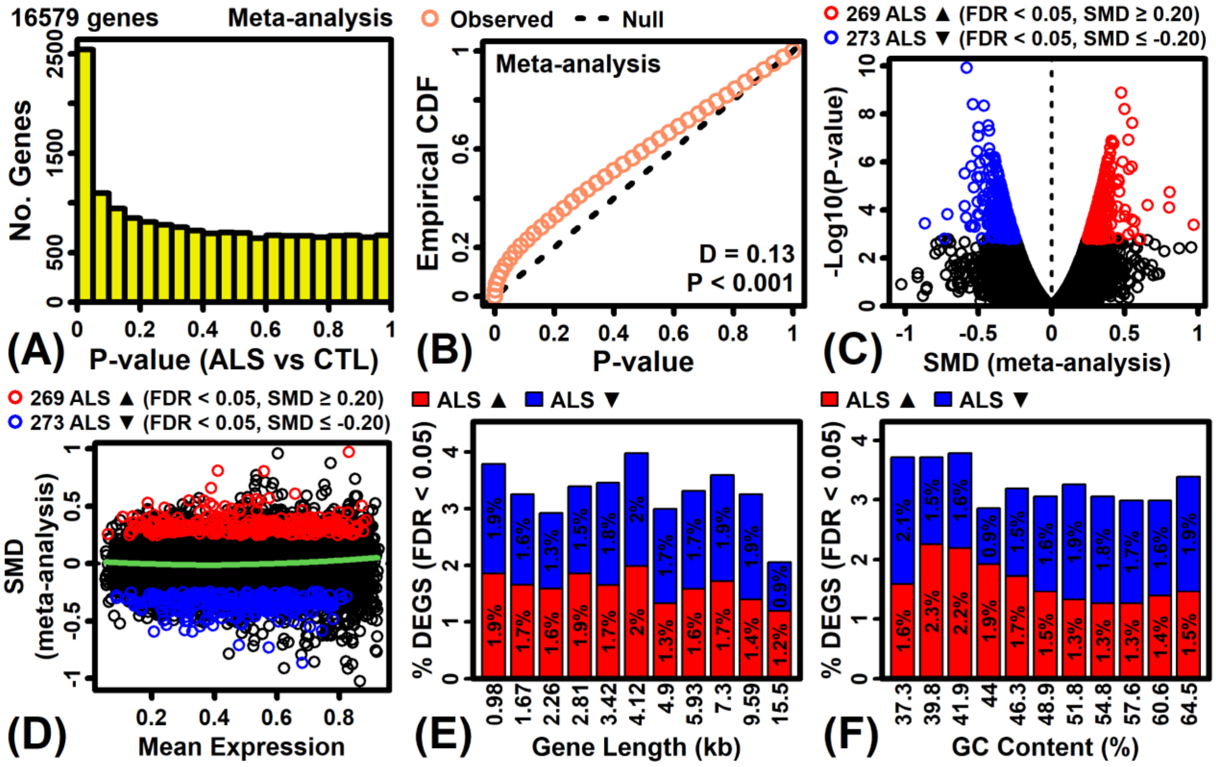

Figure S46. Meta-analysis differential expression analysis.

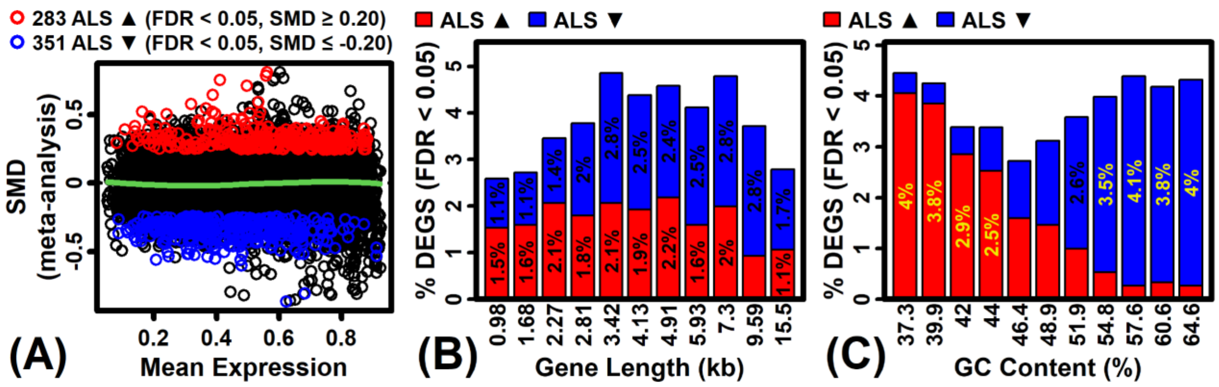

Figure S47. Meta-analysis differential expression analysis (alternative normalization).

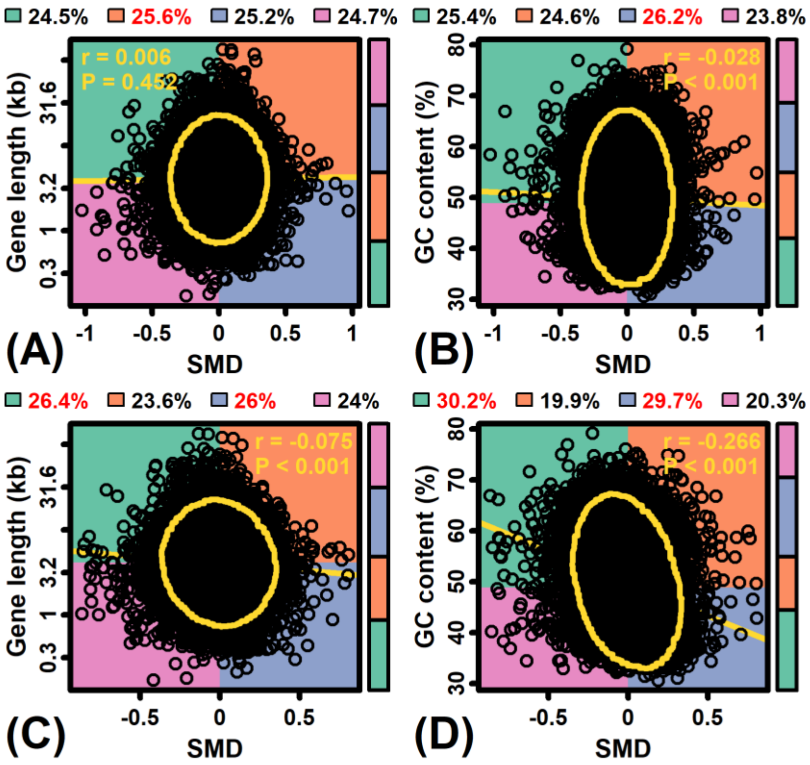

Figure S48. SMD versus gene length (kb) and GC content (%) scatterplots.

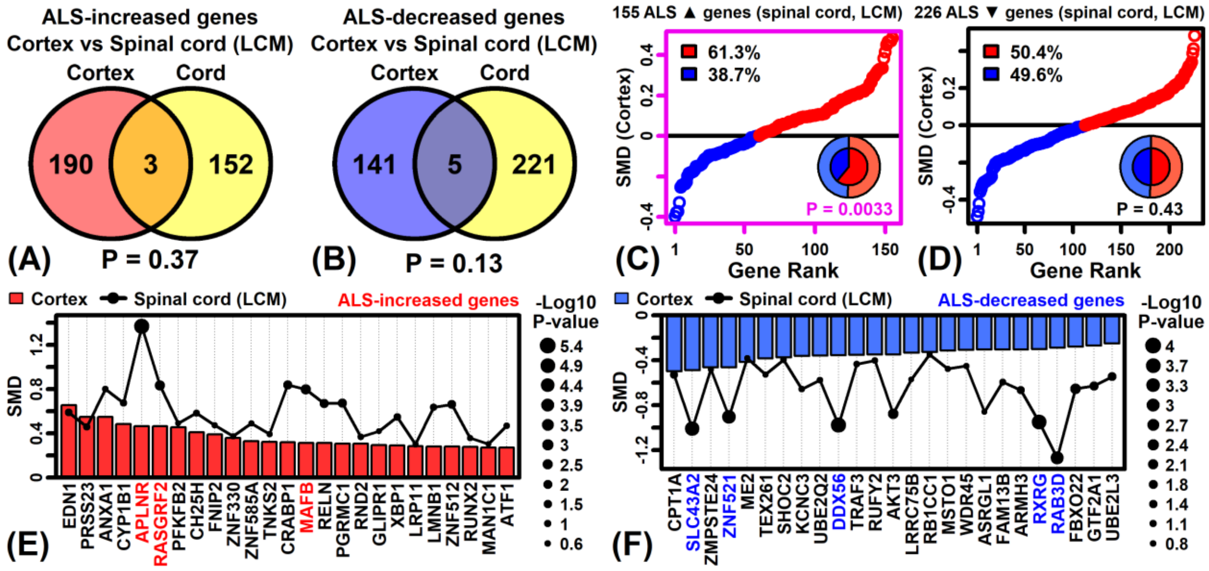

Figure S49. Comparison to DEGs from LCM-dissected spinal cord motor neurons meta-analysis (Swindell 2025, Frontiers in Genetics 15:1385114).

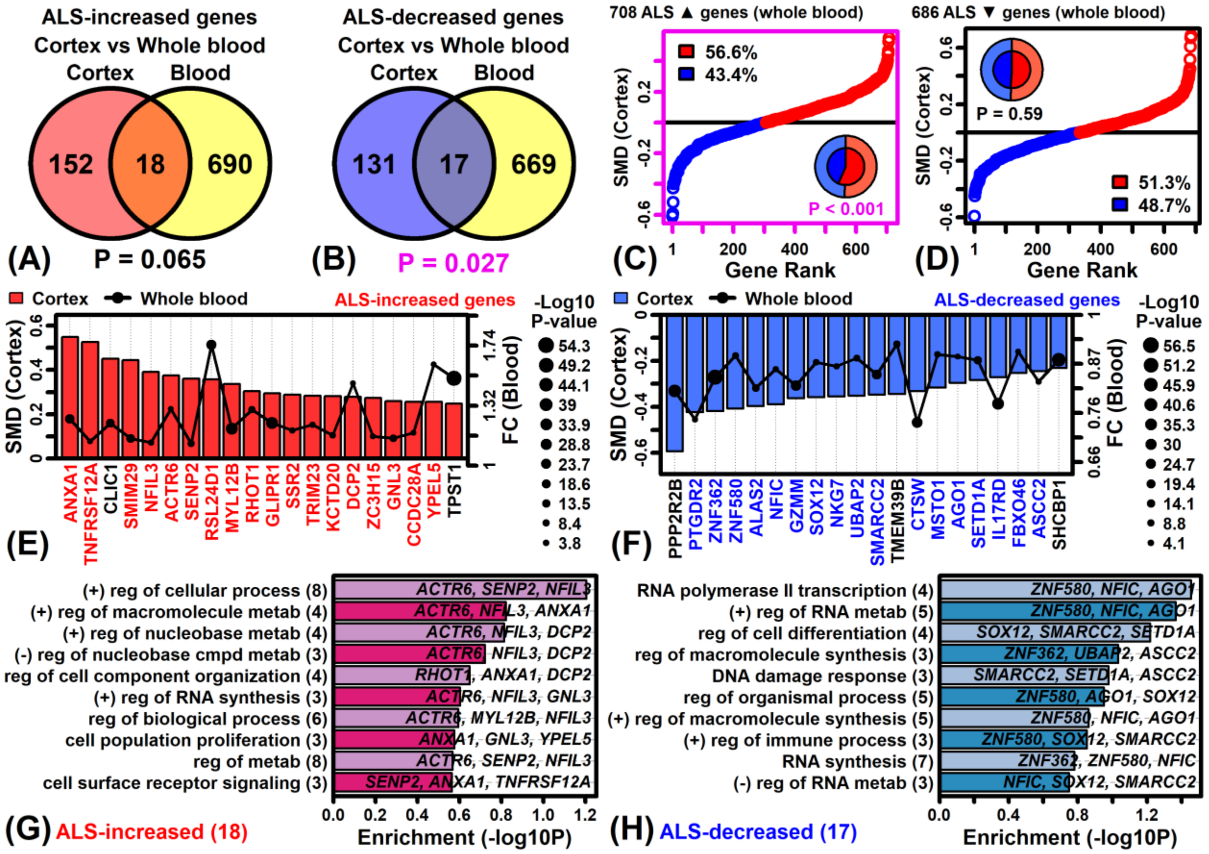

Figure S50. Comparison to DEGs from whole blood (Swindell et al. 2019, J Transl Med 17:170).

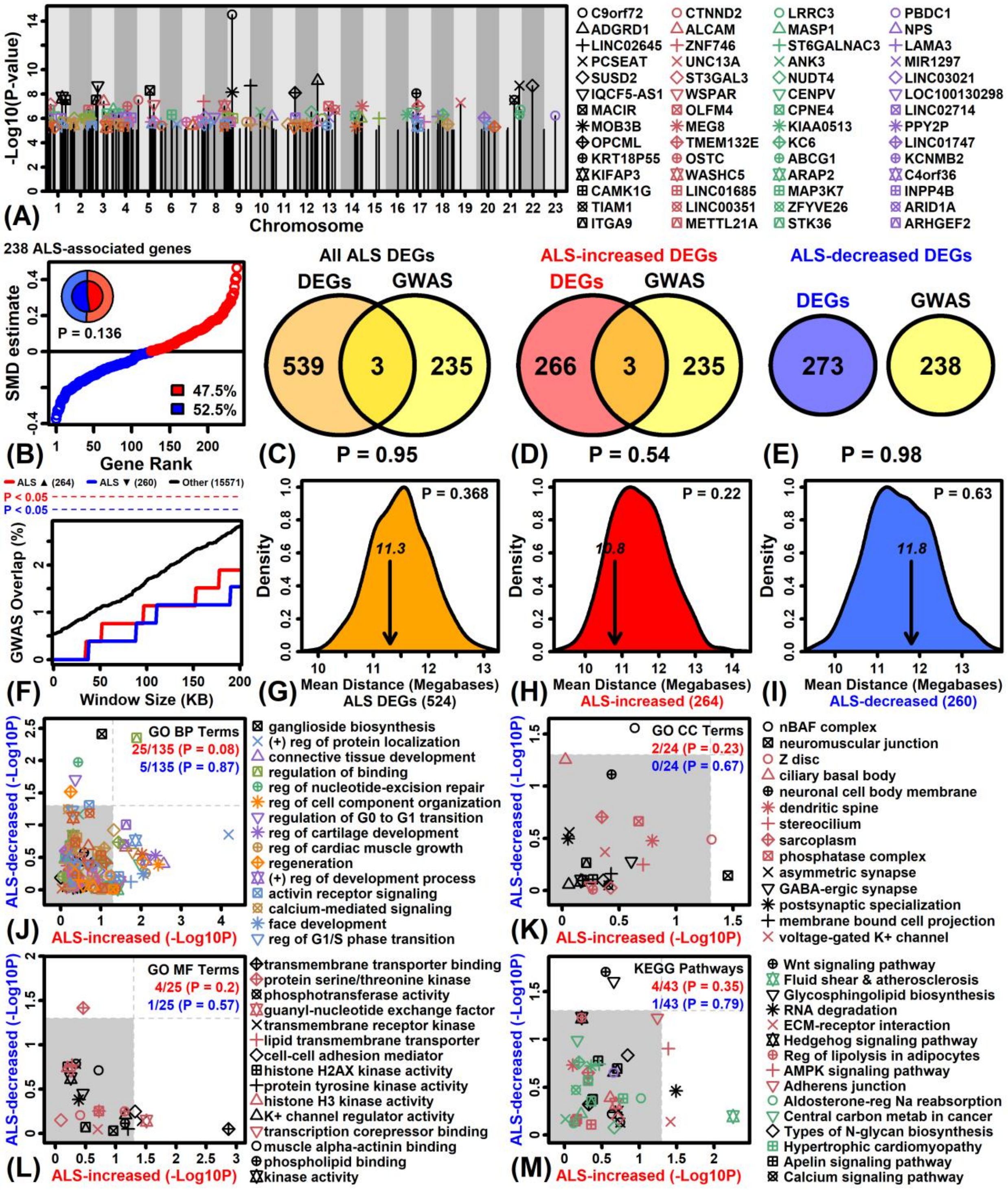

Figure S51. Overlap between DEGs and ALS-associated genes (NHGRI-EBI GWAS catalog).

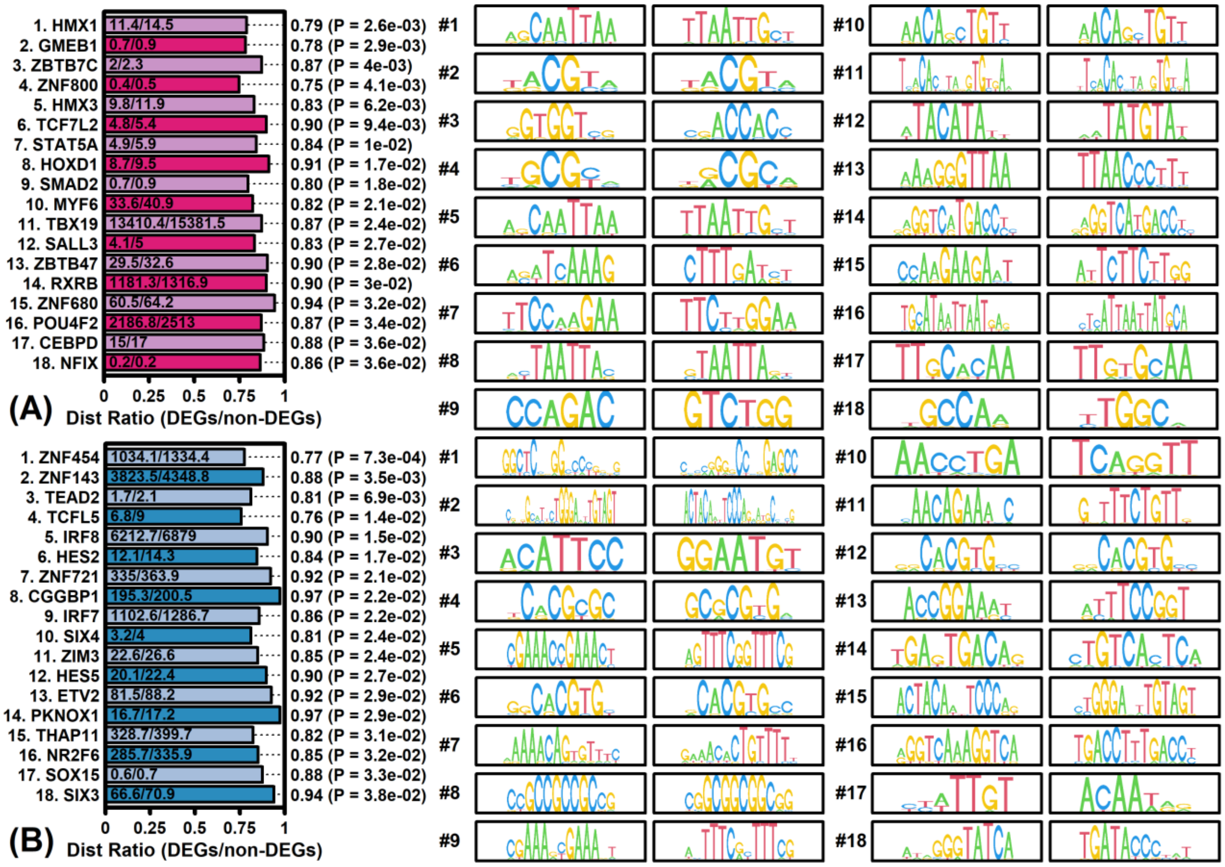

Figure S52. Transcription factor (TF) binding sites located near DEGs.
